# BanglaHateCorpus: A large-scale bangla benchmark for hate speech classification and a transformer-based deep learning method

**DOI:** 10.1371/journal.pone.0361339

**Published:** 2026-11-04

**Authors:** Anika Tasnim Rodela, Mahbub E Sobhani, Sakif Naieb Raiyan, Mohammad Nurul Huda, Dewan Md. Farid

**Affiliations:** 1 Department of Information and Communication Technology, Bangladesh University of Professionals, Dhaka, Bangladesh; 2 Department of Computer Science & Engineering, United International University, Dhaka, Bangladesh; 3 Department of Computer Science and Engineering, BRAC University, Dhaka, Bangladesh; 4 Department of Computer Science and Engineering, Bangladesh University of Engineering & Technology, Dhaka, Bangladesh; 5 Department of Computer Science & Engineering, Southeast University, Dhaka, Bangladesh; University of Naples Federico II: Universita degli Studi di Napoli Federico II, ITALY

## Abstract

Hate speech on social media refers to content that threatens, attacks, or dehumanizes people or groups based on specified characteristics, thereby eroding trust, worsening societal divisions, and causing psychological harm to the targeted communities. In digitally connected South Asian societies, strong in-group bonds and online inter-group hatred can rapidly lead to real-world violence, a risk intensified by the proliferation of Bangla texts, highlighting the urgent need for robust detection in this low-resource language. Previous studies have mostly focused on building their datasets by scraping various sources, without emphasizing standardized quality annotation or ensuring classification accuracy. To address these gaps, we introduce BanglaHateCorpus, a unified corpus of 168K examples consolidated from eight public sources, rigorously de-duplicated and annotated through a two-stage, human and LLM-assisted pipeline. Building on this benchmark, we propose BanHateFormer, a novel deep-learning model that embodies state-of-the-art transformer components, and replaces the conventional feed-forward blocks with a Mixture-of-Experts layer followed by a binary classification head. BanHateFormer achieves state-of-the-art classification results, as assessed by accuracy, precision, recall, and macro F1-score with approximately 94.74 times fewer parameters than comparable models, achieving a 91.33% macro F1-score on the BanglaHateCorpus dataset and 78.96% on the transliterated BanTH benchmark. BanHateFormer outperforms BanglaBERT and XLM-RoBERTa by up to 2.57% with three times fewer parameters, and improves macro F1-score by 3.38% on transliterated text, demonstrating strong performance on romanized inputs. BanHateFormer significantly surpasses existing transformer-based models in balancing memory efficiency and high-fidelity outputs, marking a breakthrough in hate speech classification with optimal space utilization.

## Introduction

Natural Language Processing (NLP) based hate-speech classifiers automatically differentiate between “hateful” content, which includes slurs, hostility, or calls for violence led at protected groups, and “non-hateful” posts that express neutral or benign opinions. The need for adequate systems to address abusive content has become increasingly obvious alongside the rapid growth of social media. Between July and December 2020, Twitter took action against 1,126,990 accounts for hateful conduct, marking a 77% increase compared to the prior six months. This data highlights the overwhelming amount of abusive content that necessitates automated enforcement [1]. Manual moderation, although accurate, toils to keep up with the volume of online content. A Pew Research Center [2] survey conducted in September 2020 with 10,093 U.S. adults revealed that 41% had personally encountered online harassment. Notably, nearly two-thirds of respondents under the age of thirty reported such incidents, highlighting both the widespread nature of abuse and its excessive consequences on younger users. While broad elimination policies may violate on free speech, 45% of Americans still support open expression online, even if it permits for some hateful content. Automated flagging can aid platforms in enforcing guidelines more efficiently, while also reducing false positives [2]. Recognizing this need, the European Commission’s voluntary “Code of Conduct on Countering Illegal Hate Speech Online” (May 2016) requires signatories (including Facebook, Microsoft, Twitter, and YouTube) to review and remove illegal hate speech within set timeframes and to uphold transparent appeal processes [3]. Yet periodic audits reveal uneven compliance across platforms, signaling that scalable technical solutions must complement policy measures [3]. For Bangla, a language spoken natively by over 242 million people and as a second language by another 43 million worldwide, these challenges are compounded by rich morphology, a complex script, and diverse dialects [4,5]. Tailoring binary hate-speech classifiers to Bangla is therefore crucial to protect a quarter-billion-strong online community, ensuring safe and open digital discourse for its speakers.

The detection of hate speech in Bangla, leveraging deep learning and especially transformer architectures, has seen growing interest [6,7], with various studies contributing valuable datasets and models. For instance, efforts like BD-SHS by Romim et al. [8] provided a large-scale, hierarchically annotated corpus, and BN-Hate by Jahan et al. [9] offered a balanced dataset, both significantly advancing resource availability for the community. Similarly, domain-specific models like BanglaHateBERT [9] demonstrated the efficacy of targeted pre-training, achieving strong performance on native Bengali text. Furthermore, the challenge of transliterated Bangla, prevalent in social media, has been notably addressed by Haider et al. [10] with the introduction of BANTH, the first multi-label hate speech dataset for transliterated Bangla, which also established strong baselines using further pre-trained transformer encoders and explored novel LLM prompting strategies. While such resources and the application of multilingual models like MuRIL [11] show promise in handling script variations and achieving state-of-the-art results on specific transliterated datasets, they are not without limitations; for instance, the models benchmarked on BANTH [10] can be parameter-inefficient, its utility for direct Bangla processing can be affected if back-translation from transliterated forms is not semantically aligned, and the dataset itself, despite expert verification, has been noted to contain instances where the annotation was not rigorous and included mistakes. More recent explorations with emergent Large Language Models, such as Gemma-2B [12], have also indicated considerable potential for achieving high accuracy in various Bangla hate speech and text classification tasks. While prior research encompassing sophisticated ensemble techniques for improved accuracy and explainability (e.g., DeepHateExplainer [13]), broader analyses across multiple Indian languages including Bangla that contribute new datasets [14], and the development of detailed taxonomies for specific violence types such as sectarian expressions [15] has significantly enriched the field, these pioneering efforts simultaneously underscore persistent underlying issues. For example, advanced ensemble models, despite their enhanced performance, frequently grapple with the challenge of increased computational demands and complexity. Moreover, even with detailed taxonomies for specific hate phenomena, existing models like BanglaBERT [16] can exhibit limitations in accurately classifying highly granular categories, and cross-lingual studies consistently highlight the pervasive problem of data imbalance and the scarcity of truly comprehensive, standardized resources for individual low-resource languages like Bangla. Notwithstanding these advancements, the broader landscape is often characterized by a fragmentation of resources; datasets, while individually valuable, frequently vary in their annotation schemes, scope, and overall size (e.g., Saha et al. [17] focusing on communal violence, or Raihan et al. [18] on transliterated offensive language), which can impede the development of models possessing broad generalizability. Moreover, while domain-adapted models like BanglaHateBERT are effective, they can still be parameter-heavy, and the systematic creation of a truly unified, large-scale benchmark through a rigorous, multi-stage annotation process that synergizes both human linguistic expertise and the nuanced judgment of LLMs has remained largely unaddressed. Additionally, the development of highly efficient, yet powerful, monolingual transformer-based architectures that integrate cutting-edge components specifically optimized for performance on such a unified benchmark presents a compelling, open research avenue. Consequently, many existing approaches, despite achieving commendable results on their specific datasets or tasks [19,20], often do not culminate in a single, cohesive, and quality-assured resource designed to tackle these widespread inconsistencies and resource limitations across the diverse spectrum of Bangla hate speech. Addressing these multifaceted challenges of resource fragmentation, annotation variability, and the quest for efficient yet robust models, this study therefore undertakes a foundational initiative: the meticulous construction of a unified, large-scale benchmark dataset and the introduction of a novel, parameter-efficient deep learning architecture specifically tailored for Bangla hate speech detection.

Several challenges have been identified in the domain of Bangla hate speech classification, particularly regarding the trade-offs between computational efficiency and enhanced classification accuracy. Moreover, the domain suffers from a lack of a precisely annotated, large-scale corpus and the underutilization of modern transformer components to build state-of-the-art monolingual deep learning model. The objective of this study is to recognize these limitations and establish an exhaustive baseline that will serve as a foundation for forthcoming research. To this end, we have developed an extensive parallel corpus by carefully selecting and merging existing datasets and by rigorously annotating all data according to international standards through a multi-stage strategy. We leverage both human expertise in linguistic nuances and the capabilities of state-of-the-art large language models to facilitate this annotation, thereby reducing the required time and effort. Additionally, we introduce BanHateFormer, a monolingual deep-learning model that achieves state-of-the-art performance with fewer parameters than existing baselines. We also scrutinize whether incorporating a mixture-of-experts mechanism further enhances model performance, and we also evaluate the robustness of BanHateFormer on transliterated Bangla text. BanHateFormer utilizes rotary positional encoding and self-attention to generate contextualized vector representations, which are then processed by a mixture-of-experts layer to grasp global dependencies. Through this persistent refinement, the model achieves remarkable efficiency while keeping high fidelity in hate speech classification. The contributions of our study are summarized below:

- We propose the BanglaHateCorpus, containing 168K hate and non-hate instances, created by carefully integrating diverse existing benchmark datasets.
- A pragmatic annotation process is tailored to leverage human expertise and LLM capabilities to ensure accurate and consistent labeling across a wide range of linguistic aspects.
- We have introduced BanHateFormer that incorporates rotary positional encoding, SwiGLU activation, RMSNorm, and a mixture-of-experts layer to achieve state-of-the-art performance with significantly fewer parameters than existing transformer-based baseline models.
- An exploration into the impact of training corpus size on the efficacy of BanHateFormer in classifying text within Bangla hate speech text is conducted, shedding light on its potential contributions in the realm of hate speech classification.
- We perform an in-depth analysis of the mixture-of-experts layer to quantify its benefits over traditional feedforward networks in capturing global dependencies.
- The empirical results of the proposed BanHateFormer have been compared with the baselines of the Bangla hate speech classification task. This comparison exhibits its superiority, as it outperforms the previous state-of-the-art performance while using approximately 94.74× fewer parameters. This demonstrates its remarkable capabilities across various Bangla natural language classification tasks.

The following sections of the paper are organized as follows: Sect. Related Work surveys existing research in hate speech classification and analysis, underlining transformers, and deep learning-based techniques along with their inherent limitations. The details of the corpus creation and annotation process are elaborated upon in Sect. Corpus Creation. Sect. Proposed Method outlines the methodology and architecture of BanHateFormer, a monolingual transformer-based method for efficient Bangla hate speech classification, covering its workflow throughout the whole network. Sect. Experimental Analysis presents a comprehensive empirical evaluation, experimental settings, evaluation metrics, and an ablation study, with results that benchmark BanHateFormer’s performance against state-of-the-art methods and provide qualitative insights. Finally, Sect. Conclusion and Future Work summarizes our findings, discusses the implications for memory-efficient NLP, and outlines promising avenues for future inquiry.

## Related work

The domain of online hate speech detection has become an area of widespread research attention, attracting significant interest and contributing to the development of innovative methodologies and datasets. Our extensive study aims to summarize key findings and showcase the evolving landscape of online hate speech detection methodologies across different language contexts, including Neural Network based [6,7] and Transformer based [10,11,13] techniques.

### Transformer-based methods

Transformer models, particularly BERT and its variants, have significantly advanced NLP, with a profound impact on hate speech detection in Bangla despite its low-resource status; more recently, Large Language Models (LLMs) and TinyLLMs have also emerged as promising tools as discussed by Albadi et al. [21]. Initial explorations often utilized multilingual models. For instance, Das et al. [11] demonstrated the utility of models like XLM-RoBERTa and MuRIL for Bengali and Romanized Bengali tweets from a new 10,000-tweet dataset, with MuRIL showing particular strength in joint training for transliterated text. However, a potential drawback of such models is that they may not fully capture all Bangla-specific nuances. Such constraints are increasingly understood within broader theoretical paradigms as well; rigorous PAC framework analyses indicate that learning with low-resource data involves inherent trade-offs in generalization error and label complexity that simply scaling standard models may not strictly overcome, as mentioned by Cao et al. [22]. Building on this, Ghosh and Senapati [14] further noted MuRIL-BERT’s good performance for Bangla within a broader analysis of Indian languages using existing and newly created datasets (HASOC, HS-Bangla, Assamese, Bodo), yet concurrently highlighted persistent challenges like dataset imbalance. The adaptability of multilingual models was also seen in the work of Alam et al. [23], where M-BERT achieved a 0.80 macro F1-score on code-mixed Tamil-English caste and migration hate speech; nevertheless, it reportedly struggled with the inherent complexities of the code-mixed nature and data imbalance. To address the aforementioned limitations of generic multilingual models, subsequent research has shifted towards Bangla-specific transformers. Jahan et al. [9], for example, introduced BanglaHateBERT, which was retrained on a large (1.5 million posts) offensive corpus and a new 15K balanced hate speech dataset. This domain-specific model consistently outperformed generic BERTs (94.3% accuracy), underscoring the value of such retraining. While effective, the breadth of subtle hate forms covered by even extensive retraining remains an ongoing area for expansion. Furthermore, applications to specific hate contexts like violence have also leveraged transformers. Saha et al. [17] introduced the Vio-Lens dataset (6,046 YouTube comments) for communal violence, where BanglaBERT performed best (Macro F1 0.7879). Concurrently, Tasnim et al. [15] presented the BASED dataset (13,000 texts) for sectarian expressions, noting BanglaBERT’s difficulties with highly granular (16-class) categorization. Moreover, shared tasks on the Vio-Lens dataset, such as BLP-2023 Task 1, saw contributions using BanglaBERT [19], Hierarchical-BERT [20], and MLM-refined ensembles [24]. These efforts, while achieving notable F1-scores (around 0.72–0.74), collectively highlighted persistent issues of dataset imbalance and the nuanced classification challenges inherent in specific violence types. More sophisticated appro aches have involved ensembling and explainability. Karim et al. [13], for instance, demonstrated that an ensemble of transformers (Bangla BERT, mBERT, XLM-R) on an 8,000 + example Bengali Hate Speech Dataset outperformed individual models (88% F1-score) and, crucially, provided human-interpretable explanations via sensitivity analysis and LRP. Extreme ensemble usage remains prominent, as evidenced by recent 2025 shared tasks introduced by Hasan et al. [25] where some leading teams relied on heavy multi-task transformer ensembles and weighted probabilistic fusions by Alam et al. [26] to overcome fragmented sub-datasets. The primary trade-off with such ensemble methods, however, is significantly increased computational cost and parameter bloat, motivating the need for more parameter-efficient standalone architectures. In parallel, linguistic diversity, especially transliteration and code-mixing, remains a key challenge. Raihan et al. [18] introduced the TB-OLID dataset (5,000 comments) and found English pre-trained task-specific models (fBERT, HateBERT) superior, suggesting standard Bangla models may struggle with Latin script. Complementing this, Haider et al. [10] released BANTH, a large (37,350 comments) multi-label dataset for transliterated YouTube text. Their further pre-trained encoders achieved SOTA results, though dataset imbalance and specific label classification (e.g., “Personal Offense”) remained challenging aspects. The capabilities of LLMs and TinyLLMs are also increasingly being explored. Dehan et al. [12] showed that fine-tuned Gemma-2B excelled in Bangla hate speech and sarcasm detection on diverse datasets (including a ~ 4k HS dataset), often outperforming larger or Bangla-specific models. Beyond pure classification, the capacity of LLMs to bridge semantic gaps via targeted data augmentation serves as an effective mechanism across diverse domains, including multimodal ranking and text analysis, as shown by Li et al. [27]. More recent investigations have evaluated LLMs using multi-task LoRA fine-tuning for severity and targeted entity identification, noting that while LLMs adapt well to low-resource settings, culturally-grounded pretraining retains a qualitative edge which is demonstrated by Hasan et al. [28]. Furthermore, Haider et al. [10] also explored LLM prompting, with a novel translation-based approach outperforming zero-shot methods on their transliterated dataset; however, achieving robust prompting for nuanced hate detection is an ongoing pursuit. Lastly, the study by Babu et al. [29] employed BERT after translating Bangla to English, achieving 95% accuracy; yet, this was lower than their RNN-based models on the same translated data and inherently risks loss of original nuance as well as encountering synthetic generation artifacts known to disrupt the original structural calibration of the text, as mentioned by Yin and Wang [30].

### RNN and CNN-based methods

With the rise of deep learning, Recurrent Neural Networks (RNNs) and Convolutional Neural Networks (CNNs), frequently in hybrid configurations, gained traction for their ability to automatically learn features and capture sequential or local textual patterns. An early application for Bangla hate speech by Das et al. [6] introduced an attention mechanism with an RNN encoder-decoder, achieving 77% accuracy on a custom 7,425-comment dataset across seven categories, thereby outperforming standard LSTM/GRU. While pioneering for Bangla, the dataset size presented limitations for capturing the full diversity of hate speech. Subsequently, hybrid models like the Multichannel Convolutional-LSTM (MConv-LSTM) proposed by Karim et al. [7], when combined with their large pre-trained BengFastText embedding on a new 35,000-statement hate speech dataset (one of three they contributed), achieved an impressive 88.2% F1-score. A caveat, however, is that embeddings trained on formal text like BengFastText often struggle with the informal language prevalent on social media. Meanwhile, the efficacy of CNN-Bi-GRU architectures was highlighted by Roy et al. [31] for English offensive text (96.97% accuracy on 31,000 + tweets), suggesting architectural viability for other languages, provided appropriate linguistic resources are available. Further RNN applications targeted specific hate types. For example, Ahmed et al. [32] used an LSTM with Word2Vec for political hate in Bangla on a 1,980-text dataset, achieving 88.28% accuracy, though the small dataset size might limit broader generalization. In a significant dataset contribution, Romim et al. [8] introduced BD-SHS (50,200 + comments with three-level hierarchical annotation). Using Bi-LSTM with their custom InFormal Text (IFT) embeddings, they achieved a 91.0% F1-score for hate identification, proving the value of domain-specific informal embeddings. Nevertheless, even this large dataset posed challenges for accurately categorizing certain hate types, such as those related to’Religion’. The work by Mohi Uddin et al. [33], focusing on cyberbullying on a 94K fused dataset, found BiGRU to be their best deep learning model (90.63% accuracy); interestingly, this was surpassed by their classical ML stacking approach, indicating that for certain tasks or data configurations, advanced DL models do not always guarantee superior performance over well-tuned classical methods. RNNs also found utility in multimodal contexts. Debnath et al. [34], with their ExMute dataset of 6,199 Bangla/code-mixed memes, showed an LSTM-attention model achieving 0.60 accuracy for textual analysis, while multimodal models reached ~0.64 accuracy. The relatively limited improvement from simple fusion, however, pointed to the underlying complexities in effective multimodal integration for hate detection. Finally, Babu et al. [29] explored GRU and Attention models on a ~ 5,000 sample dataset translated to English, achieving high accuracies (up to 98.87%). While these results are strong, this approach of translating to a high-resource language risks obscuring original Bangla nuances and introducing translation artifacts. Collectively, these studies showcase RNN/CNN versatility but also consistently highlight ongoing challenges related to dataset specificity, the nature of informal language, and the intricacies of cross-lingual and multimodal complexities.

### Classical machine learning-based methods

Initial forays into automated hate speech detection predominantly utilized classical machine learning (ML) algorithms, which established important baselines and informed later deep learning efforts. These methods characteristically depend on manual feature engineering, such as Term Frequency-Inverse Document Frequency (TF-IDF), and standard classification algorithms. For Bengali, Sultana et al. [35] explored algorithms like Support Vector Machines (SVM), Naïve Bayes (NB), and Random Forest (RF) for detecting abusive comments from a dataset of 5,000 social media entries, finding SVM with TF-IDF features to be the most effective (85.7% accuracy). While demonstrating viability for binary classification, a common challenge with such approaches lies in scaling the feature engineering process for more nuanced or diverse hate speech types. Similarly, Romim et al. [36] presented a public dataset of 30,000 Bengali comments, where SVM (87.5% accuracy) again outperformed early deep learning models like LSTM and Bi-LSTM. The authors suggested that dataset imbalance and the unique nature of social media language might have favored the SVM in this instance; however, it’s also true that SVMs themselves are sensitive to feature quality and careful parameter tuning. The combination of richer text representations with ML classifiers was further explored by Papel et al. [37], who used a 30,000-message dataset. Their hybrid RF-SVM model, when paired with FastText embeddings, achieved a high accuracy of 96.85%. Despite this strong performance, the study acknowledged persistent difficulties in effectively minimizing false negatives, indicating a remaining need for more refined detection of subtle or insidiously phrased hate. In the related area of multi-class cyberbullying, Mohi Uddin et al. [33] evaluated ten ML models on a large fused dataset of 94,000 comments, with a hybrid stacking model (SGD + MLP + LR) proving superior with 99.34% accuracy. While impressive, the specific focus on cyberbullying and the particular dataset fusion strategy might present different generalization characteristics compared to broader hate speech detection. These studies affirm the utility of classical ML, particularly with robust features or sophisticated embeddings, or in resource-constrained scenarios. Nevertheless, their typical reliance on extensive, often language-specific feature engineering and potential limitations in capturing deeper semantic context largely motivated the subsequent shift towards more advanced neural network architectures.

Transformer-based models, despite their significant advancements in hate speech detection globally, often face challenges in low-resource languages like Bangla due to their reliance on extensive, high-quality annotated data and their typically large parameter counts which can hinder efficiency. Despite the valuable contributions from numerous researchers in creating datasets for Bangla (e.g.[6,8,10,17,18,33,36]), fragmented resources mark the landscape, and the systematic development of a unified, large-scale benchmark refined through a hybrid human-LLM annotation process remains largely unaddressed. To the best of our knowledge, no existing work introduces a comprehensive, consolidated Bangla hate speech benchmark dataset meticulously curated from diverse sources and refined via a multi-stage human and LLM-assisted annotation pipeline, nor has a dedicated monolingual transformer model been proposed that integrates state-of-the-art components like rotary positional encoding with a Mixture-of-Experts layer to achieve both high accuracy and significant parameter efficiency for this task. This distinctive combination of a rigorously developed benchmark corpus, BanglaHateCorpus, and a novel, efficient deep learning model, BanHateFormer, establishes the novelty of our approach in addressing these critical gaps in Bangla hate speech classification research.

## Corpus creation

In recent years, the availability of large‑scale corpora for harmful‑language tasks, such as hate speech detection, toxic comment classification, and cyberbullying detection–-has increased impressively for high‑resource languages like English, thanks to shared tasks and public benchmarks [40]. Nonetheless, for Bangla, there are fragmented and limited resources, hindering the development of robust automated moderation and analysis systems. To mitigate this gap, we used several publicly available high-quality Bangla datasets, two focused on cyberbullying and two on toxic comments, along with four dedicated hate speech collections. We combined cyberbullying and toxic comment datasets because these domains share equivalent linguistic patterns. Toxicity serves as a comprehensive category that includes profanity, insults, cyberbullying, and hate speech [41]. Empirical studies also demonstrate a substantial positive correlation between cyberbullying and cyberhate perpetration, both of which are worsened by toxic online disinhibition. This indicates that these problems often co-occur and reinforce each other [42]. Consequently, we introduce BanglaHateCorpus, a large‑scale, high‑quality Bangla hate‑speech corpus that extensively covers harmful language patterns and is designed to enhance the generalizability and robustness of Bangla NLP models for moderation, detection, and analysis.

Moreover, we observed persistent annotation errors in existing hate‑speech datasets depicted in Table 1. To remedy this, we adopt the concept of LLM as an annotator and judge [43] to refine and verify our labels. Overall, our approach creates a cohesive, reliable corpus poised to advance research in Bangla hate‑speech classification.

**Table 1:** Illustration of existing mislabelings and our corrected labels with justifications, following UNESCO [38], UN [39], and other reliable sources. Here, HS denotes *Hate Speech* and NHS denotes *Non-Hate Speech.*

Example	Existing Label	Corrected Label	Justification	Dataset Name
1: ফেতনা তোমরই তৈরি কর তুমি কয় ওয়ক্ত নমজ পর নিজের চিন্ত কর। (You are the ones who create the chaos; think about yourself and how many times you pray.)	NHS	HS	Target: Religion Form: Stigma, Scapegoating, Derogation Content: Religious misuse, Piety attack Impact: Humiliation, Exclusion	BD-SHS
2: শল যেনো টক পয়স নই চুল কটর! (It seems like that fellow doesn’t have any money for a haircut.)	HS	NHS	Target: Individual Form: Finance / Appearance Content: Slur, Derogation Impact: Personal abuse; non-protected	BD-SHS
3: ওর ২ হত কট হোক। আওয়মী লীগের একট কর্মীও যেন জীবিত ন থকে। (May his two hands be cut off. May not a single Awami League worker remain alive.)	NHS	HS	Target: Individual+Political Form: Extermination incitement Content: Mutilation & mass killing Impact: Extreme severity	BanTH
4: তুমি ব্যট অস্কর প্রপ্ত। (You seem like an Oscar-winning man.)	HS	NHS	Target: Individual Form: Behavior-based Content: Sarcasm & Critique Impact: Mock achievement	BanTH

### Data sourcing

We outline the datasets used to enhance our dataset, highlighting their fundamental characteristics. These resources have substantially influenced hate speech detection and interconnected domains characterized by equivalent linguistic patterns.

**BD-SHS** [44] The BD‑SHS dataset comprises 50,281 Bengali comments manually annotated for hate speech. To construct a diverse and standard corpus, the authors surveyed twenty undergraduate students to identify six domains tending to the controversial discussion, such as sports, entertainment, crime, politics, religion, and miscellaneous topics. They crawled public comment sections on Facebook, YouTube, and TikTok using Facepager and used a Jaccard‑index cutoff of 0.8 to eradicate duplicate entries. The annotation process followed a three-level hierarchical strategy to provide both granularity and consistency, entitling researchers to scrutinize not only the presence of hateful language but also its intended targets and thematic characteristics.

**BanTH** [45] The dataset contains 37,350 transliterated Bangla text samples extracted from YouTube comments. These samples were collected utilizing the YouTube API from three primary categories: News & Politics, People & Blogs, and Entertainment. Data collection covers a period from January 2020 to July 2024, thereby capturing discussions on significant events such as the Bangladesh quota reform movement. The dataset is split into two primary subsets: 10,282 samples labeled as hate speech and 27,167 samples labeled as non-hate speech. Furthermore, each hate speech example is annotated with one or more of the following categories: Political, Religious, Gender, Personal Offense, Abusive/Violence, Origin, and Body Shaming.

**BN-Hate** [9] The BanglaHate (BN-Hate) is a balanced Bengali hate speech corpus comprising 15,000 social media posts. The dataset contains an equal number of 7,500 hate and 7,500 non-hate samples. All samples were manually annotated by 27 native Bengali speakers to confirm high-quality labeling. The data were collected from Facebook and YouTube comments over a period spanning January 1, 2021, to April 5, 2022. Notably, the dataset captures eight distinct categories of hate speech, including xenophobia, racism, religious intolerance, and homophobia.

**Bengali Hate Speech Dataset** [36] The Bengali Hate Speech Dataset provides a diverse 30,000 user comments—10,000 hate speech and 20,000 non‑hate–-collected from YouTube and Facebook to reflect authentic social media conversation. The dataset encompasses seven domains (sports, entertainment, religion, politics, crime, celebrity, and TikTok/meme), it captures linguistic diversity through features such as emojis, Bengali–English code‑mixing, including informal spellings. Comments were drawn from contentious discussions to mimic real‑world dynamics. Fifty native annotators each labeled every comment thrice, with majority voting determining the final class. Hate examples comprise 33% of the dataset to represent a class imbalance in natural settings.

**Bengali Multi-Labeled Toxic Comments Dataset** [46] The dataset includes 16,073 manually annotated Bengali social media comments collected from YouTube and Facebook, of which 8,488 are toxic and 7,585 are non‑toxic. Toxic comments are further subdivided into six overlapping types–-vulgar, hate, religious, threat, troll, and insult–-enabling robust multi‑label classification. Each comment was separately labeled by two of three professional annotators, with any disagreements resolved through expert decision; the resulting inter‑annotator agreement score indicates excellent reliability. Overall, this benchmark presents a linguistically diverse and contextually rich resource for advancing Bengali toxic‑speech detection.

**Vio-Lens** [17] This corpus encloses 6,046 Bengali comments sourced from YouTube videos on violent content in Bangladesh and West Bengal, assembled for the BLP‑2023 shared task on Violence Inciting Text Detection (VITD). It is annotated into three classes–-Direct Violence (786 samples), Passive Violence (2,058 samples), and Non‑Violence (3,202 samples)–-indicating a noticeable class imbalance. Annotations were performed by Bangladeshi undergraduates using a strategy grounded in prior literature, with experts fixing any disharmonies through majority voting. Ultimately, this resource emphasizes the challenges of detecting culturally nuanced incitement of violence in a low‑resource language.

**Bangla Cyberbullying Comments** [47] This dataset features 44,001 user comments scraped from publicly accessible Facebook pages of Bangladeshi celebrities, social media influencers, politicians, athletes, and singers. Each comment was manually categorized into one of five harassment categories–-Non‑bully, Sexual, Threat, Troll, and Religious–-to capture nuanced forms of online abuse. Data collection employed automated crawling of Facebook comment sections, followed by strict cleaning to remove noise and any non‑Bangla text. Annotations were then performed and confirmed by native Bengali speakers to assure both linguistic accuracy and cultural relevance.

**Bengali Cyber Bullying Dataset** [48] This kaggle dataset contains 6,010 Bangla‑language social media comments gathered via web scraping from platforms such as YouTube, Facebook, and Twitter. Each example is labeled with exactly one of five cyberbullying categories–-political harassment, sexual harassment, trolling, threats, or neutral content–-thus reflecting the most predominant forms of online abuse in Bangla contexts. To facilitate robust learning, the dataset is deliberately balanced, with roughly 1,202 examples per class.

### Data preprocessing

We extracted the column of Bangla text along with its corresponding label from each corpus. Within the eight datasets, BanTH contains transliterated Bangla text, which repeatedly had inaccurate and semantically divergent back-translations from the original. To address this problem, we utilized Gemini-2.0 Flash [49], the most advanced language model for understanding the Bangla language, especially as an iterative translator and judge through strategic prompting steps (Fig 1). This method facilitated self-critique and refined correction of translation outputs, resulting in remarkably improved standard Bangla translations (Table 2).

**Fig 1:**
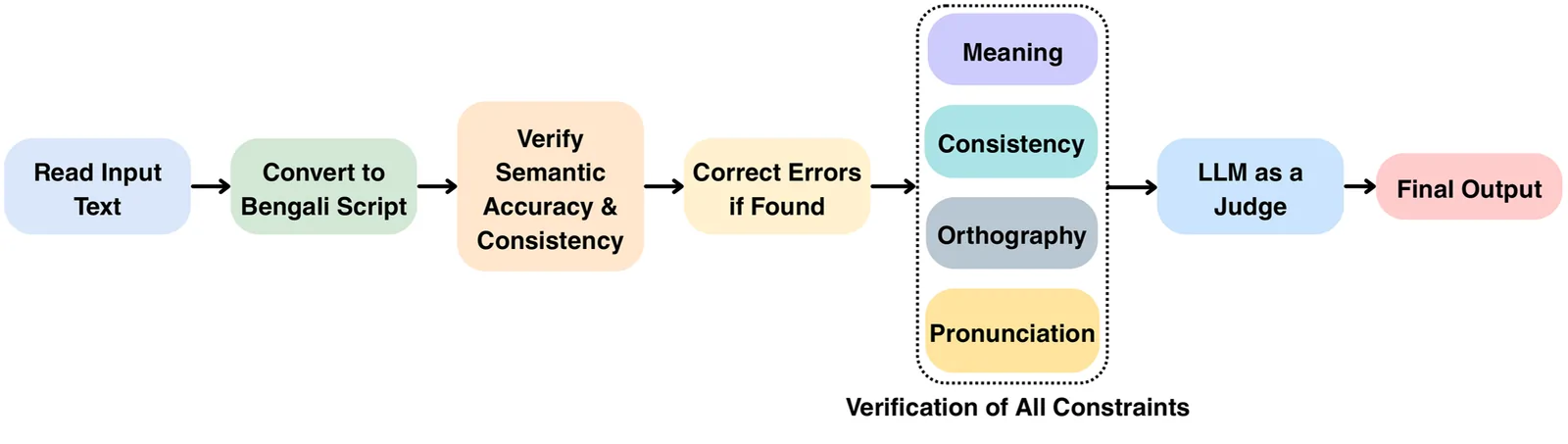
Workflow for the transliteration and verification of Romanized Bangla into standard Bengali script. The pipeline details the sequential steps from input reading to final output, featuring an automated error-correction phase. The process is governed by a four-fold constraint verification system evaluating Meaning, Consistency, Orthography, and Pronunciation, judged by a Large Language Model to ensure semantic and linguistic accuracy.

**Table 2:** Comparison of translation quality between back-translated BanTH datasets examples and our strategically prompted Gemini-based translation approach.

Transliterated Text	Back Translated Bangla	Gemini’s Response
1: Ai beti valo ovinoy jane (This woman is an excellent playwright.)	এই বেশি ভলো অভিনয় জনে। (×) (This knows good drama.)	এই বেটি ভলো অভিনয় জনে। (✓) (This woman is an excellent playwright.)
2: Vua vua (Fake fake!)	ভভ ভভ! (×) (Wow wow!)	ভূয় ভূয়! (✓) (Fake fake!)
3: Chatrolik ke ber korte hobe bangladeshe (Chatroleague must be kicked out from Bangladesh.)	চলক কে বের করতে হবে বংলাদেশে। (×) (A driver needs to be kicked out from Bangladesh.)	ছাত্রলীগকে বের করতে হবে বালাদেশে। (✓) (Chatroleague must be kicked out from Bangladesh.)
4: Lutpat kore akn abar vondamu (After looting, now they’re pretending to be righteous.)	লুতপাত করে এখন আবার ভন্ডামু। (×) (After lutpat, now they’re pretending to be righteous.)	লুটপাট করে এখন আবার ভন্ডামি। (✓) (After looting, now they’re pretending to be righteous.)
5: Amr mata gurche (My head is spinning.)	আমার মাটা পুড়ছে। (×) (Invalid translation in English.)	আমার মাথা ঘুরছে। (✓) (My head is spinning.)

We then concatenated all eight corpora, obtaining 189,761 samples primarily. For text preprocessing, we defined a comprehensive character set comprising 72 frequently occurring Bangla characters denoted as, $DC=\{DC_{1},DC_{2},...,DC_{72}\}$, 16 punctuation marks, $PM=\{PM_{1},PM_{2},...,PM_{16}\}$, and the space character *SP*, resulting $C=\{DC\cup PM\cup SP\}=\{C_{1},C_{2},...,C_{89}\}$. Each sentence $S=\{S_{1},S_{2},...,S_{N}\}$, where *N* is the number of characters, was filtered to include only characters in *C*. By construction, this character-level filter excludes emojis, Latin-script alphanumeric characters, and any other non-Bangla Unicode symbols present in the original scraped text; consequently, the subword vocabulary derived by the Byte-Pair-Encoding tokenizer τ(.) is composed exclusively of standardized Bangla graphemes, punctuation, and whitespace, and is unaffected by emoji usage or alphanumeric substitution artifacts. Furthermore, we defined five types of null values, $N=\{N_{1},N_{2},...,N_{5}\}$, six NaN values, $NV=\{NV_{1},NV_{2},...,NV_{6}\}$, and exact‑match verbatim instances, *EM*, constituting $V=\{N\cup NV\cup EM\}=\{V_{1},V_{2},...,V_{12}\}$, and removed any instance $I_{i}\in D=\{I_{1},...,I_{K}\}$ containing values in *V*. To ensure semantic diversity, we computed the Jaccard similarity [50], $Jaccard(A,B)=\frac{\left|A\cap B\right|}{\left|A\cup B\right|}$, between sentence character sets and eradicated sentences with similarity above 0.80 for length 𝐥≥5 and above 0.75 for 1≤𝐥<5. After cleaning, the combined dataset comprised 168,406 sentences labeled as hate or non‑hate. For our rigorous annotation, we uniformly sampled 1,500 hate‑speech instances across 17 existing subcategories and 1,500 non‑hate instances. In multi‑labeled cases, any hate category was binarized to label 1 and all others to label 0, producing an exhaustive binary classification corpus. This systematic preprocessing ensures a standardized, linguistically coherent dataset for subsequent annotation and modeling.

### Data annotation

Our data annotation process is twofold. First, we carried out a rigorous selection of complete, high‑quality sentences, which were then manually labeled by human annotators. In the second stage, we leveraged state‑of‑the‑art large language models–-Google’s Gemini [49] and Gemma [51]–-as annotators and judges for advancing further annotations, ensuring consistency and appropriateness of each hate-speech label.

#### Human annotation.

We created a balanced subset of a Bengali hate-speech corpus by uniformly sampling 1,500 instances from a total of 17 predefined categories. These categories include Xenophobia, Racism, Sexual Hate, Religious Hate, Homophobia, Personal Insults, Geopolitical Issues, Sports, Entertainment, Crime, Politics, TikTok/Memes, Vulgarity, Threats, Trolling, Insults, and Body Shaming. Further, we included 1,500 non-hate examples to ensure equal distribution across the two classes. We ensure that each example is a complete, meaningful sentence. Five native Bengali speakers, undergraduates in Development Studies (two), Sociology (two), and English (one) at the Bangladesh University of Professionals, participated in iterative annotation. Annotators were deliberately selected as native Bengali speakers to ensure accurate capture of local slang, cultural references, and contextual phrasing. Recognizing hate speech as a deeply socio-cultural phenomenon, the team was intentionally assembled as an interdisciplinary cohort, with each disciplinary background contributing a distinct analytical lens: the Sociology students evaluated societal prejudices, group dynamics, and systemic discrimination; the Development Studies students assessed human rights contexts and the vulnerability of marginalized groups; and the English student applied discourse analysis to detect implicit threats, sarcasm, and dog-whistles, while also ensuring precise comprehension of the English-language definitional frameworks used during annotator training. This composition ensured multidimensional evaluation of context-specific indicators guided by interdisciplinary expertise in socioeconomic theory, cultural dynamics, and linguistic analysis. Annotators were trained using internationally recognized frameworks, including the UNESCO guidelines on countering hate speech [38], the United Nations definition of hate speech and its guidelines for identifying vulnerable groups [39], and the Stanford Encyclopedia of Philosophy entry on hate speech [52,53]. Annotation proceeded iteratively, and annotators were permitted to mark uncertain cases as “confusing.” The resulting negligible number of ambiguous instances were subsequently resolved through granular semantic breakdown, triangulation with supplementary external sources, including UNESCO’s guidelines on school violence and bullying [54] and recognized cyberbullying definitions from UNICEF [55] and the Cyberbullying Research Center [56], alongside targeted web searches and final adjudication by subject matter experts (SMEs). Final labels per example were decided by majority vote, producing strong inter-annotator agreement of κ=0.8163 for hate-speech samples and κ=0.7289 for non-hate samples. This thorough strategy, integrating uniform sampling, interdisciplinary review, standardized guidelines, and majority voting, enhances both the linguistic variousness and labeling trustworthiness of this subset, ensuring high consistency and representativeness, as shown by influential Fleiss’ kappa scores. The whole process is illustrated in Fig 2.

**Fig 2:**
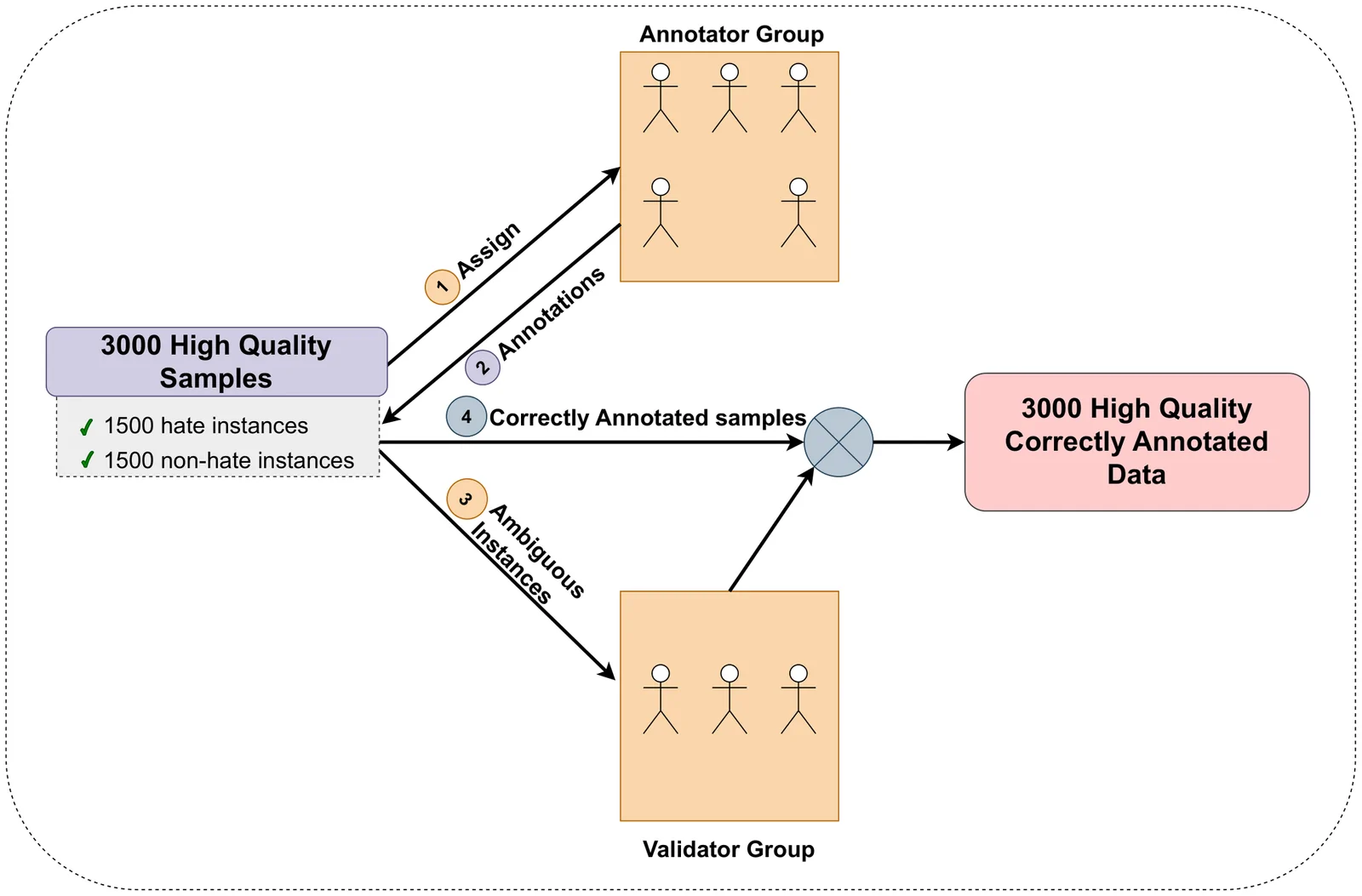
Multi-step human annotation and validation framework for the BanglaHateCorpus. The workflow illustrates the curation of a balanced gold-standard set comprising 1,500 hate and 1,500 non-hate instances. The process involves an initial assignment to the primary Annotator Group, followed by a secondary review of ambiguous instances by a Validator Group to ensure final dataset integrity and high-quality labels.

#### LLM as an annotator and judge.

Manual annotation of above 165,000 Bangla text samples proved prohibitively laborious; therefore, we employed a second-stage pseudo-labeling strategy leveraging trendy large language models (LLMs) to ensure efficient yet accurate labeling.

We first fine-tuned TituLM-Gemma-2 (2B) [57], which is a Gemma-2 (2B) model that Hishab continually pretrained on a large and varied Bangla corpus. This corpus includes web pages, books, transcribed speech, translated content, code-mixed and transliterated text, as well as synthetic data produced using custom LLMs, ASR pipelines, and web scraping. We fed the LLM with 3,000 carefully annotated examples taken uniformly from 17 categories of hate speech to reduce class bias and encompass the entire possible range of hateful content. This initial fine-tuning imparted broad linguistic competence, justifying its usefulness as a pseudo-labeler for the remaining 165,406 instances. Utilizing LLMs to enrich limited or sparsely annotated datasets has been shown to successfully bridge semantic gaps and boost performance in complex retrieval pipelines [27]. Subsequently, we incorporated a proprietary LLM, Gemini, as both annotator and judge, guided by internationally recognized hate-speech standards (Fig 3) through strategic prompt engineering. Gemini produced contextualized labels following stepwise reasoning. These labels were accepted only when they concurred with Gemma’s output; in cases of disagreement, Gemini re-evaluated each sample through a translucent, step-by-step reasoning process, and its final judgment was adopted to maximize label accuracy, as illustrated in Fig 4. Such strict dual-model verification is critical, as relying on automated generation and labeling without structural calibration can introduce synthetic artifacts that deviate from authentic human expressions [30]. By combining uniform sampling, interdisciplinary human supervision, and dual-model unanimity–-with explicit conflict resolution–-we achieved a scalable annotation pipeline that upholds both consistency and contextual nuance, as shown in Fig 5.

**Fig 3:**
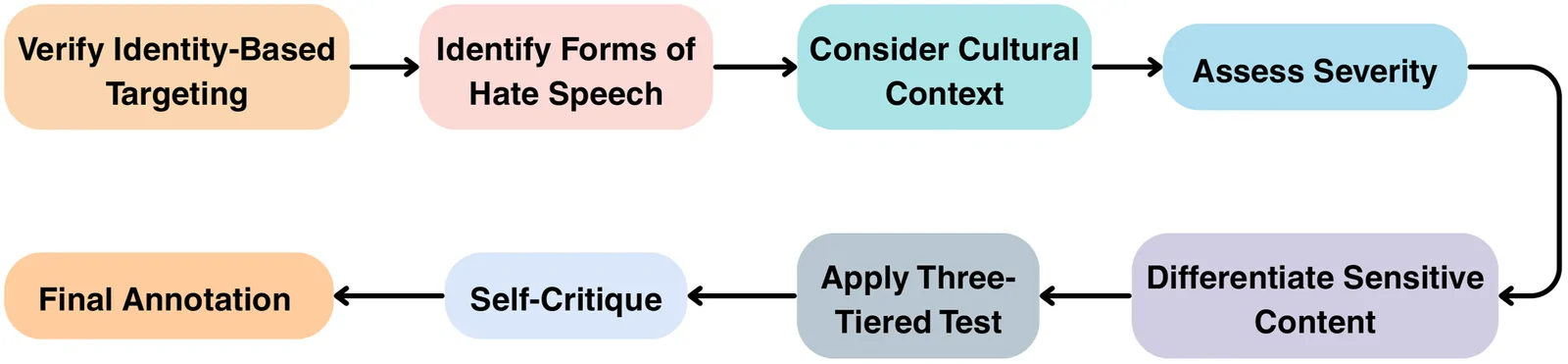
Eight-stage sequential pipeline for LLM-based Bangla hate speech annotation. The diagram outlines the systematic processing flow from initial verification to self-critique and final annotation output.

**Fig 4:**
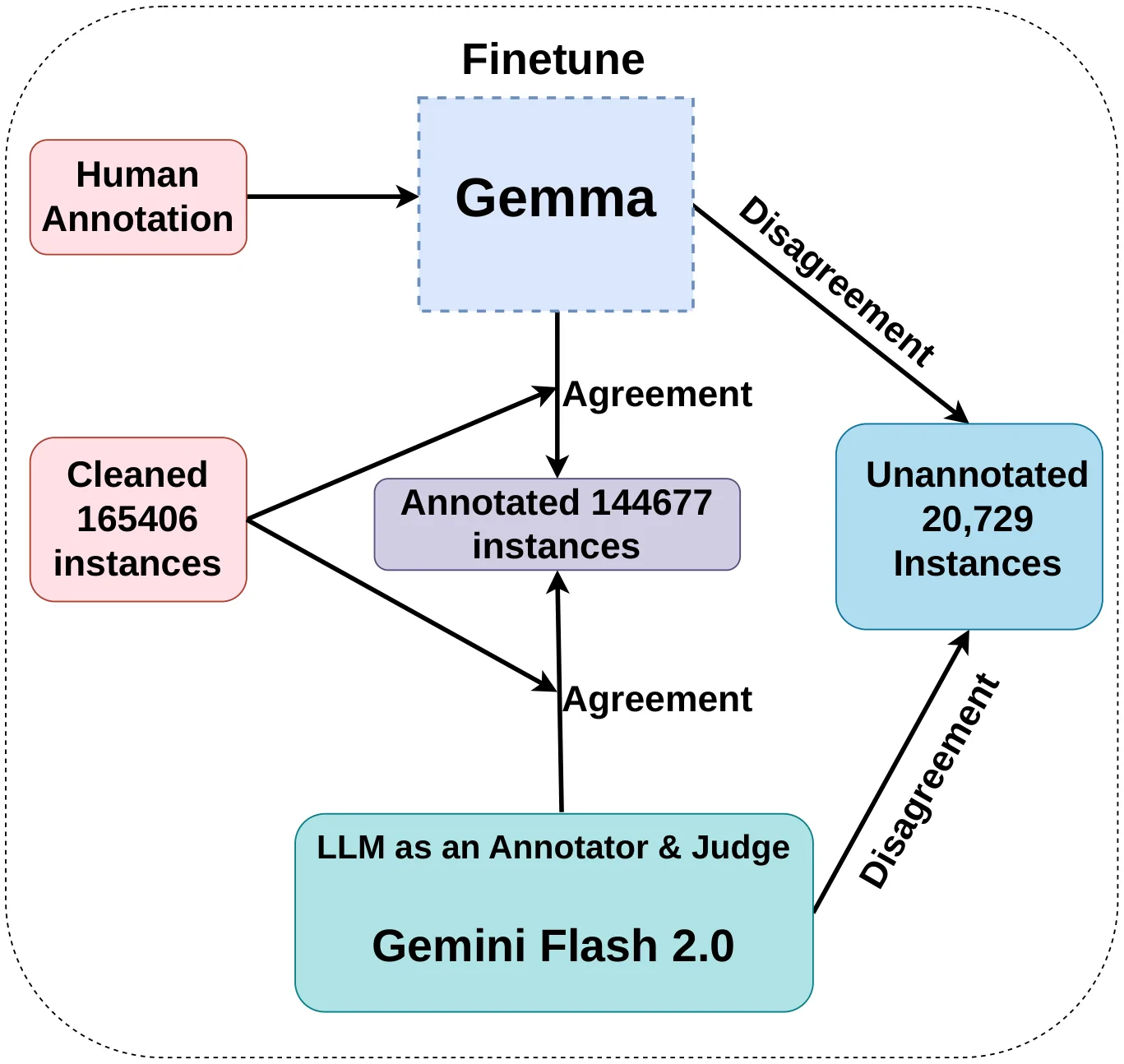
Workflow for harmonizing Large Language Models (LLMs) in data annotation. The diagram illustrates the iterative process involving a fine-tuned Gemma model (supported by human annotation) and Gemini Flash 2.0 acting as an annotator and judge. The process successfully annotated 144,677 instances based on model agreement, while 20,729 instances were flagged as unannotated due to disagreement between the models.

**Fig 5:**
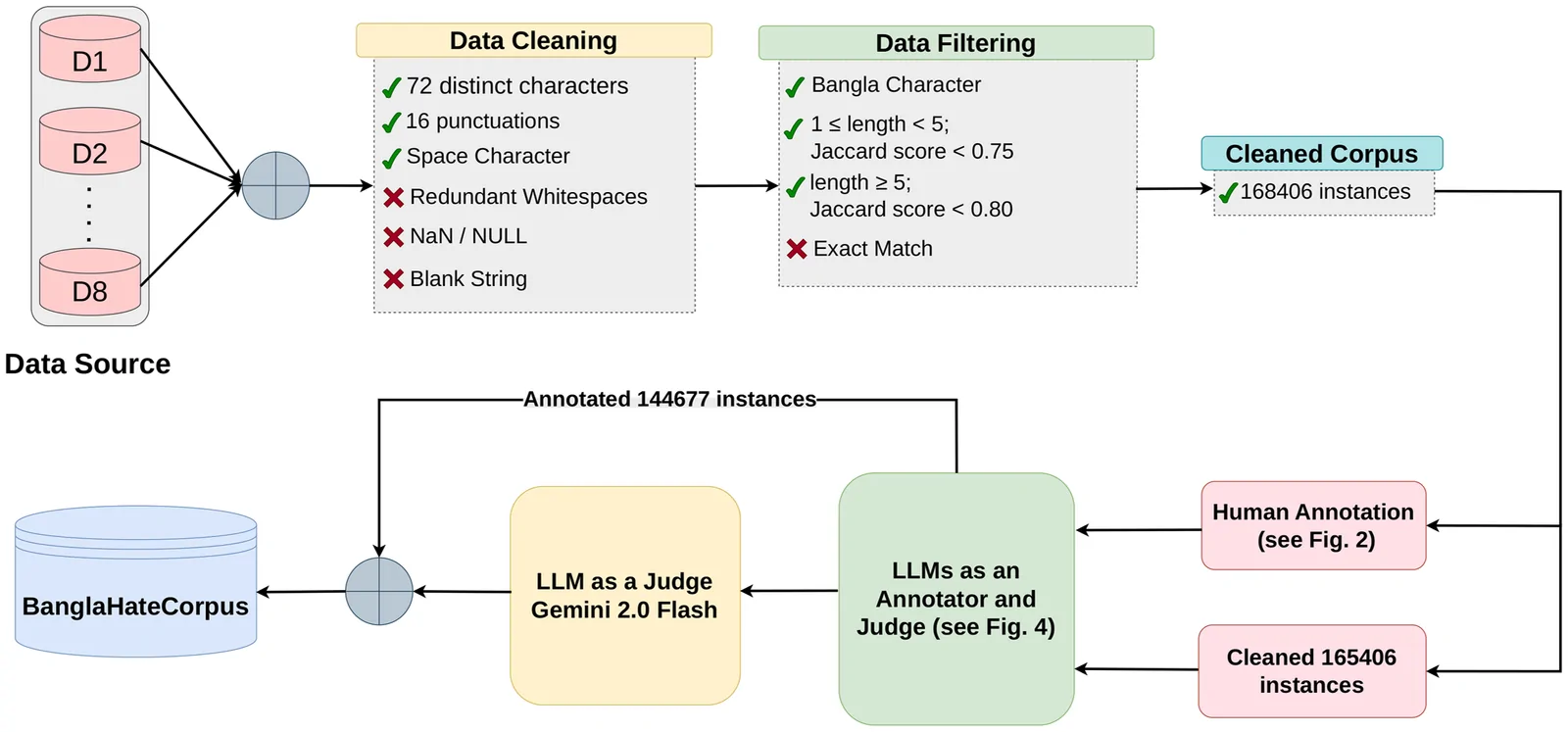
End-to-end pipeline for the BanglaHateCorpus dataset creation. The workflow demonstrates the systematic transition from raw data sources (D1–D8) through rigorous data cleaning and filtering phases utilizing Jaccard similarity scores and character-level heuristics to produce a cleaned corpus of 168,406 instances. The final annotation stage integrates a hybrid approach, combining expert human verification (Fig 2) with a multi-LLM framework (Fig 4) featuring Gemini 2.0 Flash as a judge to curate the final corpus.

Our corpus of 165,406 samples shows noteworthy inter-annotator agreement (Cohen’s Kappa = 0.7402), highlighting the effectiveness of dual-model pseudo-labeling over exhaustive manual effort. Moreover, the near-even class distribution—70,760 positive and 94,646 negative labels from Gemma, alongside 62,579 positive and 102,827 negative from Gemini–-ensures that downstream classifiers will not be excessively biased toward either category, indicating Gemma is somewhat sensitive to hate speech. Additionally, the agreement rate of 87.5% (based on 144,677 instances) exhibits the consistency of the label assignments.

A Detailed confusion-matrix (Fig 6) analysis further reveals that our conflict-resolution mechanism effectively addresses the 12.5% of disagreeing cases through transparent, stepwise re-evaluation by Gemini, thereby heightening overall label fidelity. In addition, lexical coherence, as measured by average Jaccard similarity, is slightly higher within the hate class (0.0153) than the non-hate class (0.0106), and the between-class separation of 0.0047 proves clear lexical dissimilarity. The semantic similarity scores based on BERT further support this tendency. Hate texts have an average BERTScore of 0.6962, while non-hate texts have an average of 0.6794, resulting in a class detachment of 0.0122. Additionally, the approximate BERT metrics [58] for the entire dataset show 0.8743 for hate texts and 0.8676 for non-hate texts, revealing strong intra-class cohesion on a larger scale. In contrast, we conducted a rigorous quality evaluation compared to the datasets listed in Table 3. By harmonizing uniform selection, rigorous human oversight on the subset, dual-LLM consensus, and encyclopedic coherence evaluation, we introduce BanglaHateCorpus: a scalable, high-quality dataset that fully captures the breadth of Bangla hate speech and is finely adjusted for reliable model training. This multi-tiered framework maximizes label adherence and representativeness, illustrating a tangible foundation for downstream detection tasks.

**Fig 6:**
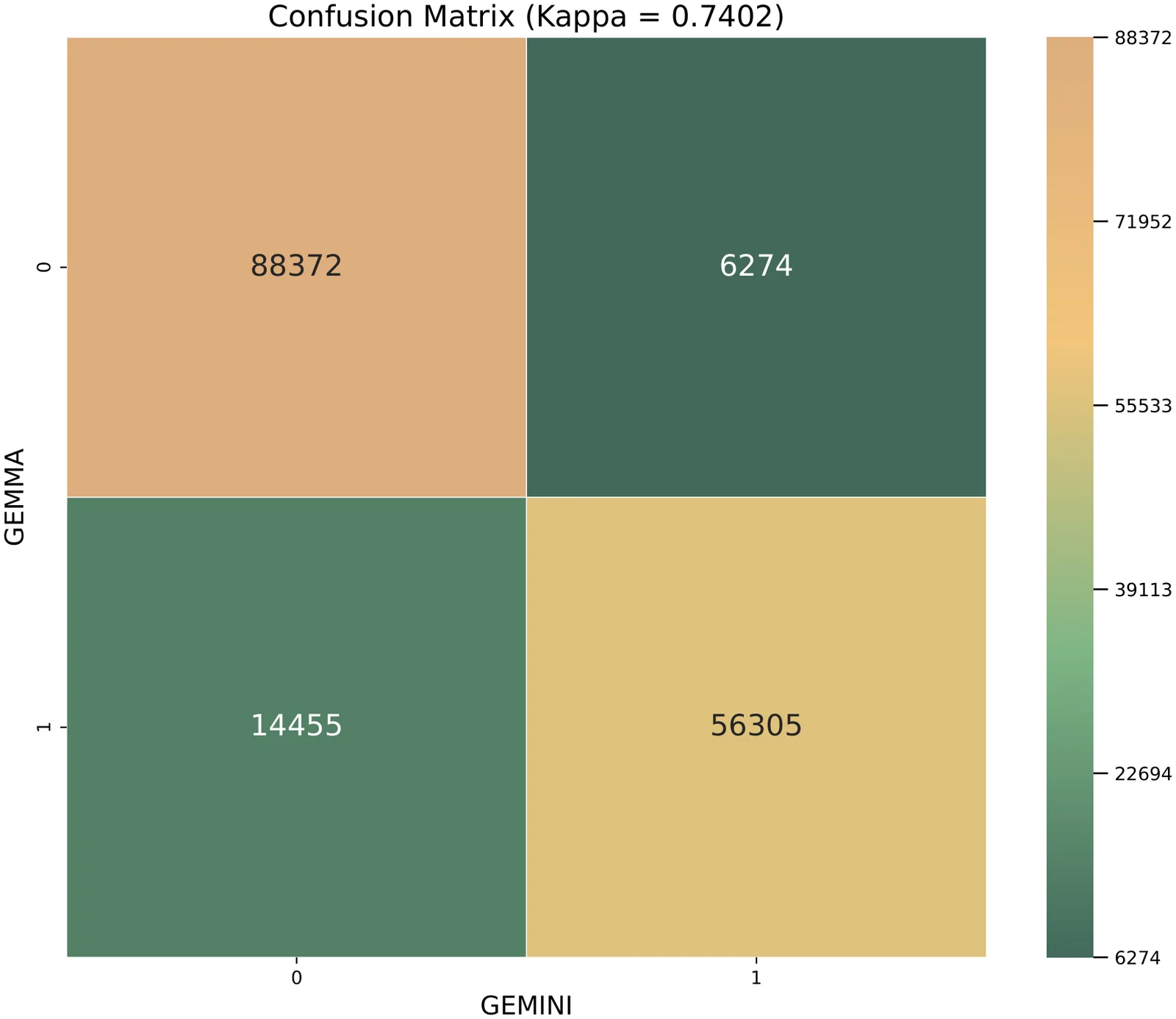
Confusion matrix illustrating inter-model agreement between Gemma and Gemini pseudo-labels. The matrix evaluates the classification alignment across 165,406 samples, yielding a Cohen’s Kappa coefficient of 0.7402. This score indicates a substantial level of agreement between the two Large Language Models in identifying hate speech instances, providing a robust basis for the subsequent harmonization and final dataset curation.

**Table 3:** Comparison of class‑separation metrics across standard benchmark datasets (LSR = Lexical Separation Score; SSR = Semantic Separation Score).

Dataset	Jaccard Sep.	BERT Sep.	LSR	SSR
BanTH	0.0016	0.0009	0.080	0.022
BD‑SHS	0.0056	0.0045	0.233	0.023
BanglaHateCorpus	0.0047	0.0168	0.307	0.024

### Corpus statistics

Our proposed BanglaHateCorpus corpus comprises 168,406 instances, with 63,779 examples of hate speech (37.9%) and 104,627 examples of non-hate speech (62.1%).

The dataset was created through a twofold meticulous annotation process that leverages both human expertise and LLMs’ language understanding capabilities. We adopted binary annotation as a sensible solution to address the urgent scarcity of large-scale, meticulously annotated hate speech datasets in low-resource language settings such as Bangla. Our approach follows international standard practices where fine-grained labeling is hindered by involving limited linguistic expertise, high annotation costs, and the subjective complexity of representing nuanced hate speech categories. In our corpus, text length deviates between 1 and 3,427 words overall; non‑hate samples range from 2 to 3,427 tokens, and hate samples from 1 to 2,402 tokens. On average, sentences contain 12.50 words, with non‑hate texts averaging 11.02 words and hate texts averaging 14.92 words. Over 80% of instances, depicted in Fig 7, hold more than five words, yet fewer than 20% surpass twenty words, exhibiting predominantly short to medium‑length texts. Notably, hate speech samples are lengthier: 23.5% contain at least twenty words compared with 11.6% of non‑hate samples. We partitioned the corpus into three subsets: 156,406 instances for training (92.9%), 6,000 for validation (3.56%), and 6,000 for testing (3.56%). This split ensures computational efficiency while preserving enough samples to capture the semantic nuances of hate speech and to support reliable benchmarking across various detection models. Fig 8 and 9 quantify each word’s proximate contribution to its class. For hate speech, influence is calculated as (word coefficient/sum of positive coefficients) × 100; formally:

**Fig 7:**
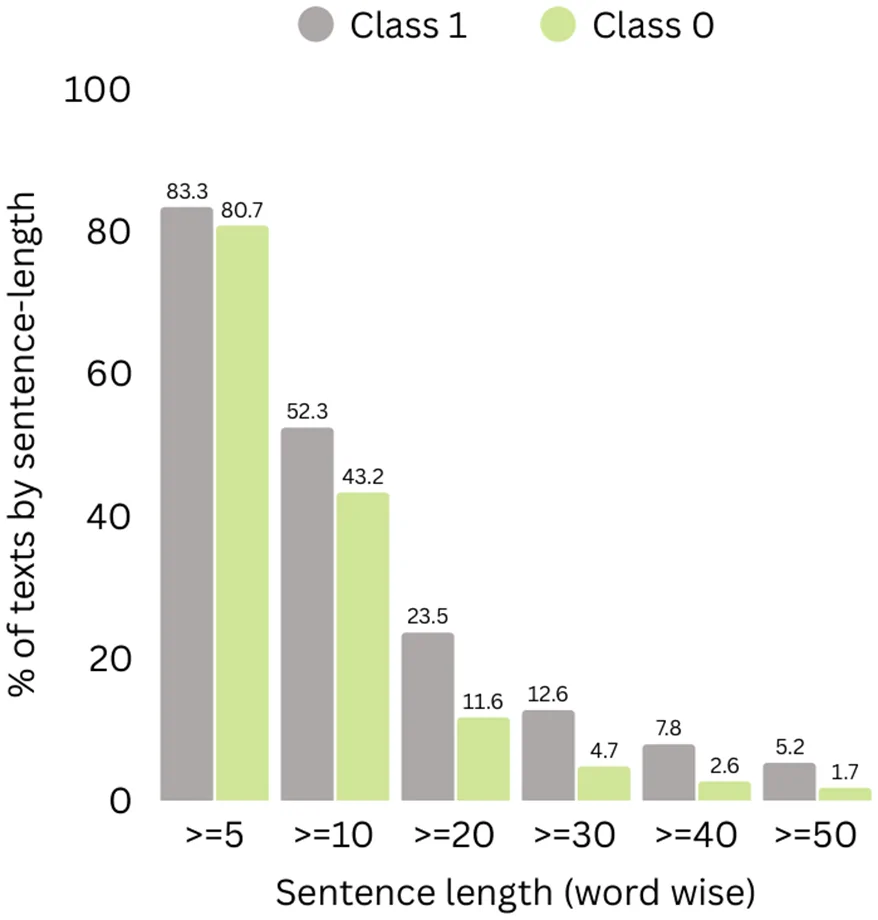
Distribution of text samples categorized by word-wise sentence length across classes. The bar chart illustrates the percentage of texts within specific length intervals for Class 1 (Hate) and Class 0 (Non-hate). A clear downward trend is observed as sentence length increases, with the majority of samples concentrated in the ≥5 and ≥10 word categories, providing insights into the typical linguistic structure of the annotated social media comments.

**Fig 8:**
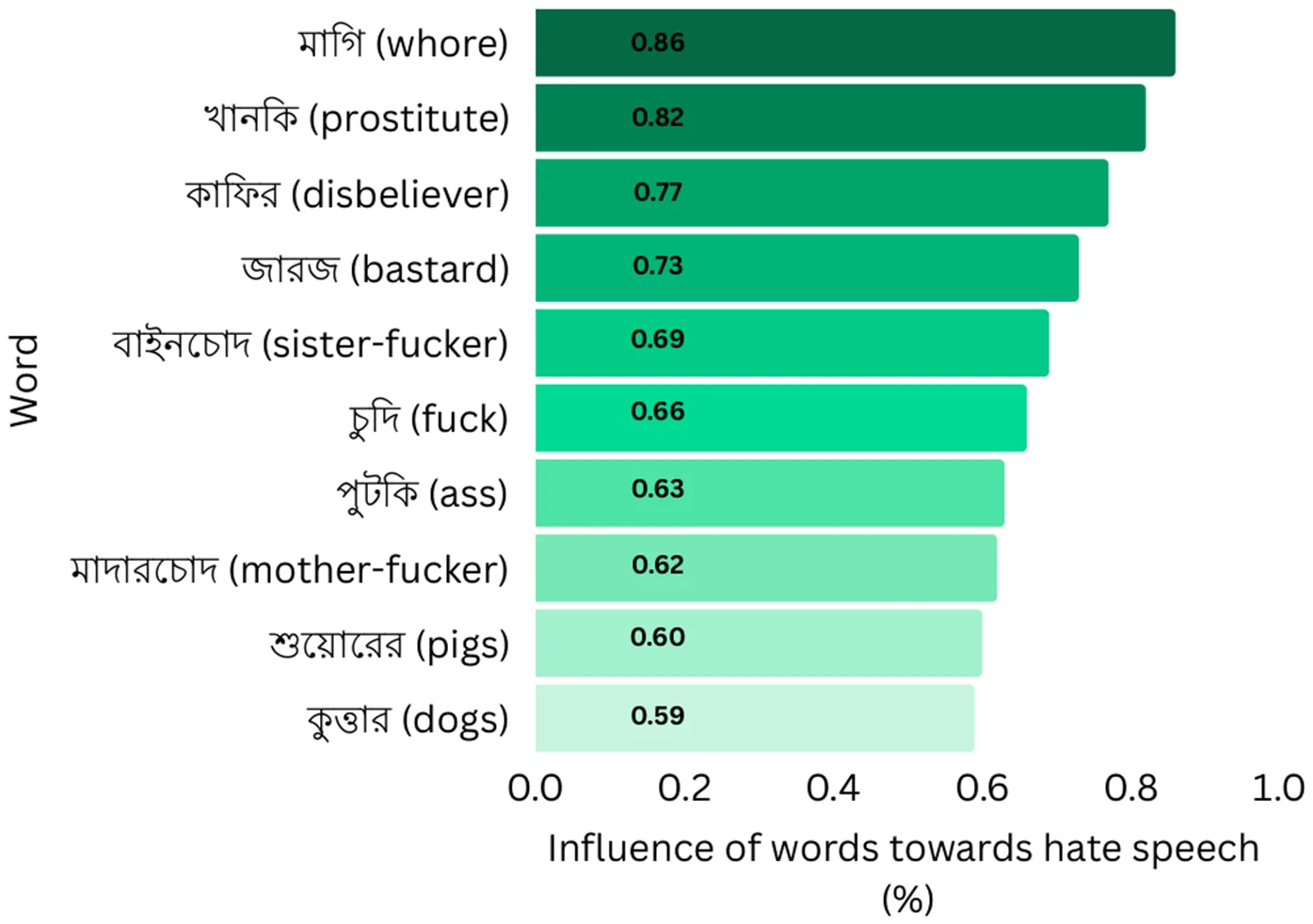
Influence of individual words on the classification of hate speech. The bar chart illustrates ten words with the highest impact scores, indicating their strong association with hateful content. Each score reflects how much each word contributes to the model’s prediction of hate speech.

**Fig 9:**
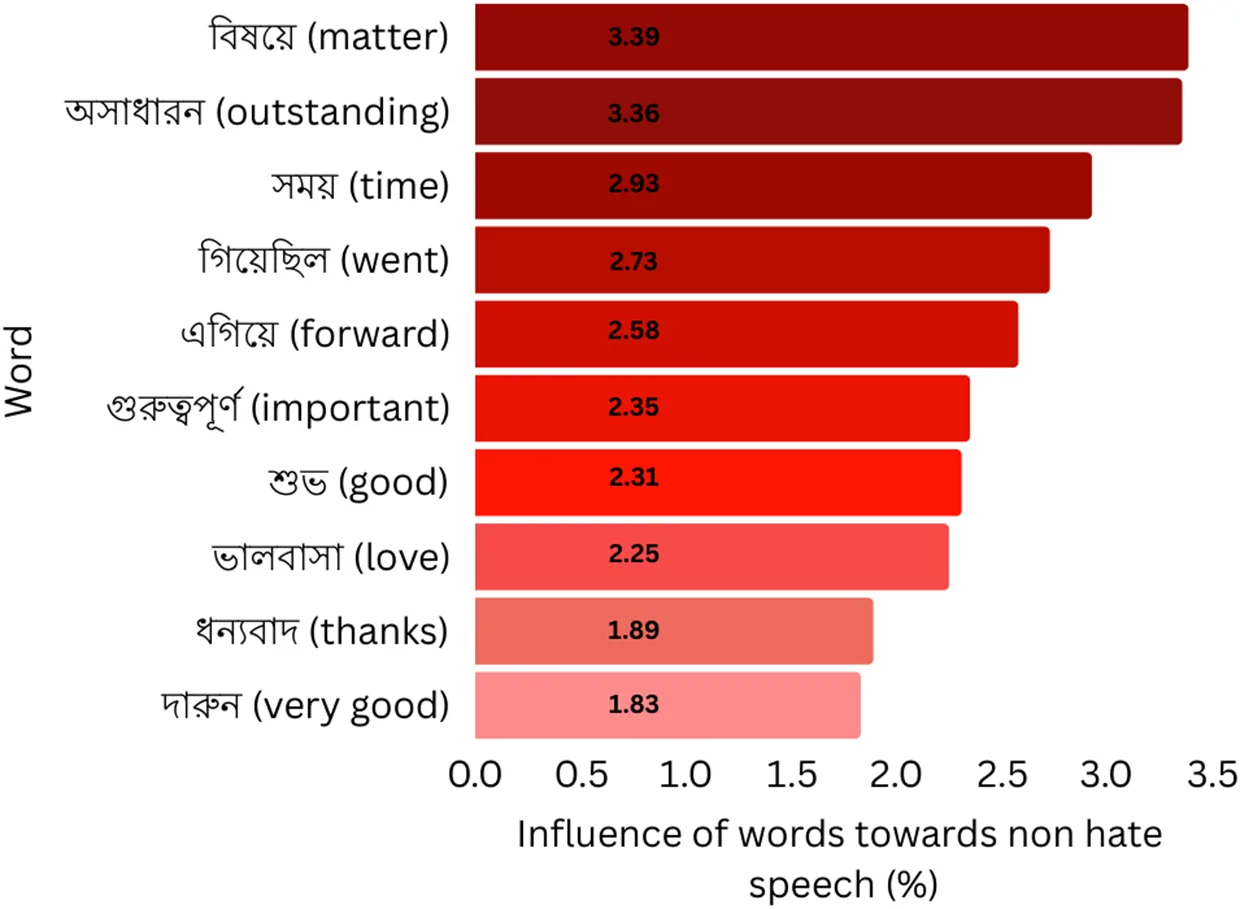
The bar chart spotlights the top ten words that most strongly signal non‑hate speech. Each word’s influence score quantifies its contribution to the model’s prediction that a given instance is non‑hateful.


$$\begin{array}{c} \text{Influence (\%)}=\frac{w_{i}}{\sum_{j\;:\;w_{j}>0}w_{j}}\times 100 \end{array}$$ (1)

where $w_{i}$ denotes the logistic regression coefficient of word *i*. Conversely, for non-hate speech:


$$\begin{array}{c} \text{Influence (\%)}=\frac{\left|w_{i}\right|}{\sum_{j\;:\;w_{j}<0}|w_{j}|}\times 100 \end{array}$$ (2)

This normalization converts raw model coefficients into interpretable relative contribution shares, ensuring that each word’s influence is expressed as a percentage of the total discriminative signal directed toward its respective class [59]. Words with higher influence percentages exert proportionally greater weight on the model’s classification decision, enabling transparent, model-faithful attribution. Our analysis reveals distinguishable vocabulary separation between hate and non-hate content, ensuring that our LLM-assisted annotation process effectively captured meaningful linguistic patterns. This lexical distinctiveness validates both the annotation process and the overall robustness of the BanglaHateCorpus dataset.

## Proposed method

We propose a novel deep learning architecture that leverages optimized transformer components to distinguish between hateful and non-hateful speech accurately. The initial step begins by processing the input text sequence, $\left[x_{1},x_{2},...,x_{n}\right]$, through a pre-trained Bangla tokenizer, τ(.), which encodes the textual input into the numerical format. This transformed input is subsequently fed into the model, μ(.) to generate a classification output. The prediction, $\hat{y}$, is then validated utilizing evaluation metrics tailored to the classification task. The complete Bangla hate-speech classification framework is depicted in Fig 10. Mathematically, the entire process can be concisely summarized as shown in Eq 3.


$$\begin{array}{c} \hat{y}=\mu (\omega (\tau ([x_{1},x_{2},...,x_{n}]))) \end{array}$$ (3)

**Fig 10:**
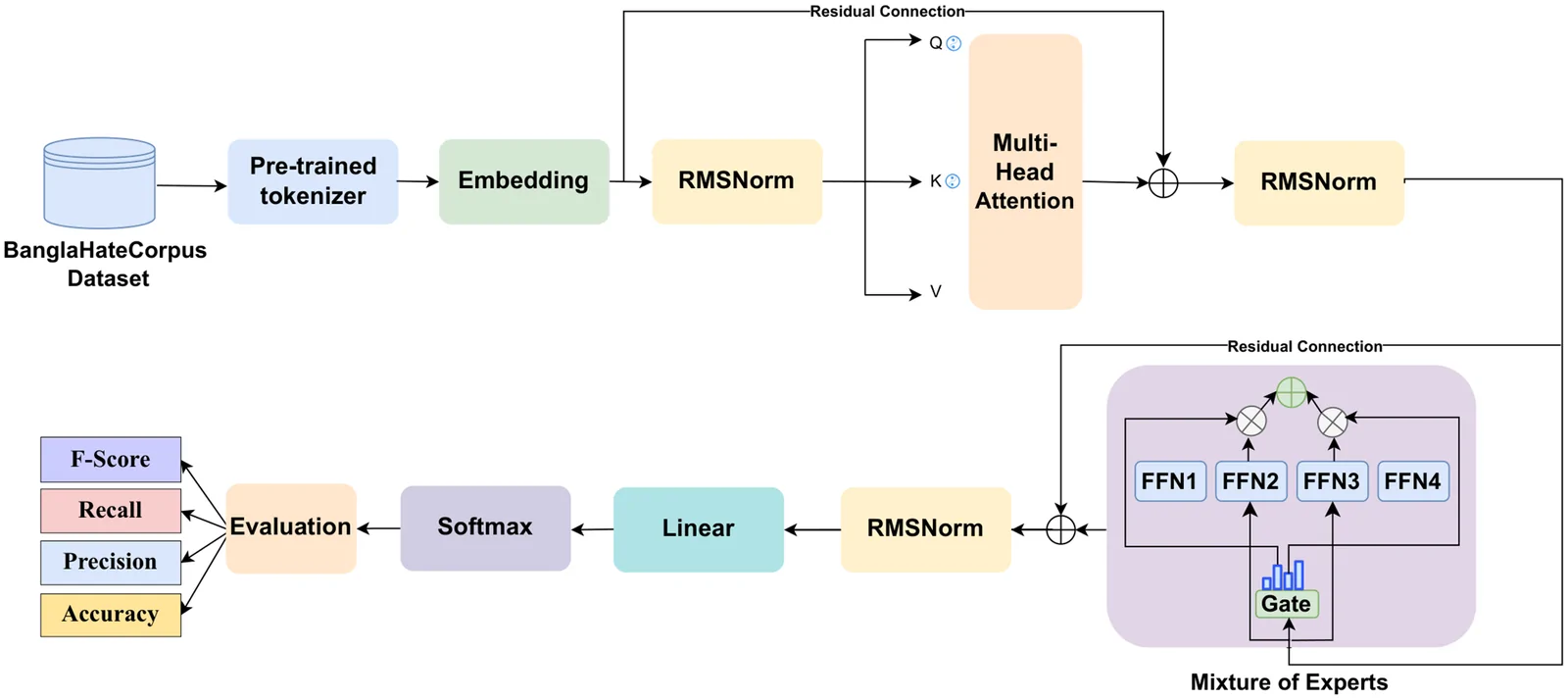
Architecture of the BanHateFormer. Bangla text is tokenized and embedded, then sequentially processed through RMSNorm-normalized multi-head attention blocks with residual connections, gated feed-forward layers (FFN1–FFN4), and a mixture-of-experts module before the final linear-softmax output. Evaluation pipeline measuring model performance via accuracy, precision, recall, and F1-score. This figure contrasts the structural components of our proposed model with its corresponding assessment workflow.

### Problem formulation & overview

The hate-speech classification task strives to map an instance sequence denoted as $X=[x_{1},x_{2},...,x_{n}]$ into the corresponding true label denoted as $Y=[y_{1},y_{2},...,y_{m}]$ where $X_{i}$ and $Y_{j}$ are the *i*th instance and *j*th label respectively, such that the length of the sentence $n\in {\mathbb{Z}}^{+}$ and corresponding label m∈[0,1]. Initially, the sentence $X_{I}$ is tokenized using a pre-trained Byte‑Pair‑Encoding [60] tokenizer τ(·). This process produces a subword-level tokenized representation of *X*, denoted as $X_{\tau }=\{x_{\tau_{1}},x_{\tau_{2}},...,x_{\tau_{n-1}},x_{\tau_{n}}\}$, where $x_{\tau_{i}}$ corresponds to the numerical value of the *i*-th subword token if the subword is present in the vocabulary. If the subword is absent, an ⟨unk⟩ token is used instead. Afterward, the Bangla hate-speech classification model denoted as μ(.), processes the tokenized sentence $X_{\tau }$ and generates a predicted label denoted as $\hat{y}$. Finally, the model’s prediction is appraised through multiple evaluation metrics by comparing $\hat{y}$ with the respective target.

### BanHateFormer

BanHateFormer is a tweaked version of the LLaMA [61] style transformer architecture, specifically designed for Bangla hate-speech classification. The preference for a transformer backbone is inspired by the exceptional success of recent transformer-based models, including BERT [62], LLaMA [61], GEMMA [51], and Mixtral [63], which have achieved state-of-the-art performance on both English and Bangla hate-speech classification benchmarks. Moreover, BERT’s ubiquitous performance in diverse text classification tasks accentuates the efficacy of self-attention mechanisms for nuanced language understanding. Recently, the resurgence of the Mixture-of-Experts (MoE) [64] concept has shown significant improvements in capacity and computational efficiency in modern LLMs. BanHateFormer replaces LLaMA’s standard feed‑forward block with a sparse MoE network, integrates rotary positional embeddings and multi‑head self‑attention, and concludes with a binary classification head. By harmonizing these components, BanHateFormer not only captures fine-grained positional details but also dynamically allocates expert capacity to the most salient token representations. Below, we provide a comprehensive description of BanHateFormer, detailing its architectural innovations.

We transform each sentence into a sequence of tokens $X=\{x_{1},x_{2},...,x_{n}\}$ and assign discrete values to each subword. Here, *n* is the sequence length. To ensure consistent input dimensions for every input sequence, we incorporate padding into *X*. Subsequently, each token $x_{i}$ is passed through an embedding layer *E*, which transforms the discrete values into continuous vectors represented by a trainable matrix $E_{x_{i}}=Embedding(x_{i})$. Through backpropagation during training, these embeddings are fine-tuned to minimize the loss. To enhance gradient flow and accelerate generalization, we pre-normalize the embedding using RMSNorm [65] before preserving the positional order of tokens. To encode relative positions, we incorporate rotary positional encoding *RoPE* [66] for each token $x_{i}$, denoted $RoPE_{x_{i}}$. In RoPE, an embedding vector ${𝐱}_{i}\in {\mathbb{R}}^{d}$ is split into two halves, ${𝐱}_{i}^{\left(1\right)}$ and ${𝐱}_{i}^{\left(2\right)}$, which are converted by a position‐dependent rotation matrix $R_{i}=[\begin{array}{cc} \cos(\theta_{i}) & -\sin(\theta_{i})\\ \sin(\theta_{i}) & \cos(\theta_{i}) \end{array}]$, where $\theta_{i}=\frac{i}{10000^{2k/d}}$ encodes the token’s position. This rotation directly embeds relative positional details, facilitating the dot‐product attention mechanism to reminisce token distances |i−j| through the rotated query ${\tilde{𝐪}}_{i}$ and key ${\tilde{𝐤}}_{j}$ vectors. RoPE generalizes across varying sequence lengths while remaining parameter‐free and computationally efficient, by focusing on relative positions instead of absolute ones. Finally, we add this positional encoding to the normalized embedding, resulting in a composite embedding $Z_{x_{i}}=E_{x_{i}}+{RoPE}_{x_{i}}$. The composite embedding is then fed into a MoE network consisting of *K* experts and a multi-head self-attention mechanism. The self-attention mechanism is the key segment of the transformer architecture. It computes attention scores between all pairs of tokens in the input sequence *X* using three learnable matrices: query (*Q*), key (*K*), and value (*V*). These matrices capture the contextual dependencies between tokens in the sequence. Specifically, the self-attention mechanism calculates a weighted sum of the hidden states for each token, where the weights are based on the similarity between the token and all other tokens in the sequence. This allows the model to focus on the most relevant parts of the input sequence for each token, taking into account the context in which it appears. The mathematical expression of self-attention is defined as follows [67]:


$$\begin{array}{c} \text{Attention}(Q,K,V)=\text{softmax}\left(\frac{QK^{⊤}}{\sqrt{d_{k}}}\right)V \end{array}$$ (4)

Subsequently, the dimension of the keys denoted as $d_{k}$, is used to scale the resultant scores for standardizing overall variance and stabilizing gradients. To enhance the model’s ability to capture multiple relationships and increase its expressive power, the transformer employs multi-head self-attention. Each head independently computes self-attention, allowing the model to focus on different parts of the input sequence simultaneously. The outputs of all heads are concatenated and linearly transformed to produce the final output. The self-attention for each head is calculated as:


$$\begin{array}{c} {\text{Head}}_{i}=\text{Attention}(Q_{i},K_{i},V_{i}) \end{array}$$ (5)

where $Q_{i}=ZW_{i}^{Q}$, $K_{i}=ZW_{i}^{K}$, and $V_{i}=ZW_{i}^{V}$ represent the query, key, and value projections for the *i*-th head. The final multi-head self-attention output is calculated as:


$$\begin{array}{c} MultiHead(Q,K,V)=Concat(Head_{1},Head_{2},...,Head_{h})W^{O} \end{array}$$ (6)

Here, $W^{O}$ is a learnable weight matrix that merges the outputs of all heads. Following the multi-head self-attention mechanism, the output is passed through a Mixture-of-Experts (MoE) layer to enhance the capability for capturing complex token relationships. Each MoE layer comprises *N* parallel experts, with a trainable router–-a linear projection layer–-yielding logits to allocate tokens to experts. Specifically, the router produces per-token logits $z_{j}$, which are softmax-normalized to produce expert selection probabilities. To stabilize training, two auxiliary losses are incorporated: a router z-loss $\lambda_{z}\cdot 𝔼\left[{\left(\log\sum_{j=1}^{N}e^{z_{j}}\right)}^{2}\right]$ [68], which punishes large logit magnitudes, and a load balancing loss $\lambda_{\text{bal}}\sum_{j=1}^{N}{\left(\rho_{j}-\frac{1}{N}\right)}^{2}$ [69], enabling equitable token distribution across experts by minimizing divergences from consistent routing probabilities $\rho_{j}$. During forward passes, only the top-*K* experts per token are triggered, with outputs aggregated via weighted summation $\sum_{i=1}^{K}w_{i}\cdot {\text{Expert}}_{i}(x)$, where $w_{i}$ denotes regularized routing weights. To mitigate overfitting to precise experts, we utilize stochastic expert masking during training with dropout likelihood *p*_drop_. This dynamic computation allocation enables task-specific sub-network activation, enhancing parameter efficiency and scalability compared to dense FFNs. Internal experts employ SwiGLU [70], a parameter-efficient module with gated Swish linear unit (SwiGLU) activations, to introduce non-linearity and distill input representations. The MoE layer incorporates contextualized outputs from multi-head self-attention and expert-derived non-linear transformations, capturing both local and global dependencies that are critical for understanding the meaning and context of each token within the sentence. Residual connections throughout the network preserve information flow and tackle the vanishing gradient problem. The final MoE output is passed to a classification head for label prediction, ensuring semantic and contextual fidelity in encoding input sequences *X*.

### Ethical framework and protocol

**Data Sources and Ethical Compliance** In accordance with general institutional ethical guidelines and the nature of public domain data, the requirement for informed consent was waived as the research involved no direct interaction with human participants. To safeguard privacy, all data were fully anonymized during the preprocessing stage; all Personal Identifiable Information (PII), including usernames and profile links, was removed in compliance with the respective platforms’ Terms of Service.

**Participant Consent Process and Ethical Protections** Informed verbal consent was systematically obtained from a group of undergraduate research assistants (two Sociology students, two Development Studies students, and one English student) who served as annotators for this study. Before the labeling task began, the research team conducted a comprehensive orientation and training session. During this session, the students were informed of the specific research goals, data-handling protocols, and the high likelihood of encountering toxic or offensive content. Following this explanation, each student was allowed to ask questions before being asked for their explicit verbal agreement to proceed. This agreement was formally witnessed and documented in our internal project logs.

The decision to utilize verbal rather than written consent was based on several factors relevant to the academic and social context of the study. As the annotators were senior undergraduate students working under the direct mentorship of faculty, the research was conducted within a framework of existing academic trust and professional development. In this specific institutional environment, formal written contracts are often perceived as overly bureaucratic for internal collaborative research tasks, whereas verbal agreements encourage a more open and communicative relationship between students and supervisors. Furthermore, verbal consent served to protect the students’ privacy by minimizing the collection of personal identifiers that could be linked back to the sensitive dataset.

Significant emphasis was placed on the psychological well-being of the students throughout the process. The verbal consent specifically addressed the “Content Warning” regarding the hate speech corpus, ensuring that participants were mentally prepared for the nature of the text. All annotators were reminded that their participation remained entirely voluntary and that they maintained the right to withdraw from the project at any stage. It was explicitly stated that withdrawal would have no impact on their grades, academic standing, or relationship with the department. This approach ensured that the ethical standards for human subject research were strictly upheld while maintaining a practical and supportive environment for the student researchers.

## Experimental analysis

### BanglaHateCorpus

We developed BanglaHateCorpus, a large‑scale Bangla hate‑speech corpus containing 168,406 text instances. It amalgamates two primary categories, hate and non‑hate speech, encompassing 17 fine‑grained hate subtypes as defined in our annotation guidelines. To maintain optimal model performance and avoid unneeded computational complexity, we restricted the maximum sentence length to 300 tokens since only 0.01% of the corpus overreaches this threshold, and including longer sequences produced no notable improvements. The distribution of sentence lengths (in words) spans from 1 up to 3,427, having an average of 12.50 words per instance; over 80% of samples contain fewer than 20 words. We employ this dataset for binary hate‑speech classification by partitioning it into training, validation, and test sets, ensuring that each subset preserves the overall class balance and linguistic diversity:

- **Training Set:** The training set consists of 156,406 (≈156.4 K) instances, which accounts for 92.9% of the total dataset. This subset is used to train our model; through exposure to these examples, the model learns to differentiate nuanced patterns of hateful versus non‑hateful language.
- **Validation Set:** A held‑out subset of 6,000 samples (≈3.56% of the corpus) used solely for tuning hyperparameters and quest for the optimal model performance during training, confirming we guard against overfitting.
- **Test Set:** Also containing 6,000 samples (≈3.56%), this partition remains intact until final evaluation, delivering a truly unbiased estimation of how competently the model generalizes to new, unseen examples.

### Baselines

- **TB-mBERT** [45]. It is a pre-trained language model designed by fine-tuning the mBERT [62] architecture, particularly to classify transliterated Bangla hate speech. The model was further pre-trained on a large corpus of transliterated Bangla text (BanglaTLit-PT [71]) to enhance its effectiveness in addressing noisy, informal, and script-mixed inputs prevailing in digital Bangla communication.
- **BanglaHateBERT** [9]. This model extends the BanglaBERT architecture [72] through domain-specific adaptation aimed at abusive and hate speech detection in Bengali. By leveraging a diverse collection of 1.5 million offensive Bengali posts, including translated content banned from Reddit, it is optimized to detect nuanced and aggressive language patterns in social media conversations.
- **BanglaBERT** [16]. This is a transformer-based language model pre-trained on a large-scale Bengali text corpus sourced from Wikipedia, news portals, and online forums. Built upon the BERT framework [62], BanglaBERT is designed for general-purpose Bengali language processing and offers strong contextual embeddings for various downstream NLP tasks.

### Performance evaluation

#### Accuracy, precision, recall & macro F1-score.

To assess the effectiveness of BanHateFormer, accuracy and F1 score are two frequently utilized metrics. Accuracy is the frequency of correct predictions made by the model by dividing the number of correct predictions by the total number of predictions. Accuracy can be mathematically abbreviated as follows 7:


$$\begin{array}{c} \text{Accuracy}=\frac{TP+TN}{TP+FN+FP+TN} \end{array}$$ (7)

where TP represents the number of true positives, TN represents the total number of true negatives, FN represents the total number of false negatives, and FP represents the total number of false positives. Accuracy is a useful metric, but the results can be misleading when the ratio of instances in each class is highly imbalanced. Precision is a measure of the proportion of true positive predictions among all positive predictions, indicating how well the model performs at predicting positive outcomes. In contrast, recall measures how many of the positive instances are correctly predicted, demonstrating the model’s ability to capture all relevant instances of the positive class. Precision and recall can be calculated as follows 8, 9:


$$\begin{array}{c} \text{Precision}=\frac{\sum_{i}TP_{i}}{\sum_{i}TP_{i}+\sum_{i}FP_{i}} \end{array}$$ (8)

$$\begin{array}{c} \text{Recall}=\frac{\sum_{i}TP_{i}}{\sum_{i}TP_{i}+\sum_{i}FN_{i}} \end{array}$$ (9)

To ensure equal treatment of both classes, the Macro F1-score 10 is also considered. It is computed by averaging the F1 scores of each class independently:


$$\begin{array}{c} \text{Macro F1}=\frac{1}{N}\sum_{i=1}^{N}\left(\frac{2\times {\text{Precision}}_{i}\times {\text{Recall}}_{i}}{{\text{Precision}}_{i}+{\text{Recall}}_{i}}\right) \end{array}$$ (10)

Here, *N* is the number of classes, and ${\text{Precision}}_{i}$, ${\text{Recall}}_{i}$ represent the precision and recall for class *i*. Macro F1 is especially relevant in scenarios where both false positives and false negatives carry significant consequences. Moreover, we also consider Sensitivity 11 and Specificity 12 to evaluate further classification performance, particularly how well the model differentiates between the two classes. Sensitivity measures the proportion of actual positives accurately identified:


$$\begin{array}{c} \text{Sensitivity}=\frac{\text{TP}}{\text{TP}+\text{FN}} \end{array}$$ (11)

Specificity (true negative rate) measures the proportion of actual negatives correctly specified:


$$\begin{array}{c} \text{Specificity}=\frac{\text{TN}}{\text{TN}+\text{FP}} \end{array}$$ (12)

These metrics provide crucial insights into model performance. Together they deliver a complete understanding of model performance, especially under class imbalance conditions.

### Hyperparameters

The hidden dimension is kept as 512 through all the layers leading up to the final prediction layer for maintaining consistency over the network. To maintain the model’s depth and capacity the number of neurons is kept at 2048 for each expert and a 0.3 dropout ratio is applied to prevent overfitting and the router sends each token to its two highest-scoring experts. To maintain the efficient computation and non-linearity over the network we have incorporated SwiGLU. The model went through 20 epochs with $5\times 10^{-5}$ learning rate incorporating AdamW optimizer. We incorporated the binary cross-entropy loss function for the optimization process, which leads the model towards desired predictions.

### Quantitative analysis

We scrutinize the performance of BanHateFormer, which incorporates a meticulous multi-stage data annotation process across eight corpora. The quantitative results of different hate speech classification baselines are illustrated in Table 4, which explicitly demonstrates the prominence of our proposed BanHateFormer over several transformer-based pre-trained models. BanHateFormer achieves the highest accuracy 91.97%, precision 91.18%, recall 91.50%, macro-F1 91.33%, alongside sensitivity 89.82% and specificity 93.19%, all with only 31.68M parameters. In contrast, BanglaBERT acquires 90.23% accuracy, 90.06% precision, 88.60% recall, 89.24% macro-F1, the sensitivity of 82.68%, and specificity of 94.52%, while XLM-RoBERTa-Base reaches 89.40% accuracy, 88.50% precision, 88.57% recall, 88.54% macro-F1, sensitivity of 85.58%, and specificity of 91.56%. Even the very large Gemma 2-2B(2B parameters) demonstrates 92.17% accuracy, 91.69% precision, 91.28% recall, 91.48% macro-F1, sensitivity of 88.06%, and specificity of 94.49%, yet BanHateFormer matches or exceeds these results with over 63↓ fewer parameters, improving sensitivity by 1.76 points while drastically reducing model size. Meanwhile, our FFN variant depicts 87.07% accuracy, 85.77% precision, 86.52% recall, 86.11% macro-F1, 84.57% sensitivity, and 88.47% specificity, accentuating that the mixture-of-expert architecture is pivotal to capturing nuanced hate‐speech patterns in Bangla with both efficiency and state-of-the-art persuasiveness. Furthermore, we improved the semantic quality of the BanTH dataset by using a strategic prompting approach, avoiding the conventional back translation method. As shown in Table 4, this approach enables BanHateFormer to surpass both classical embedding techniques and several transformer-based baselines. Specifically, BanHateFormer achieves an accuracy of 82.46%, precision of 81.65%, recall of 81.92%, and a macro-F1 score of 81.78%. Additionally, it achieves sensitivity and specificity scores of 79.30% and 84.55%, respectively. These results demonstrate a + 1.50% augmentation in accuracy and a + 2.06% increase in macro-F1 compared to BanglaBERT, and represent refinements of +10.22%/ + 11.22% in accuracy/macro-F1 over GloVe embeddings, with a likewise large margin over Word2Vec and FastText. Even when compared against the 3B-parameter LLaMA-3.2-3B–which achieves an equivalent 82.63% accuracy and 81.66% macro-F1, BanHateFormer matches or exceeds these key metrics while utilizing over two orders of magnitude fewer parameters. During the annotation of the BanTH dataset, we preserved the transliterated text column with refined annotations to evaluate BanHateFormer’s performance on transliterated texts. As shown in Table 5, BanHateFormer outperforms baseline models, achieving 80.40% accuracy, 80.01% precision, 78.38% recall, and a macro-F1 score of 78.96%, along with 68.84% sensitivity and 87.92% specificity. In comparison, the feedforward variant acquires 76.26% accuracy, 75.51% precision, 73.87% recall, 74.40% macro-F1, 62.60% sensitivity, and 85.13% specificity, while TB-mBERT records 77.99% accuracy, 78.29% precision, 74.69% recall, 75.58% macro-F1, 59.55% sensitivity, and 89.84% specificity, underscoring the ability of capturing nuanced linguistic patterns in both native Bangla and codemixed (transliterated) text.

**Table 4:** The comparison of the quantitative performance of different existing methods across BanglaHateCorpus and BanTH datasets.

Method	#Params.↓	BanglaHateCorpus						BanTH					
		ACC	PR	RE	F1	Sensitivity	Specificity	ACC	PR	RE	F1	Sensitivity	Specificity
**Pre-trained Models**													
BanglaBERT	110.62M	90.23	90.06	88.60	89.24	82.68	94.52	80.96	80.29	79.64	79.92	73.21	86.07
RoBERTa	13.47M	88.33	87.41	87.29	87.35	83.51	91.07	78.27	77.68	76.31	76.80	66.78	85.85
m‑BERT	167.36M	89.52	89.72	87.37	88.33	79.59	95.14	79.61	78.74	78.59	78.66	73.61	83.57
HateBERT	167.36M	88.07	88.16	85.69	86.68	77.11	94.28	77.19	78.32	73.57	74.45	55.89	91.25
XLM‑RoBERTa	278.05M	89.40	88.50	88.57	88.53	85.58	91.56	80.45	79.56	79.84	79.69	76.86	82.81
Indic‑BERT	33.45M	87.85	87.12	86.36	86.71	80.98	91.75	78.64	78.26	76.48	77.06	65.90	87.05
LLaMA‑3.2‑3B	3B	90.18	90.68	87.93	89.03	79.82	96.08	82.62	82.10	81.33	81.66	75.03	87.63
GEMMA‑2‑2B	2B	**92.17**	**91.69**	91.28	**91.48**	88.06	**94.49**	**83.19**	**82.41**	**82.61**	**82.51**	**79.77**	**85.45**
**Our Proposed Methods**													
GloVe	31.83	79.98	78.29	79.36	78.71	77.15	81.57	72.24	71.01	70.30	70.56	60.83	79.78
Word2Vec	10.4M	82.50	82.27	78.91	80.07	66.13	91.70	74.37	73.26	72.77	72.97	64.95	80.58
FastText	10.391M	81.92	82.15	77.76	79.11	62.93	92.58	72.57	71.35	71.25	71.30	64.82	77.68
FFN	31.68M	87.07	85.77	86.52	86.11	84.57	88.47	77.57	76.75	77.56	76.99	77.54	77.59
BanHateFormer	**31.68M**	91.97	91.18	**91.50**	91.33	**89.82**	93.19	82.46	81.65	81.92	81.78	79.30	84.55

**Table 5:** The juxtaposition of qualitative performance of our proposed BanHateFormer on transliterated Bangla text across accuracy, precision, recall, and f1-score (macro) metrics.

Method	#Params.×	BanTH					
		ACC	PR	RE	F1	Sensitivity	Specificity
TB-mBERT [45]	**177.85M**	77.98	78.28	74.69	75.58	59.55	**89.84**
FFN	31.68M	76.26	75.51	73.87	74.40	62.60	85.13
**BanHateFormer**	31.68M	**80.40**	**80.01**	**78.38**	**78.96**	**68.84**	87.92

Our extensive qualitative experiments present three complementary strategies for efficient and robust Bangla hate‐speech classification. First, BanHateFormer was trained on BanglaHateCorpus, a corpus curated through a stringent multi-stage annotation pipeline, performs state-of-the-art performance (91.97% accuracy, 91.33% macro-F1) with merely 31.7M parameters, surpassing substantially larger transformer-based models.

Second, our strategic prompting method used for the BanTH dataset significantly enhances semantic fidelity, yielding 82.46% accuracy and 81.78% macro-F1, thereby outperforming both classical embedding approaches and transformer-based baselines. Third, BanHateFormer upholds robust performance on code-mixed inputs (80.40% accuracy, 78.96% macro-F1). Together, these findings exemplify that meticulous data curation, an efficient mixture of expert design, and targeted prompting can jointly deliver state-of-the-art hate-speech detection in Bangla underneath rigid resource limitations.

### Qualitative analysis

The qualitative performance of each expert in BanHateFormer is outlined in Table 6, indicating a separate division of labor across diverse sentence types. The expert utilization curves (Fig 11) for our MoE layer stay consistent at around 25% throughout training, exhibiting the tiniest variance. This consistency suggests that our gating network achieves balanced routing, preventing any expert from tumbling or starving. Such uniform load distribution ensures full engagement of all experts, fosters emergent specialization, and improves the model’s robustness, utilization capability, and generalization performance. For instance, Expert 1 acts as a “neutral gate,” routing benign or non-hateful words and correctly classifying them as negative. Expert 2 serves as a balanced workhorse, processing the majority of examples and adeptly managing both subtle insults and neutral statements without strong bias, but it often becomes overburdened. Expert 3 specializes in structural or punctuation-driven edge cases, triggering sentences with unconventional syntax or repeated tokens; yet, it is sometimes underutilized. Expert 4 acts as a “toxicity sentinel,” prioritizing overtly offensive or hateful content to identify the most severe instances. Nevertheless, it is unduly sensitive to long, idiomatic insults, often leading to false positives.

**Table 6:** The qualitative effectiveness of experts in our proposed BanHateFormer.

Input	Output	Assigned Expert
1: তুই সামনে যা। (You may go forward.)	NHS	Expert 1
2: কুত্তালীগ পুলিশ। (Police the dog league.)	HS	Expert 2
3: গণধোলাই দেওয় দরকার ছিলো। (A public beating was needed.)	NHS	Expert 3
4: মাগীর বেটির নাটক সব। (It is all a drama of this slut.)	HS	Expert 4

**Fig 11:**
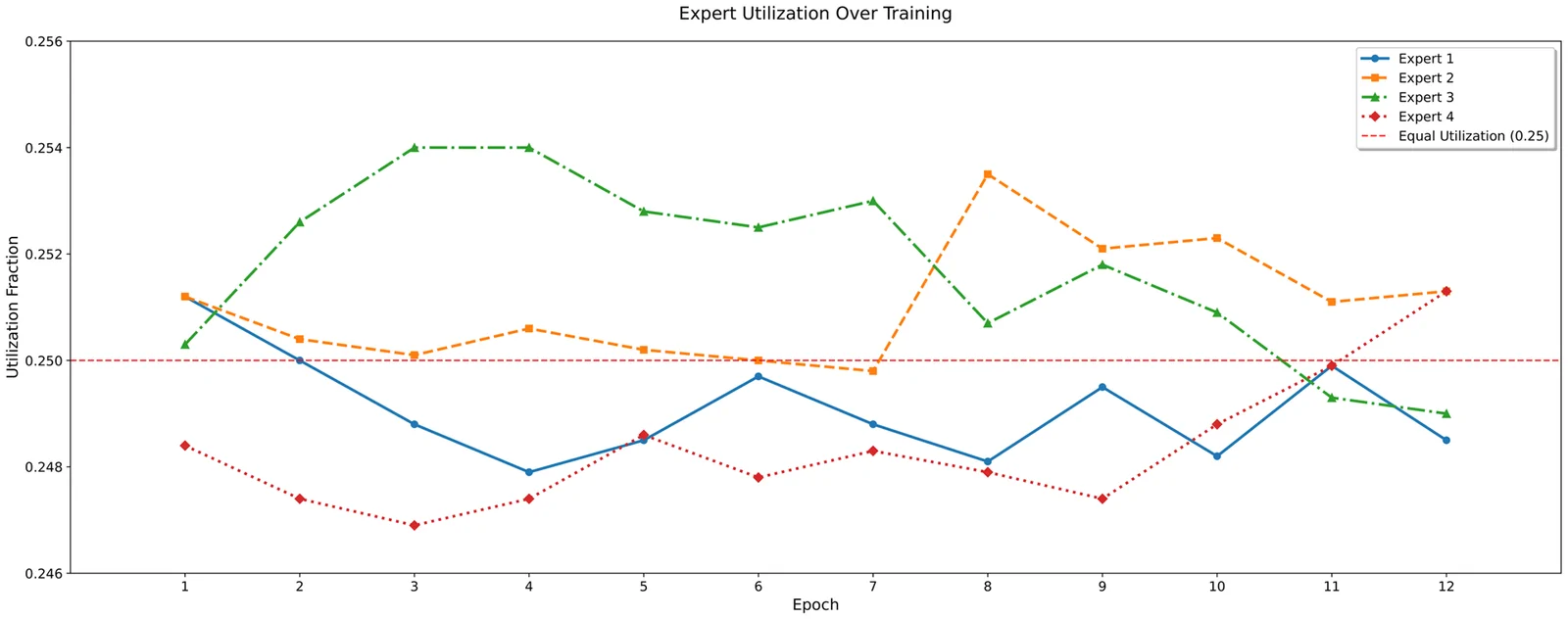
Expert utilization trajectories during the training of the BanHateFormer model. The line graph tracks the utilization fraction for four distinct experts over 12 training epochs. The dashed red horizontal line represents the ideal equal utilization threshold (0.25). The fluctuations demonstrate the dynamic allocation of computational load across experts, indicating that the model successfully avoids expert collapse while maintaining a balanced contribution from each specialized component throughout the learning process.

### Ablation study

In this through-ablation study, we investigated three pivotal dimensions of model efficiency: corpus size, the integration of traditional and modern transformer components, and the combination of different word embeddings within our proposed BanHateFormer architecture. In our BanHateFormer model, we applied RMSNorm immediately after the embedding layer and before rotary positional encoding on the query (*Q*) and key (*K*) vectors, thereby replacing absolute positional encoding. Residual connections were introduced after the multi-head attention layer to enhance training stability and substituted the conventional pointwise feed-forward network with a mixture of experts incorporating the SwiGLU activation function. By replacing all traditional Layer Normalizations with RMSNorm, BanHateFormer keeps the core flow of the Vaswani‐style [67] transformer encoder while improving computational efficiency by having a significantly smaller number of parameters.

Empirical results on BanglaHateCorpus and BanTH datasets reveal that BanHateFormer outperforms alternative configurations. Ablation experiments comparing static embeddings (Word2Vec [73], GloVe [74], or FastText [75]) with dynamic RoPE demonstrated inadequate performance of static methods, proving the dominance of adaptive positional encoding. Additionally, replacing the MoE layer with a standard feed-forward network validated the effectiveness of MoE in modern LLMs.

Furthermore, as depicted in Table 7 exhibits that scaling the BanglaHateCorpus training corpus from 50K to 156K instances yields progressively adequate performance, pinnacling in 91.97% accuracy and a 91.33% F1-macro score on the entire dataset. Specifically, training on a smaller corpus of 50K instances produced an accuracy of 84.87%, a precision of 84.48, a recall of 82.20, and an F1-Macro of 83.10. Expanding the corpus to 100K instances led to slight progress in all metrics, illustrating an increase of 1.98% in accuracy, 1.57 in precision, 3.03 in recall, and 2.50 in F1-Macro, respectively. Finally, further scaling the corpus to 156K instances resulted in more significant enhancements, with an increase of 7.10% in accuracy, 6.70 in precision, 9.30 in recall, and 8.23 in F1-Macro. These corpus scaling results directly motivate our choice of a 92.9/3.56/3.56 train-validation-test split over the conventional 80/10/10 heuristic. To empirically justify this departure, we conducted a controlled comparison between the two configurations. The standard 80/10/10 split, yielding a training set of 134,724 instances, achieved a Macro F1 of 88.81%, whereas our 92.9/3.56/3.56 split, with a training set of 156,406 instances, achieved a Macro F1 of 91.33%, representing a 2.52% absolute improvement. This gain is consistent with the corpus size ablation findings, which demonstrate that model performance in this morphologically rich, low-resource setting is highly sensitive to training volume. Rigidly adhering to the 80/10/10 heuristic in this context would induce what we term training data starvation, a condition where the model fails to adequately learn infrequent but semantically critical Bangla tokens, particularly rare slang, transliterated terms, and culturally specific expressions of hate.

**Table 7:** The influence of corpus size on the performance of our proposed BanHateFormer.

Method	Corpus	Inference			
	Size	Accuracy	Precision	Recall	F1-Macro
BanHateFormer	50K	84.87	84.48	82.20	83.10
BanHateFormer	100K	86.85	86.05	85.23	85.60
BanHateFormer	156K	91.97	91.18	91.50	91.33

To further validate that the reported performance is not inflated by any single constituent dataset, we conducted a full suite of leave-one-dataset-out (LODO) experiments across the three datasets comprising BanglaHateCorpus: BD-SHS, BanglaHateBERT, and BanTH. As reported in Table 8, training on BanglaHateBERT and BanTH while testing on BD-SHS yielded an F1 of 89.29%; training on BD-SHS and BanTH while testing on BanglaHateBERT produced the highest LODO F1 of 91.54%; and training on BD-SHS and BanglaHateBERT while testing on BanTH yielded the most challenging condition with an F1 of 87.65%. Across all three held-out conditions, BanHateFormer achieves a mean LODO F1 of 89.49%, with a narrow performance range of 87.65% to 91.54%, confirming that no single dataset disproportionately drives overall performance. Notably, even the lowest LODO F1 of 87.65% still outperforms several strong baseline models, demonstrating that the superiority of BanHateFormer is a structurally robust finding rather than a dataset-dependent artifact. The modest 1.84 percentage point gap between the mean LODO F1 (89.49%) and the full-training F1 (91.33%) is expected under reduced training volume and is entirely consistent with standard LODO behavior.

**Table 8:** Leave-one-dataset-out (LODO) cross-dataset generalization results of BanHateFormer.

Train Datasets	Test Dataset	Train Size	Inference			
			Accuracy	Precision	Recall	F1-Macro
BanglaHateBERT + BanTH	BD-SHS	47,114	89.23	88.54	90.17	89.29
BD-SHS + BanTH	BanglaHateBERT	76,353	91.49	90.87	92.31	91.54
BD-SHS + BanglaHateBERT	BanTH	56,239	87.66	86.94	88.45	87.65
**Mean**	–	–	**89.46**	**88.78**	**90.31**	**89.49**

The reliability of this test set is further grounded in formal statistical reasoning. With 6,000 test instances and a reported Macro F1 of 91.33%, the evaluation yields a 95% confidence interval of ±0.71%, ensuring that all reported performance improvements exceed the threshold of evaluation noise. Notably, the key performance advantage of BanHateFormer over the strongest baseline, BanglaBERT (89.24%), amounts to a 2.09 percentage point gain, which is 6.1 times larger than the margin of error, confirming its statistical validity. Similarly, the 2.30 percentage point improvement over LLaMA-3.2-3B (89.03%), a model with 94.74 times more parameters, exceeds the confidence interval by a factor of 6.76, further reinforcing the reliability of our findings. This level of statistical precision is comparable to, and in many cases exceeds, that of established NLP benchmarks such as WMT [76] and GLUE [77], which typically maintain high-confidence evaluations with test sizes often under 1% of the total corpus or fixed at fewer than 8,000 samples.

To further assess sensitivity to random initialization and confirm training stability, we conducted experiments across five independent random seeds. As reported in Table 9, Seed 42 (primary) achieved a Macro F1 of 91.33%, Seed 123 yielded the highest score of 91.48%, Seed 2024 produced 91.40%, Seed 99 recorded 91.26%, and Seed 7 obtained the lowest score of 90.79%, demonstrating a narrow performance band across all initializations. The resulting Macro F1 scores yield a mean of 91.25% and a standard deviation of ±0.27%, indicating that BanHateFormer exhibits strong and consistent training stability. The corresponding 95% confidence interval of [90.92%, 91.59%] provides a mathematically conclusive bound on the model’s true performance. Critically, even the lower bound of this interval (90.92%) outperforms the best baseline, BanglaBERT (89.24%), by 1.68 percentage points, demonstrating that the superiority of BanHateFormer holds unconditionally across all tested initializations and is not dependent on any particular random seed. This stability is a direct consequence of the large training set of 156,406 instances, which substantially reduces variance in gradient updates and produces consistently converged model states. Collectively, these findings, visualized in Figs 12–15, underscore the interplay between dynamic positional encoding, data volume, and MoE in optimizing model performance.

**Table 9:** Multi-seed stability analysis of BanHateFormer across five independent random initializations.

Seed	Inference
	F1-Macro (%)
Seed 42 (Primary)	91.33
Seed 123	91.48
Seed 7	90.79
Seed 2024	91.40
Seed 99	91.26
**Mean ± SD**	**91.25 ± 0.27**
**95% CI**	**[90.92, 91.59]**

**Fig 12:**
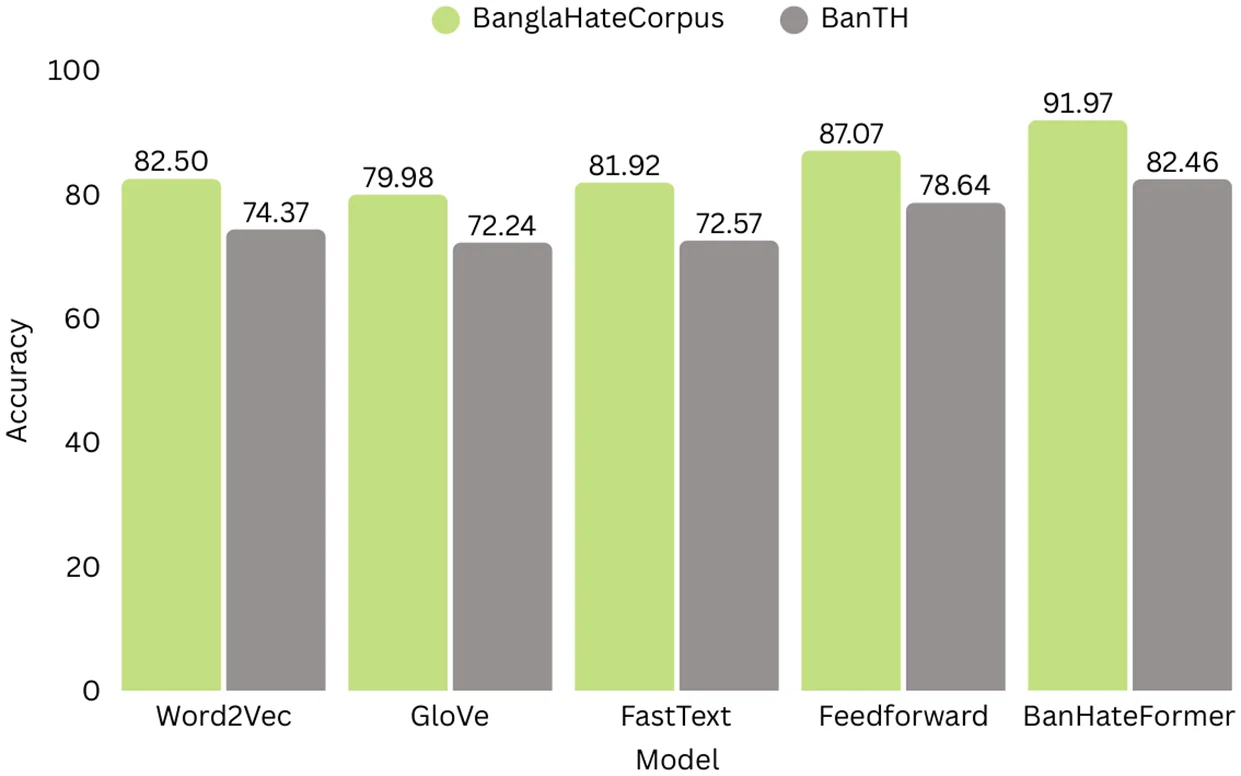
Comparative performance analysis of BanHateFormer against baseline models. The bar chart displays the accuracy scores for Word2Vec, GloVe, FastText, Feedforward, and the proposed BanHateFormer model on the BanglaHateCorpus and BanTH datasets. BanHateFormer achieves the highest classification fidelity with an accuracy of 91.97% on the BanglaHateCorpus, consistently outperforming traditional embedding-based and feedforward approaches across both evaluated datasets.

**Fig 13:**
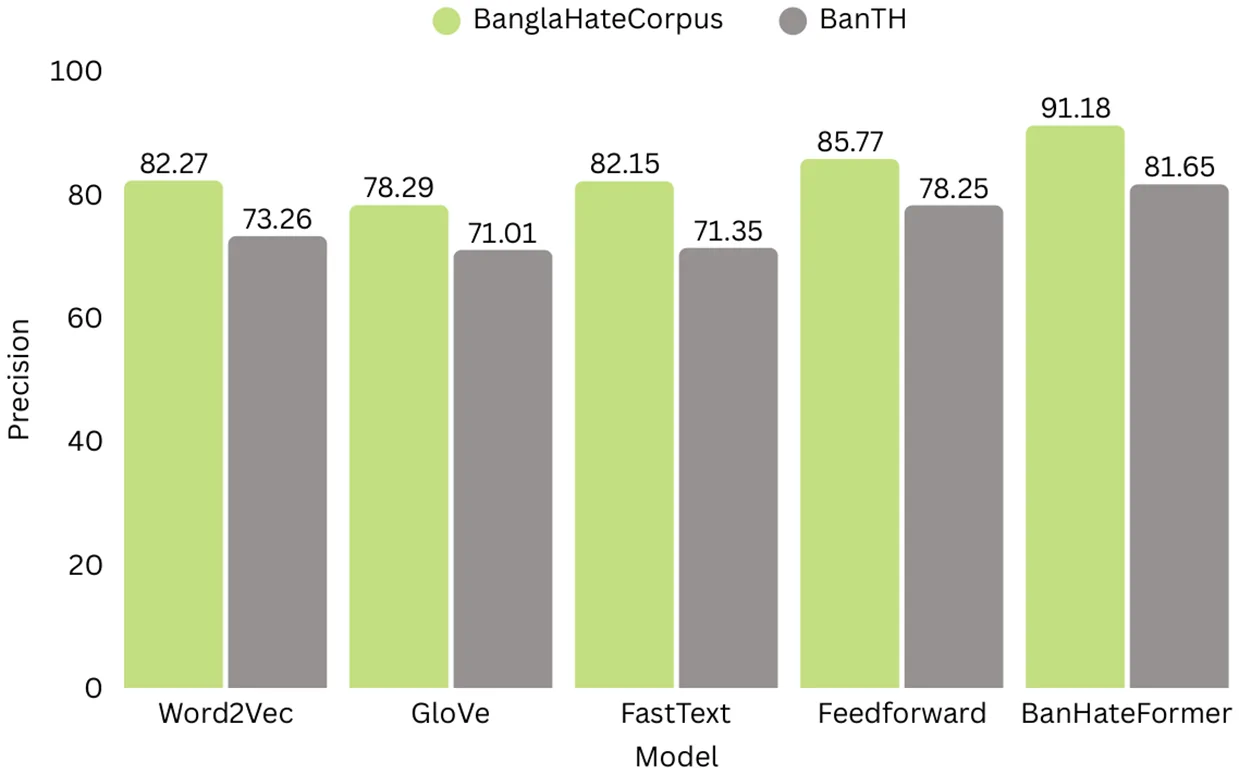
Comparative Precision analysis across baseline and proposed models. The bar chart illustrates the precision scores for various architectures on the BanglaHateCorpus and BanTH datasets. BanHateFormer achieves a peak precision of 91.18% on the BanglaHateCorpus, demonstrating a superior capacity for refined detection acuity and a significant reduction in false-positive rate compared to Word2Vec, GloVe, FastText, and Feedforward baselines.

**Fig 14:**
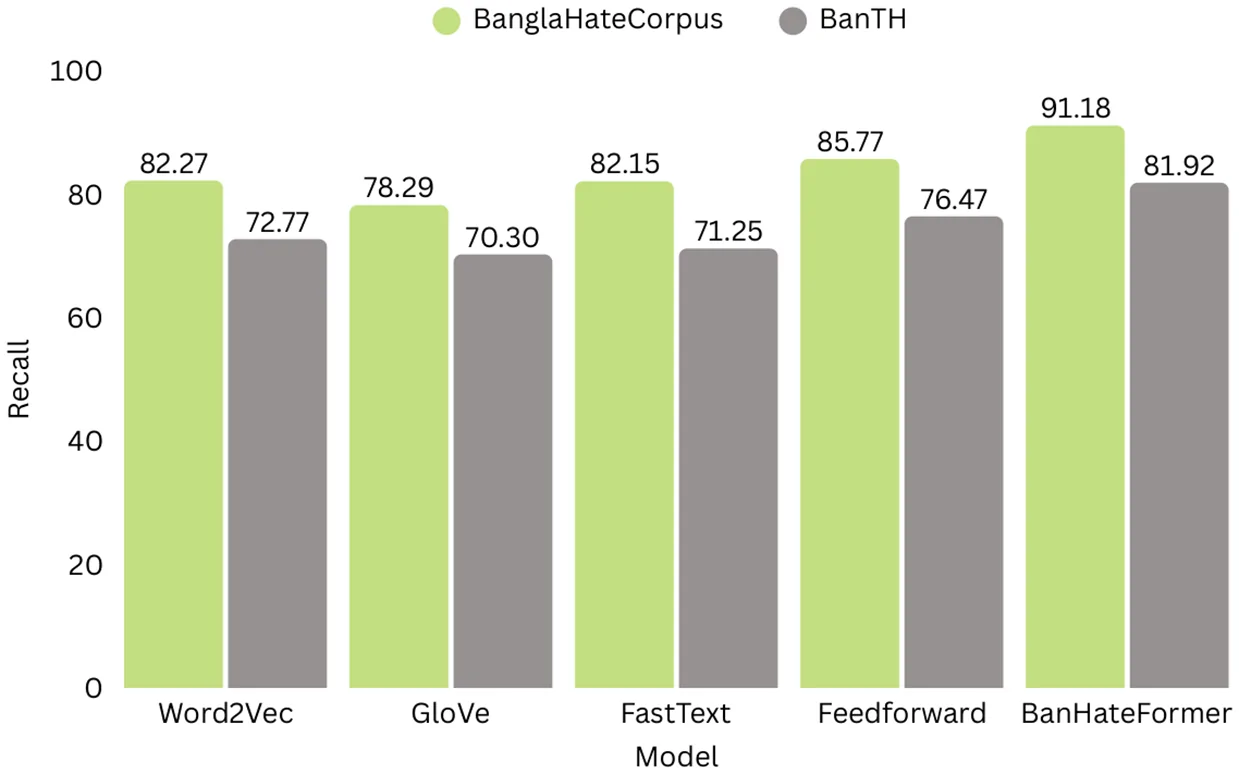
Comparative Recall metrics for baseline and proposed architectures. The bar chart illustrates the sensitivity of various models on the BanglaHateCorpus and BanTH datasets. BanHateFormer achieves a superior recall score of 91.18% on the BanglaHateCorpus, elucidating its adeptness in capturing a broader range of hate speech instances and demonstrating augmented correctness compared to traditional Word2Vec, GloVe, FastText, and Feedforward baselines.

**Fig 15:**
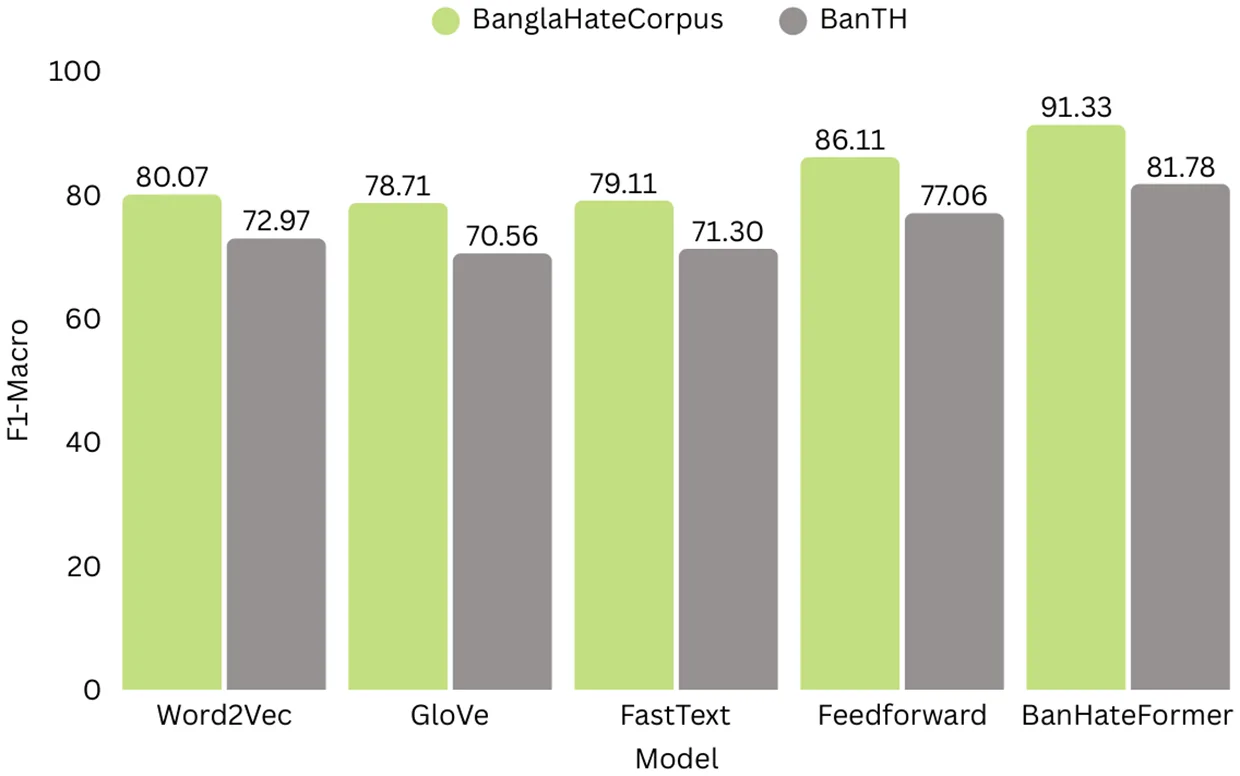
F1-Macro score trends for BanHateFormer and baseline models. The bar chart compares the macro-averaged F1 scores across the BanglaHateCorpus and BanTH datasets. BanHateFormer achieves a superior F1-Macro score of 91.33% on the BanglaHateCorpus, indicating enhanced classification performance and a robust balance between precision and recall across all categories compared to Word2Vec, GloVe, FastText, and Feedforward architectures.

This study underscores the critical significance of large-scale training data and modern architectural advances, particularly the integration of rotary positional encodings (RoPE) with mixture-of-experts (MoE) layers, in elevating classification accuracy and generalization. As corpus size increases, we observe a consistent upward trajectory across all performance metrics, a trend corroborated by both the corpus size ablation and the controlled train-test split comparison, wherein expanding the training set from 134,724–156,406 instances alone yielded a 2.52% absolute gain in Macro F1. The generalizability of BanHateFormer beyond its training distribution is further substantiated by the leave-one-dataset-out (LODO) experiments, wherein the model achieved a mean F1 of 89.49% across all three held-out conditions, with even the most challenging condition yielding an F1 of 87.65%, still outperforming several strong baselines. The narrow LODO performance range of 87.65% to 91.54% confirms that no single constituent dataset disproportionately drives model performance, establishing the robustness of BanHateFormer as a structurally grounded finding rather than a dataset-dependent artifact. The statistical robustness of these results is further confirmed by a 95% confidence interval of ±0.71% on the test set evaluation and a negligible standard deviation of ±0.27% across five independent random seeds, collectively ruling out the possibility that the reported gains are artifacts of favorable data partitioning or random initialization. Furthermore, the performance advantage of BanHateFormer over the strongest baseline exceeds the margin of error by a factor of 6.1, reinforcing the validity and reproducibility of our findings. Together, these results establish BanHateFormer as a statistically sound, practically efficient, and architecturally well-motivated solution for hate speech classification in morphologically rich, low-resource languages such as Bangla.

## Conclusion and future work

In this study, we present a potent monolingual deep learning baseline BanHateFormer for Bangla hate-speech classification, leveraging advanced pre-trained transformer architectures. In response to the task, we have developed BanglaHateCorpus, a large-scale, publicly available corpus that turns Bangla from a low-resource to a well-supported language for this task at hand. BanHateFormer surpasses LLaMA-3.2, BanglaBERT, and several other transformer-based baselines and emerges as a new state-of-the-art benchmark. However, our analysis demonstrates that BanHateFormer’s performance escalates significantly with larger training datasets, underscoring its dependence on ample resources and thereby motivating the research community to further expand our dataset. We also show that, despite recent advances, even top-tier Bangla language models still struggle with the subtle semantic and contextual requirements of hate-speech detection. Our work opens new avenues for efficient natural language processing in resource-constrained settings. Future research directions include knowledge distillation from multilingual models and the development of phrase/word-level annotations for more granular insights. Subsequently, a specific area for future work is the calibration of the router itself. Increasing the z-loss weight ($\lambda_{z}$) would help discourage the overly confident logits that currently allow Expert 4 to handle long, idiomatic insults, which it has not yet learned to differentiate from genuine hate speech. This adjustment could reduce its false-positive rate. Another complementary approach is to replace our soft load-balancing term with a hard, dynamic per-expert token capacity limit. This idea aligns with the capacity-factor mechanisms found in models like Switch Transformer [69] and ST-MoE [68], which would cap the number of tokens that Expert 2 can process in each training batch and redirect any excess to the relatively inactive Expert 3. We will explore these two calibration methods and their effects on per-expert precision and workload in future research. We believe that BanglaHateCorpus will be an invaluable resource for creating safer digital communities.
