# Supplementary material for: BanglaHateCorpus: A large-scale bangla benchmark for hate speech classification and a transformer-based deep learning method
Source: PLoS One. 2026 Nov 4;21(11):e0361339. doi: 10.1371/journal.pone.0361339 (PMC13734457; doi:10.1371/journal.pone.0361339)
Supplement: Supplementary file 1 [file pone.0361339.s001.pdf]

Original Manuscript ID: PONE-D-25-68240

Original Article Title: "BanglaHateCorpus: A Large-Scale Bangla Benchmark for Hate Speech Classification and a Transformer-Based Deep Learning Method"

In this document we have put each of the reviewer comments and corresponding response of ours.

Reviewer #1:

We sincerely thank Reviewer #1 for the thorough and constructive evaluation of our work, and for recognizing the contributions of our semi-supervised annotation pipeline, the breadth of our transformer-based experimentation, and the overall soundness of our research methodology. We are especially encouraged by the reviewer's positive assessment of the manuscript's write-up quality and the novelty of combining human expert annotation with LLM-based labeling for large-scale corpus construction. In response to the observations raised, we have carefully addressed each concern and believe the revisions meaningfully strengthen the clarity, rigor, and accessibility of the manuscript. A point-by-point summary of all changes is provided below.

Summary of response:

- We clarified the 94.74× parameter efficiency claim: the figure represents the ratio of LLaMA-3.2-3B's 3 billion parameters to BanHateFormer's 31.68 million parameters, and we have updated the abstract and introduction to make this computation explicit and unambiguous.
- We improved figure quality and legibility throughout the manuscript: all figures have been enlarged and standardized to match the resolution, font size, and graphical clarity of Figure 10, ensuring that architectural components, data labels, and trend lines are fully visible at publication scale.
- We justified the selection of Jaccard Similarity for deduplication: we explain its monotonic equivalence to Dice Coefficient, its superiority over Cosine Similarity for short-text lexical deduplication, and its empirical validation via Semantic Separation Rate (SSR = 0.024) and BERT Separation (0.0168) scores in Table 3.
- We expanded the Human Annotation section with full annotator qualifications, interdisciplinary selection rationale, and inter-annotator agreement statistics: Fleiss' Kappa scores of $\boldsymbol{K}$ = 0.8163 (hate) and $\boldsymbol{K}$ = 0.7289 (non-hate) confirm high consensus and methodological rigor.
- We resolved all bibliographic and formatting issues: 'last retrieved' dates have been added to all Hugging Face URLs, the broken "Fig ??" LaTeX reference has been corrected, and a comprehensive manuscript-wide proofread has been completed.
- We provided a formal justification for the influence normalization formula with a worked example and a supporting citation, and we clarified that the formula derives from established feature attribution practices in interpretability literature for linear classifiers.
- We empirically justified the 92.9/3.5/3.5 data split: a controlled 80/10/10 ablation yielded 88.81% Macro F1 versus 91.33% under our optimized split, a 2.52% absolute gain, corroborated by the Corpus Size Ablation in Table 7 and validated by the statistical robustness of our 6,000-instance test set (95% CI of ±0.71).
- We restructured and substantially expanded the background into a dedicated Related Work section incorporating state-of-the-art studies from 2024–2026, covering LLM-based hate speech detection, PAC-theoretic low-resource learning, ensemble methods, LoRA fine-tuning, and data augmentation paradigms, with seven newly integrated references.
- We conducted a full linguistic audit to ensure every instance of Bengali text in the manuscript is accompanied by phonetic transliteration and a faithful English translation, ensuring accessibility for readers unfamiliar with the Bengali script.

Reviewer #1, Concern #1 (How can 94.74x can be parameters. Is there some typo in the claimed contribution in introduction "state-of-the-art performance while using approximately 94.74× fewer parameters"?):

Author response: We appreciate the reviewer's careful attention to this detail, we would like to clarify that the "94.74×" figure is not a typo but represents the relative ratio of model parameters between our proposed architecture and a high-performing Large Language Model (LLM) baseline. We have clearly stated in the abstract that "BanHateFormer achieves state-of-the-art classification results, as assessed by accuracy, precision, recall, and macro F1-score with approximately 94.74 times fewer parameters than comparable models, achieving a 91.33% macro F1-score on the BanglaHateCorpus dataset and 78.96% on the transliterated BanTH benchmark." Specifically, while BanHateFormer utilizes an efficient architecture with only 31.68 million parameters, it achieves a Macro F1-score of 91.33%, which outperforms LLaMA-3.2-3B (with 3 billion parameters and an 89.03% F1-score). By dividing the 3 billion parameters of the baseline by the 31.68 million parameters of our model (3,000 / 31.68), we arrive at the factor of approximately 94.74x. This comparison is intended to highlight the extreme parameter efficiency of our sparse Mixture-of-Experts (MoE) approach, demonstrating that state-of-the-art performance in Bangla hate speech detection can be achieved with a model nearly two orders of magnitude smaller than the current state-of-the-art LLMs.

Reviewer #1, Concern #2 (Figure sized and content within figure is too small. Fig should be englarged for better visibility and quality should be improved. Fig 10 size and quality can be used for other figures too.):

Author response: We sincerely thank the reviewer for the feedback regarding figure legibility. In the revised manuscript, we have updated all figures to ensure consistent high resolution and improved visual clarity. Specifically, we have enlarged the figure dimensions and adjusted the internal font sizes and graphical elements to match the quality and scale of Figure 10. These modifications ensure that all architectural components, data labels, and trends are clearly visible and meet the publication standards for scientific presentation.

Reviewer #1, Concern #3 (Why Jaccard similarity is computed. Why not only metrics such as Dice Coefficient and Cosine Similarity are also considered for application?):

Author response: We thank the reviewer for this insightful question regarding our deduplication methodology. We selected Jaccard Similarity over the Dice Coefficient and Cosine Similarity based on mathematical properties, the specific constraints of short-form social media data, and empirical performance.

The Dice Coefficient and Jaccard Similarity are monotonically related via the identity:

$$Dice = \frac{2 * Jaccard}{1 + Jaccard}$$

Consequently, both metrics produce identical rankings for duplicate detection. We chose Jaccard as it remains the conventional standard in NLP for set-overlap deduplication.

Furthermore, a core objective of our pipeline is to "ensure semantic diversity" by removing lexical duplicates without collapsing topically distinct instances. Cosine Similarity measures semantic/vector equivalence. Using it for deduplication would inadvertently flag lexically distinct but topically similar hate speech (e.g., similar rhetoric targeting different groups) as duplicates. This would artificially homogenize the corpus. Jaccard Similarity, when applied to character sets, focuses on lexical exactness. This allows us to prune near-

verbatim noise while preserving the nuanced semantic variations necessary for a robust hate speech dataset. Social media text is often extremely short ($1 \leq l \leq 5$). Cosine Similarity is notoriously unstable in high-sparsity, short-text settings. To mitigate this, we computed Jaccard Similarity on character-level sets rather than word tokens. Combined with our dynamic thresholding (> 0.80 for $l \geq 5$; > 0.75 for $l < 5$), this approach is significantly more robust to the typographical variations and orthographic noise common in Bangla social media.

Moreover, our empirical results in Table 3 serve as evidence of the validation of this choice:

Our dataset achieves a Semantic Separation Score (SSR) of 0.024 and a BERT Separation of 0.0168, significantly outperforming benchmarks like BanTH and BD-SHS. We observed an average internal Jaccard similarity of 0.0153 (Hate) vs. 0.0106 (Non-Hate), with a clear between-class separation of 0.0047. These metrics demonstrate that our Jaccard-based approach successfully eradicated redundant noise while maintaining the high semantic diversity required for effective model training—a result that would likely have been suppressed by the more aggressive semantic filtering of Cosine Similarity.

Reviewer #1, Concern #4 (Details of human annotators are not given. How they were selected? Were they qualified and were having expertise to carry out the task. Has Kappa Statistics been applied to check whether they were correct to annotate the data?):

Author response: We thank the reviewer for emphasizing the importance of annotation transparency; we agree that explicitly detailing the annotators' qualifications and statistical validation significantly strengthens the methodology. To clarify details that were previously condensed, we have expanded the relevant Human Annotation section in the revised manuscript to explicitly justify our data annotation strategy. The five annotators were selected as native Bengali speakers to ensure the accurate capture of local slang, cultural references, and contextual phrasing. Recognizing hate speech as a deeply socio-cultural phenomenon, we intentionally assembled an interdisciplinary team of undergraduates from the Bangladesh University of Professionals: two Sociology students to evaluate societal prejudices and group dynamics, two Development Studies students to assess human rights contexts and the vulnerability of marginalized groups, and one English student to apply discourse analysis for detecting implicit threats, sarcasm, and dog-whistles, while also ensuring the precise comprehension of the English-language UNESCO framework [1] and United Nations definitions [2] of hate speech specifically the UN targets guideline [3] used for their training. Furthermore, to resolve ambiguous cases and ensure standardized evaluations, annotators were instructed to triangulate context using supplementary academic and institutional resources, including the Stanford Encyclopedia of Philosophy entry on hate speech [4], UNESCO's guidelines on school violence [5], and recognized definitions of cyberbullying from UNICEF [6] and the Cyberbullying Research Center [7]. Regarding the statistical validation of their work, we applied Fleiss' Kappa to measure inter-annotator agreement, yielding strong scores of $\boldsymbol{K}$=0.8163 for hate-speech samples and $\boldsymbol{K}$=0.7289 for non-hate samples, which confirms high consensus and validates the team's consistency. These scores have already been mentioned in the manuscript. The manuscript has been updated to seamlessly integrate these academic justifications and reliability metrics, ensuring our annotation process is fully transparent, reproducible, and grounded in interdisciplinary expertise.

References:

1. https://www.unesco.org/en/countering-hate-speech/need-know
2. https://www.un.org/en/hate-speech/understanding-hate-speech/what-is-hate-speech
3. https://www.un.org/en/hate-speech/impact-and-prevention/targets-of-hate
4. https://plato.stanford.edu/entries/hate-speech/
5. https://www.unesco.org/en/articles/what-you-need-know-about-school-violence-and-bullying
6. https://www.unicef.org/stories/how-to-stop-cyberbullying
7. https://cyberbullying.org/what-is-cyberbullying

Reviewer #1, Concern #5 (URLs like link of one source of hugging face is given. IT is recommended that last date retrieved should be mentioned. As these URLS are likely to get obsolete or change.):

Author response: We have updated the bibliography to include the 'last retrieved' date for all Hugging Face URLs to ensure archival clarity. Specifically, we have updated the citation for the titulm-gemma-2-2b-v1.1 model in the LLM as an Annotator and Judge subsection under the Data Annotation section.

Reviewer #1, Concern #6 (It is mentioned that "influence is calculated as (word coefficient/sum of positive coefficients) × 100.". What is the logic of using it? An working example with assumed values be used for computation process and to show the steps of computation for better understanding. Also if this is processed method by the author or they have taken from any other study, reference should be provided.):

Author response: We thank the reviewer for this thoughtful question. We address the logic, provide the clarification of the methodological origin below.

The Formula:

$$\text{Influence (\%)} = \frac{w_i}{\sum_{j\,:\,w_j>0} w_j} \times 100$$

is grounded in the interpretability literature for linear classifiers. In Logistic Regression, the coefficient w_i of a feature directly reflects its learned discriminative contribution to the log-odds of the target class. A larger positive coefficient means the model assigns a stronger push toward predicting "hate speech" when that word is present. Normalizing by the sum of all positive coefficients transforms raw coefficients into a relative contribution of the total positive discriminative signal learned by the model. What percentage does this single word contribute? This makes individual word contributions interpretable and comparable on a 0–100% scale, independent of the model's absolute weight magnitudes, which vary with regularization strength, dataset size, and feature scaling.

This normalization approach is consistent with well-established practices in feature attribution and model interpretability [1]. We have updated the manuscript to include all the necessary details.

[1] Waseem, Z., & Hovy, D. (2016). Hateful Symbols or Hateful People? Predictive Features for Hate Speech Detection on Twitter. In J. Andreas, E. Choi, & A. Lazaridou (Eds.), Proceedings of the NAACL Student Research Workshop (pp. 88–93). Association for Computational Linguistics. https://doi.org/10.18653/v1/N16-2013

Reviewer #1, Concern #7 (Such error shows that the proofread is requird, "The entire intrusion classification method is illustrated in Fig ??."):

Author response: We sincerely thank the reviewer for catching this oversight and apologize for the typographical and formatting errors. The broken figure reference ("Fig ??") was due to a LaTeX compilation issue that has now been resolved, correctly pointing to the intended figure in the updated manuscript. Furthermore, we have conducted a comprehensive and thorough proofreading of the entire manuscript to ensure that all terminology is accurate, references are properly linked, and the text is free of any similar typographical or formatting issues.

Reviewer #1, Concern #8 (Why test and validation division is merely less than 4 %.?"):

Author response: We thank the reviewer for the opportunity to empirically justify our data split. While an 80/10/10 ratio is a common heuristic, we found that rigidly adhering to it in low-resource, morphologically rich settings like Bangla leads to "training data starvation," where the model fails to capture infrequent but critical linguistic features. To address this, we conducted the 80/10/10 experiment: this split (134.7k train/16.8k test) achieved 88.81% Macro F1, whereas our 92.9/3.5/3.5 split (156.4k train/6k test) reached 91.33% which is a significant 2.52% absolute improvement. This result directly corroborates our Corpus Size Ablation (Table 7), where expanding the training set from 100k to 156k yielded a +5.73% F1 boost, proving that model generalization in this domain is highly sensitive to training volume. Furthermore, our 6,000-instance test set is not a limitation but a statistically robust choice; at a 91% performance level, it yields a 95% confidence interval of ±0.71, ensuring that our reported gains are mathematically conclusive and not artifacts of evaluation noise. This strategy of prioritizing training density over excessively large test sets aligns with established benchmarks like WMT and GLUE, which maintain high-confidence evaluations with test allocations often under 1% or fixed at <8,000 samples. Consequently, our split represents an empirically optimized architecture that maximizes the model's exposure to rare Bangla tokens while maintaining a rigorous, bulletproof evaluation standard.

Reviewer #1, Concern #9 (There is no related work but background is provided. Need to discuss the review of latest publications from years 2024-6 in this section to highlight latest work in the corpus and sentiment analysis.

doi: 10.1145/3773075

doi: 10.1109/TCE.2026.3652186

doi: https://doi.org/10.1016/j.knosys.2025.115123):

Author response: We thank the reviewer for this important point. We have expanded the background section into a comprehensive "Related Work" section, incorporating a review of state-of-the-art studies from 2024 to 2026. This updated discussion specifically highlights recent advancements in Bengali hate speech detection, Large Language Models (LLMs), and theoretical frameworks for low-resource data. We have also incorporated these recent studies into our related work, which are as follows.

Transformer models, particularly BERT and its variants, have significantly advanced NLP, with a profound impact on hate speech detection in Bangla despite its low-resource status; more recently, Large Language Models (LLMs) and TinyLLMs have also emerged as promising tools as discussed by Albadi et al. [1].

Such constraints are increasingly understood within broader theoretical paradigms as well; rigorous PAC framework analyses indicate that learning with low-resource data involves inherent trade-offs in generalization error and label complexity that simply scaling standard models may not strictly overcome, as mentioned by Cao et al. [2].

Extreme ensemble usage remains prominent, as evidenced by recent 2025 shared tasks introduced by Hasan et al. [3], where some leading teams relied on heavy multi-task transformer ensembles and weighted probabilistic fusions by Alam et al. [4] to overcome fragmented sub-datasets. The primary trade-off with such ensemble methods, however, is significantly increased computational cost and parameter bloat, motivating the need for more parameter-efficient standalone architectures.

Beyond pure classification, the capacity of LLMs to bridge semantic gaps via targeted data augmentation serves as an effective mechanism across diverse domains, including multimodal ranking and text analysis, as shown by Li et al. [5].

More recent investigations have evaluated LLMs using multi-task LoRA fine-tuning for severity and targeted entity identification, noting that while LLMs adapt well to low-resource settings, culturally-grounded pretraining retains a qualitative edge, which is demonstrated by Hasan et al. [6].

Lastly, the study by Babu et al. [7] employed BERT after translating Bangla to English, achieving 95% accuracy; yet, this was lower than their RNN-based models on the same translated data and inherently risks loss of original nuance as well as encountering synthetic generation artifacts known to disrupt the original structural calibration of the text, as mentioned by Yin and Wang [8].

[1] Hate Speech Detection Using Large Language Models: A Comprehensive Review.

[2] Analytical Survey of Learning with Low-Resource Data: From Analysis to Investigation.

[3] Catalyst at BLP-2025 Task 1: Transformer Ensembles and Multi-task Learning Approaches for Bangla Hate Speech Detection.

[4] CoU-CU-DSG at BLP-2025 Task 1: Leveraging Weighted Probabilistic Fusion of Language Models for Bangla Hate Speech Detection.

[5] AugMMRev: An LLM-Augmented Multimodal Ranking Model for Personalized Image Material Retrieval.

[6] LLM-Based Multi-Task Bangla Hate Speech Detection: Type, Severity, and Target.

[7] Bangla Hate Speech Detection System Using Transformer-Based NLP and Deep Learning Techniques.

[8] Span-level detection of AI-generated scientific text via contrastive learning and structural calibration.

Reviewer #1, Concern #10 (It should be checked again that no Bangla content is in the manuscript without English translation as otherwise, it will be helpful for understanding of the readers.):

Author response: We thank the reviewer for highlighting the importance of linguistic accessibility for a general NLP audience. We fully agree that providing English translations for all non-English examples is essential for the readability and impact of the work. In response to this feedback, we have conducted a comprehensive manual audit of the manuscript to ensure that every instance of Bengali text which includes dataset samples, error analysis cases, and qualitative examples that is accompanied by a faithful English translation. We have specifically standardized the format to include the original script, a phonetic transliteration (where relevant for morphosyntactic discussion), and a literal or idiomatic English translation. These additions ensure that readers unfamiliar with the Bengali script can fully grasp the semantic nuances and linguistic challenges addressed in our methodology and results. We have updated the manuscript according to the above concern.

Reviewer #2:

We sincerely thank Reviewer #2 for the thorough and rigorous evaluation of our manuscript, and for acknowledging the significance of our large-scale unified corpus and the parameter efficiency of BanHateFormer as a core contribution to Bangla hate speech classification. We welcome the recommendation for Major Revision and have treated each concern as an opportunity to substantially strengthen the methodological transparency, statistical rigor, reproducibility, and ethical grounding of the work. We are confident that the comprehensive revisions described below, all of which are fully reflected in the updated manuscript, collectively address every dimension of the reviewer's critique and bring the submission into full alignment with PLOS ONE's editorial and scientific standards.

Summary of response:

- We have provided clear theoretical and empirical justifications for unifying toxicity and cyberbullying datasets within a hate speech classification framework. This rationale is supported by three key points: linguistic equivalence, empirical co-occurrence, which is influenced by the online disinhibition effect, and improved model robustness across 17 distinct hate categories. All of this information is formally documented in the revised Corpus Creation section.
- We substantially expanded the hybrid annotation pipeline to meet full reproducibility standards: the revised manuscript now documents exact model versions (TituLM-Gemma-2-2B-v1.1 and Gemini 2.0 Flash), complete prompt specifications (five-step transliteration and seven-step hate speech annotation protocols), adjudication criteria (consensus-first with Gemini as designated Judge), and the raw agreement rate of 87.5% across 165,406 instances.
- We reported comprehensive inter-annotator reliability metrics at both human and LLM levels: Fleiss' Kappa of $\boldsymbol{K}$ = 0.8163 (hate) and $\boldsymbol{K}$ = 0.7289 (non-hate) for human annotators, and Cohen's Kappa of $\boldsymbol{K}$ = 0.7402 between Gemma-2 and Gemini 2.0 Flash, providing dual-layer statistical validation of label trustworthiness across the entire corpus.
- We introduced multi-seed analysis across five independent random seeds, reporting a mean Macro F1 of 91.25% ± 0.27% and a 95% confidence interval of [90.92%, 91.59%], confirming that BanHateFormer's performance gains over all baseline models, including the 2.09 percentage point improvement over BanglaBERT, comfortably and conclusively exceed interval bounds and cannot be attributed to favorable random initialization.
- We provided full ablation study evidence isolating each architectural component: replacing MoE with a standard FFN caused a 5.22 percentage point drop in Macro F1 (91.33% → 86.11%); RoPE outperformed all static embedding baselines; RMSNorm matched or exceeded Layer Normalization at reduced computational cost; and corpus size scaling from 100K to 156K yielded a +5.73% F1 boost, demonstrating that training volume is architecturally necessary rather than merely additive.
- We conducted a structured, three-level error analysis covering human annotation adjudication of borderline cases, qualitative characterization of all four MoE expert failure modes (including Expert 4's documented over-sensitivity to idiomatic insults), and quantitative word-influence visualization in Figures 8 and 9 to expose systematic lexical biases at the model's decision boundary.
- We provided full transparency on the data-splitting methodology, including stratified class preservation (37.9% hate / 62.1% non-hate across all partitions) and a controlled empirical comparison showing that our 92.9/3.56/3.56 split outperforms the standard 80/10/10 heuristic by 2.52% absolute Macro F1, with a statistically defensible 6,000-instance test set yielding a 95% CI of ±0.71%.
- We satisfied PLOS ONE's data availability requirements by publicly releasing the processed BanglaHateCorpus, the BanTH processed dataset, all training scripts, model weights, and full documentation with licensing information via our dedicated repository at https://github.com/Rodelaa00/BanglaHateCorpus.

Dataset links:

1. BD-SHS,
2. BanTH,
3. BN-Hate,
4. Bengali Hate Speech Dataset,
5. Bengali Multi-Labeled Toxic Comments Dataset,
6. Vio-Lens, Bangla Cyberbullying Comments,
7. Bengali Cyber Bullying Dataset

All data is publicly available.

https://github.com/Rodelaa00/BanglaHateCorpus

Dataset links:

1. https://aclanthology.org/2022.lrec-1.552/

2. https://aclanthology.org/2025.findings-naacl.403/

3. https://aclanthology.org/2022.restup-1.2/

4. https://www.kaggle.com/datasets/naurosromim/bengali-hate-speech-dataset

5. https://github.com/deepu099cse/Multi-Labeled-Bengali-Toxic-Comments-Classification

6. https://huggingface.co/datasets/faisalahmed/Bengali_Cyberbullying_Detection_Comments_Dataset/tree/main

7. https://www.kaggle.com/datasets/moshiurrahmanfaisal/bangla-cyber-bullying-dataset

- We performed a full manuscript-wide terminology audit, defined all abbreviations at first use, updated the architectural diagram (Figure 10), and expanded the Methods section with step-by-step workflow descriptions to ensure scientific clarity and presentation consistency throughout.
- We documented full ethical compliance, including informed consent waiver for publicly available social media data, complete PII anonymization in compliance with platform Terms of Service, and annotator protections comprising informed consent, mandatory breaks, opt-out rights, and industry-standard compensation.

Reviewer #2, Concern #1 (Technical Soundness and Validity of the Conclusions

The manuscript presents a solid approach and a well-articulated motivation. The unification of eight datasets is valuable, but it requires additional transparency, particularly regarding the integration of toxicity and cyberbullying data when the stated objective is hate-speech classification.

The hybrid human–LLM annotation pipeline is innovative; however, it requires reproducible detail, including exact model versions, parameters, prompts, and agreement criteria.

The proposed BanHateFormer model appears promising in terms of efficiency, yet complete quantitative evidence comparing it with baseline models—along with robust statistical testing—is currently lacking.):

Author response: We sincerely thank the reviewer for their thorough and constructive evaluation. The concerns raised regarding annotation transparency, pipeline reproducibility, and statistical rigor are well-

founded, and we have addressed each point comprehensively below. We believe these clarifications, all of which are now reflected in the revised manuscript, substantially strengthen the scientific validity of our work.

1. **Unification of Toxicity and Cyberbullying Data within a Hate Speech Corpus**

We acknowledge that the rationale for integrating toxicity and cyberbullying datasets into the BanglaHateCorpus required more explicit justification. This unification is a deliberate and theoretically grounded design decision, supported by three converging lines of evidence:

(i) Linguistic Equivalence: Toxicity and hate speech share a common vocabulary of online aggression and overlapping linguistic patterns, making their joint representation appropriate for a unified classification framework [1].

(ii) Psychological and Empirical Correlation: Empirical research demonstrates that cyberbullying and cyberhate are positively correlated and frequently co-occur, a phenomenon explained by the "online disinhibition effect" [2]. Treating them as entirely separate phenomena would therefore introduce artificial distinctions that do not reflect real-world online discourse.

(iii) Enhanced Model Robustness: By treating toxicity as a comprehensive umbrella category, the model learns to recognize the toxic contextual signals that routinely precede or co-occur with identity-based attacks. This unified approach spans 17 predefined fine-grained categories such as Xenophobia, Racism, Sexual Hate, Religious Hate, Homophobia, Personal Insults, Geopolitical Issues, Sports, Entertainment, Crime, Politics, TikTok/Memes, Vulgarity, Threats, Trolling, Insults, and Body Shaming are thereby enabling the model to distinguish between subtle nuances while maintaining high precision in binary classification.

The revised manuscript now makes these justifications explicit in the Corpus Creation section.

2. **Reproducibility of the Hybrid Human–LLM Annotation Pipeline**

We fully agree that transparency in hybrid annotation is a prerequisite for scientific validity. We have substantially expanded the relevant sections of the revised manuscript to provide complete reproducibility information across three dimensions: annotator selection, LLM pipeline specifications, and agreement criteria which will be found in the corpus creation section.

2a. Human Annotator Selection and Qualifications

The five annotators were selected as native Bengali speakers to ensure accurate capture of local slang, cultural references, and contextual phrasing. Recognizing hate speech as a deeply socio-cultural phenomenon, we intentionally assembled an interdisciplinary team of undergraduates from the Bangladesh University of Professionals: Two Sociology students to evaluate societal prejudices, group dynamics, and systemic discrimination; Two Development Studies students to assess human rights contexts and the vulnerability of marginalized groups; One English student to apply discourse analysis for detecting implicit threats, sarcasm, and dog-whistles, and to ensure precise comprehension of the English-language definitional frameworks used for annotator training. Annotators were trained using internationally recognized frameworks, including the UNESCO guidelines on countering hate speech [3], the United Nations definition of hate speech [4], the UN targets guideline for identifying vulnerable groups [5], and the Stanford Encyclopedia of Philosophy entry on hate speech [6]. To resolve ambiguous cases and ensure standardized evaluation, annotators were further instructed to triangulate context using supplementary resources, including UNESCO's guidelines on school violence and bullying [7], and recognized cyberbullying definitions from UNICEF [8] and the Cyberbullying Research Center [9].

2b. LLM Model Versions, Parameters, and Prompts

The pipeline utilized two LLMs operating in a dual-annotator configuration:

Gemma-2 (TituLM-Gemma-2-2B-v1.1): Fine-tuned on 3,000 high-quality human-annotated samples (1,500 hate, 1,500 non-hate) to impart domain-specific linguistic competence and reduce class bias prior to pseudo-labeling the remaining 165,406 instances.

Gemini 2.0 Flash: Served as an independent second annotator and final adjudicator, selected for its advanced understanding of the Bangla language and multi-step reasoning capabilities. The pipeline employed two precisely documented prompts, both reproduced in full in the supporting materials: Transliteration Prompt: A five-step sequence, Read, Convert, Verify, Correct, and Output which is governed by four explicit constraints: Meaning, Consistency, Orthography, and Pronunciation. Hate Speech Annotation Prompt: A seven-step annotation guideline grounded in the UN Strategy and Plan of Action on Hate Speech [3–7], comprising: (1) verification of identity-based targeting across protected attributes (religion, ethnicity, nationality, race, gender, etc.); (2) identification of specific hate speech forms including scapegoating, stigmatization, and dehumanization; (3) consideration of socio-cultural context and Bangla-specific "dog whistles"; (4) severity assessment differentiating incitement from personal threats; (5) differentiation of legitimate criticism or satire from hate speech; (6) application of a three-tiered test evaluating Intent, Content, and Consequence; and (7) a mandatory Self-Critique step requiring the model to articulate its rationale and consider alternative interpretations, thereby mitigating inherent LLM biases.

2c. Agreement Criteria and Conflict Resolution Mechanism

The pipeline employed a structured "consensus-first" adjudication protocol: Automatic Acceptance: Labels were accepted without further review when both Gemma and Gemini independently agreed on the classification. This occurred in 144,677 of 165,406 instances, yielding an 87.5% agreement rate.

Conflict Resolution: In the remaining 12.5% of disagreement cases, Gemini was invoked as the designated "Judge," re-evaluating the sample via its step-by-step transparent reasoning process, with its final judgment adopted as ground truth and further verified by subject matter experts (SMEs).

Inter-Annotator Reliability (Human): Fleiss' Kappa yielded $\boldsymbol{k}$ = 0.8163 for hate-speech samples and $\boldsymbol{k}$ = 0.7289 for non-hate samples, indicating strong consensus and validating labeling consistency.

Inter-Annotator Reliability (LLM–LLM): Cohen's Kappa of 0.7402 between Gemma and Gemini confirms substantial agreement between the two automated annotators, providing an independent reliability check on the pseudo-labeled data.

All of these parameters, prompts, model versions, and adjudication criteria are now fully documented in the revised manuscript and supplementary materials to ensure complete reproducibility.

3. Quantitative Evidence and Statistical Robustness

We address this concern through four layers of quantitative evidence now fully reported in the manuscript.

3a. Comprehensive Benchmarking Against Baseline Models

BanHateFormer is evaluated against 12 baseline models across two benchmarks, BanglaHateCorpus and BanTH, which include eight transformer architectures (BanglaBERT, mBERT, XLM-RoBERTa, and others) and high-parameter LLMs (LLaMA-3.2-3B and Gemma-2-2B). As reported in Table 4: BanHateFormer (31.68M parameters) achieves a 91.33% Macro F1-score on BanglaHateCorpus. This outperforms the domain-specific BanglaBERT by 2.09 F1 points (89.24%) and the 3B-parameter LLaMA-3.2 (89.03%) while being 94.74 times smaller in parameter count. Evaluation is multidimensional, reporting Precision, Recall, Macro F1, Sensitivity, and Specificity to provide a robust characterization of performance under potential class imbalance.

3b. Ablation Studies as Structural Statistical Validation

The ablation study systematically isolates the contribution of each architectural component:

Mixture-of-Experts (MoE): Replacing the MoE layer with a standard feed-forward network (FFN) yields a measurable decline in F1-score across all categories, confirming that the MoE design is pivotal for capturing nuanced hate speech patterns.

Positional Encoding: Comparisons between static embeddings (Word2Vec, GloVe, FastText) and dynamic RoPE demonstrate the statistical superiority of adaptive positional encoding for this task.

Scalability Testing: Model performance is tracked as training corpus size scales from 50K to 156K samples, providing empirical evidence of reliability and data-volume dependence.

Additionally, the Expert Utilization Analysis (Fig. 11) confirms that all four experts maintain a stable 25% utilization rate, demonstrating balanced routing without expert collapse, and verifying that performance gains derive from genuine architectural efficiency rather than evaluation artifact.

3c. Statistical Rigor in Dataset Construction

The quantitative validity of the evaluation rests upon the statistical soundness of the dataset itself:

Human Label Validity: Fleiss' Kappa scores of $\boldsymbol{k}$ = 0.8163 (hate) and $\boldsymbol{k}$ = 0.7289 (non-hate) confirm the trustworthiness of the gold standard annotations.

LLM Label Consistency: Cohen's Kappa of 0.7402 between the two LLM annotators validates the pseudo-labeled majority of the corpus.

Class Separability: BERT Separation Score (BERT Sep.) and Lexical Separation Score (LSR) statistically demonstrate that BanglaHateCorpus achieves a class-separation score of 0.0168, compared to only 0.0009 in the prior BanTH benchmark, confirming that the dataset's classes are significantly more semantically distinct and thus conducive to more meaningful model evaluation.

3d. Cross-Dataset Generalization

BanHateFormer's robustness is further validated on the BanTH benchmark, which consists of noisy, transliterated (Romanized) Bangla text. BanHateFormer improves the macro F1-score by 3.38% over the specialized TB-mBERT baseline, demonstrating quantitative superiority even on script-mixed, out-of-distribution inputs.

We believe that the expanded annotation transparency, fully documented LLM pipeline, and multi-layered quantitative evidence collectively address all three concerns raised. The corresponding sections have been revised and expanded in the manuscript. We hope these clarifications demonstrate the rigor and reproducibility of our methodology, and we remain available to provide any further details the reviewer may require.

References

[1] Risch J. Toxicity. In: Strippel C, et al., editors. Challenges and perspectives of hate speech research. Digital Communication Research, vol. 12. Berlin; 2023. p. 219–230.

[2] Aljasir S. Effect of online civic intervention and online disinhibition on online hate speech among digital media users. Online Journal of Communication and Media Technologies. 2023;13(4):e202344.

[3] https://www.unesco.org/en/countering-hate-speech/need-know

[4] https://www.un.org/en/hate-speech/understanding-hate-speech/what-is-hate-speech

[5] https://www.un.org/en/hate-speech/impact-and-prevention/targets-of-hate

[6] https://plato.stanford.edu/entries/hate-speech/

[7] https://www.unesco.org/en/articles/what-you-need-know-about-school-violence-and-bullying

[8] https://www.unicef.org/stories/how-to-stop-cyberbullying

[9] https://cyberbullying.org/what-is-cyberbullying

Reviewer #2, Concern #2 (2. Statistical Analysis and Quantitative Rigor

The evaluation must include confidence intervals, statistical significance testing, and multi-seed analysis.

Ablation studies for each architectural component are necessary, as well as a detailed error analysis, particularly for borderline or ambiguous cases.

The data-splitting methodology must be fully clarified, including any stratification techniques used.):

Author response: We thank the reviewer for these precise and technically substantive concerns. We fully agree that confidence intervals, significance testing, multi-seed analysis, ablation studies, error analysis, and data-splitting transparency are all essential components of rigorous machine learning research. We address each sub-concern systematically below, with all corresponding updates reflected in the revised manuscript.

We acknowledge that the initial submission did not explicitly report confidence intervals or multi-seed variance analysis, and we have rectified this in the revised manuscript.

Confidence Intervals: Our test set comprises 6,000 instances, which is a deliberate and statistically principled choice. At the reported performance level of 91.33% Macro F1, this test set size yields a 95% confidence interval of ±0.71%, ensuring that all reported performance gains are mathematically conclusive and cannot be attributed to evaluation noise. This level of statistical precision is comparable, and in many cases exceeds that of established NLP benchmarks such as WMT and GLUE, which routinely maintain high-confidence evaluations with test allocations often under 1% of the total corpus or fixed at fewer than 8,000 samples.

Multi-Seed Analysis: To further validate performance stability, we conducted experiments across five independent random seeds. The results are reported in the table below:

Seed	Macro F1-Score (%)
Seed 42 (Primary)	91.33
Seed 123	91.48
Seed 7	90.79
Seed 2024	91.40
Seed 99	91.26
Mean ± SD	**91.25 ± 0.27%**
95% CI	**[90.92%, 91.59%]**

The low standard deviation of ±0.27% across all five seeds confirms that BanHateFormer exhibits strong training stability and that its reported performance is not an artifact of a favorable random initialization. Furthermore, the 95% confidence interval of [90.92%, 91.59%] provides a mathematically conclusive bound on the model's true performance, demonstrating that the reported gains over baseline models, which include the 2.09 percentage point improvement over the domain-specific BanglaBERT, comfortably exceed the interval bounds and cannot be attributed to random seed variance. This stability is a direct consequence of

the large training set size (156,406 instances), which substantially reduces variance in gradient updates and produces consistently converged model states across different initializations. These results have been added to the revised manuscript.

Inter-Annotator and Inter-Model Statistical Agreement: The statistical validity of the underlying evaluation data is further supported by two formal agreement metrics:

- **Fleiss' Kappa (Human Annotators):** k = 0.8163 for hate-speech samples and k = 0.7289 for non-hate samples, both indicating strong consensus and confirming the trustworthiness of the gold standard labels.
- **Cohen's Kappa (LLM–LLM):** k = 0.7402 between Gemma-2 and Gemini 2.0 Flash, with an overall 87.5% raw agreement rate (144,677 of 165,406 instances), validating the consistency of the large-scale pseudo-labeled data.

Dataset Class Separability: To statistically validate that the BanglaHateCorpus supports meaningful classification rather than spurious pattern matching, we report two class-boundary metrics:

- **BERT Separation Score (BERT Sep.):** BanglaHateCorpus achieves 0.0168, compared to only 0.0009 for the prior BanTH benchmark, and an approximately 18.7× improvement in semantic class separability.
- **Lexical Separation Score (LSR):** Corroborates the BERT-based finding, confirming that the two classes in our corpus are substantially more linguistically distinct, which is a prerequisite for reliable classifier evaluation.

Ablation Studies for Each Architectural Component

The manuscript includes a dedicated and comprehensive ablation study (Section Ablation Study) that systematically isolates and quantifies the contribution of each architectural component of BanHateFormer. The key findings are as follows:

Mixture-of-Experts (MoE) vs. Standard Feed-Forward Network (FFN): Replacing the MoE layer with a conventional FFN caused the Macro F1-score to drop from 91.33% to 86.11%**,** which is a statistically meaningful degradation of 5.22 percentage points. This result conclusively demonstrates that the MoE architecture is pivotal for capturing the nuanced, multi-dimensional linguistic patterns characteristic of Bangla hate speech, rather than being an incidental design choice.

Expert Utilization and Routing Stability: The Expert Utilization Analysis (Fig. 11) confirms that all four internal experts maintain a stable 25% utilization rate throughout training and inference. This balanced routing provides two critical statistical assurances: (i) the model does not suffer from expert collapse, a known failure mode in MoE architectures where a single expert dominates routing; and (ii) each expert has genuinely specialized in distinct linguistic sub-tasks, meaning performance gains are attributable to architectural efficiency rather than parameter bloat or evaluation artifact.

Positional Encoding RoPE vs. Static Embeddings: Ablation experiments comparing dynamic Rotary Positional Encoding (RoPE) against three static embedding baselines (Table 4), such as Word2Vec, GloVe, and FastText, demonstrate the statistical superiority of RoPE for this task. Static embeddings, which lack the ability to model long-range syntactic dependencies, are particularly inadequate for morphologically rich Bangla text, where positional context is semantically critical.

Normalization Strategy RMSNorm vs. Layer Normalization: The ablation study also justifies the replacement of traditional Layer Normalization with RMSNorm, demonstrating that RMSNorm achieves equivalent or superior model fidelity while reducing computational overhead, a particularly valuable property for deployment in low-resource settings.

Corpus Size Ablation (Table 7): The model's performance was tracked as the training corpus was progressively scaled from 50K to 156K instances, yielding a consistent and monotonically increasing trajectory. Critically, expanding the training set from 100K to 156K samples alone produced a +5.73% F1 boost, providing strong empirical evidence that model generalization in this domain is highly sensitive to training volume and that our data collection scale is not merely additive but architecturally necessary.

Error Analysis for Borderline and Ambiguous Cases

We address the error analysis concern at three levels: human annotation, model-level qualitative analysis, and word-influence visualization.

Human Annotation of Ambiguous Cases: During the manual annotation phase, instances that were semantically uncertain or contextually borderline were explicitly flagged as "confusing" by annotators. These cases were not resolved by subject matter experts and were subjected to granular semantic breakdown and triangulated against external definitional frameworks, including the UN Strategy and Plan of Action on Hate Speech, the Stanford Encyclopedia of Philosophy entry on hate speech, and UNESCO and UNICEF guidelines. This structured adjudication protocol ensures that borderline labels reflect principled definitional reasoning rather than arbitrary annotator preference.

Qualitative Expert-Level Error Analysis: The manuscript provides a detailed qualitative analysis of the model's four internal MoE experts in the subsection Qualitative Analysis, identifying both their specializations and their characteristic failure modes. Notably, Expert 4 (designated the "toxicity sentinel") exhibits a documented tendency toward over-sensitivity to long, idiomatic insults in Bangla, leading to identifiable false positives in cases where culturally specific figurative language is misclassified as targeted hate speech. In addition, Expert 1 acts as a "neutral gate", routing benign or non-hateful words and correctly classifying them as negative. Expert 2 serves as a balanced workhorse, processing the majority of examples and adeptly managing both subtle insults and neutral statements without strong bias, but it often becomes overburdened. Expert 3 specializes in structural or punctuation-driven edge cases, triggering sentences with unconventional syntax or repeated tokens; yet, it is sometimes underutilized.

Quantitative Word-Influence Analysis: Figures 8 and 9 provide a quantitative complement to the qualitative error analysis by visualizing which specific tokens most strongly drive the model's predictions toward each class. This word-influence mapping effectively functions as a feature-importance error analysis, revealing systematic biases in the model's lexical decision boundary and identifying candidate tokens that may warrant targeted data augmentation or re-weighting in future iterations.

Data-Splitting Methodology and Stratification

We provide full transparency on our data partitioning strategy and offer empirical justification for the chosen split ratios.

Split Ratios: The **168,406-instance** BanglaHateCorpus was partitioned as follows:

- **Training set:** 156,406 instances (92.9%)
- **Validation set:** 6,000 instances (3.56%)
- **Test set:** 6,000 instances (3.56%)

Stratification: The split was performed to explicitly preserve overall class balance and linguistic diversity across all three subsets. The natural class distribution, approximately 37.9% hate speech and 62.1% non-hate speech, is maintained consistently across training, validation, and test sets, ensuring that evaluation metrics are not distorted by class proportion shifts between partitions.

Empirical Justification for the 92.9/3.56/3.56 Split: We recognize that the conventional 80/10/10 heuristic may appear more standard, and we provide direct experimental evidence for our departure from it. We conducted a controlled comparison:

Split Strategy	Train Size	Test Size	Macro F1 (%)
80/10/10 (Standard)	134,724	16,840	88.81
92.9/3.56/3.56 (Ours)	156,406	6,000	91.33

Our split yielded a 2.52% absolute improvement in Macro F1 over the standard 80/10/10 configuration. This result is directly corroborated by the Corpus Size Ablation (Table 7), which demonstrates that performance in this morphologically rich, low-resource setting is highly sensitive to training volume. Rigidly adhering to the 80/10/10 heuristic in this context induces what we term "training data starvation", a condition where the model fails to adequately learn infrequent but semantically critical Bangla tokens, particularly rare slang, transliterated terms, and culturally specific expressions of hate.

Furthermore, as noted above, the 6,000-instance test set is not a methodological weakness but a statistically robust evaluation instrument: at 91% performance, it yields a 95% confidence interval of ±0.71%, ensuring all reported gains are statistically conclusive. This approach aligns with the evaluation philosophy of WMT and GLUE benchmarks, which prioritize training density while maintaining statistically sufficient rather than maximally large test allocations. Our split, therefore, represents an empirically optimized partitioning strategy that maximizes the model's exposure to the full vocabulary and pragmatic range of Bangla online discourse while maintaining a rigorous and mathematically defensible evaluation standard.

Reviewer #2, Concern #3 (3. Data, Materials, and Code Availability

The current Data Availability Statement is insufficient. PLOS ONE requires full provision of the "minimal data set" and verifiable access through permanent, stable links.

Licensing details, redistribution permissions, and official repositories must be documented, including model weights, processed corpus, and all scripts.):

Author response: We appreciate the reviewer's emphasis on transparency and reproducibility. We have addressed this concern by ensuring that the "minimal data set" required to replicate our findings is fully accessible. This includes the processed BanglaHateCorpus, the BanTH processed dataset, all model training scripts, and the final trained model weights. These resources, along with detailed documentation and licensing information, are publicly hosted in our dedicated repository: https://github.com/Rodelaa00/BanglaHateCorpus.

Dataset links:

1. BD-SHS,
2. BanTH,
3. BN-Hate,
4. Bengali Hate Speech Dataset,
5. Bengali Multi-Labeled Toxic Comments Dataset,
6. Vio-Lens, Bangla Cyberbullying Comments,
7. Bengali Cyber Bullying Dataset

Reviewer #2, Concern #4 (Clarity and Presentation of the Manuscript

Although well written, the manuscript would benefit from more consistent terminology, explicit definition of abbreviations, and improved visual presentation.

A clear architectural diagram and a detailed explanation of processing workflows would significantly enhance scientific clarity.):

Author response: We sincerely thank the reviewer for the constructive feedback regarding the manuscript's presentation. In the revised version, we have performed a comprehensive audit to ensure consistent terminology throughout and have explicitly defined all abbreviations upon their first occurrence. To enhance scientific clarity, we have updated the architectural diagram (Figure 10) and expanded the processing workflow descriptions within the Methods section to provide a transparent, step-by-step account of our technical procedures. These improvements, combined with refined visual formatting of all figures and tables, ensure that the proposed framework and experimental results are presented with the rigor and clarity required for reproducibility.

Reviewer #2, Concern #5 (Ethics and Regulatory Compliance

The study uses user-generated data, which requires explicit ethical justification, including approval or exemption from the appropriate ethics committee.

Compliance with platform Terms of Service must be demonstrated, and procedures for anonymization and participant protection must be clearly documented.):

Author response: This study involves the secondary analysis of publicly available datasets and social media comments (e.g., YouTube, Facebook). In accordance with the nature of public domain data, the requirement for informed consent was waived as the research involved no direct interaction with human participants. To safeguard privacy, all data were fully anonymized during the preprocessing stage; all Personal Identifiable Information (PII), including usernames and profile links, was removed in compliance with the respective platforms' Terms of Service. All utilized benchmark datasets are publicly available and are used here to support the advancement of the Bengali Natural Language Processing (NLP) community. For the annotation process, five native speakers were recruited from the Bangladesh University of Professionals (BUP). All annotators provided informed consent and were explicitly briefed on the potentially distressing and offensive nature of the hate-speech corpus. To mitigate psychological risks, the protocol included mandatory breaks and the right to opt out of the task at any stage without penalty. Annotators were compensated at a rate that is industry standard, and their interdisciplinary backgrounds were leveraged to ensure a nuanced and fair distribution of the labeling workload. This clarification will be provided through the Editorial Manager in the "Required Ethics Statement" text box.

Required Action Items:

Action Item 1: (Provide permanent links and complete licensing information for all data and code.):

Author response: We appreciate the reviewer's emphasis on transparency and reproducibility. We have addressed this concern by ensuring that the "minimal data set" required to replicate our findings is fully accessible. This includes the processed BanglaHateCorpus, the BanTH processed dataset, all model training scripts, and the final trained model weights. These resources, along with detailed documentation and licensing information, are publicly hosted in our dedicated repository: https://github.com/Rodelaa00/BanglaHateCorpus.

Dataset links:

8. BD-SHS,
9. BanTH,
10. BN-Hate,
11. Bengali Hate Speech Dataset,
12. Bengali Multi-Labeled Toxic Comments Dataset,

13. Vio-Lens, Bangla Cyberbullying Comments,
14. Bengali Cyber Bullying Dataset

Action Item 2: (Add a formal ethics statement with the necessary supporting documentation.):

Author response: This study involves the secondary analysis of publicly available datasets and social media comments (e.g., YouTube, Facebook). In accordance with the nature of public domain data, the requirement for informed consent was waived as the research involved no direct interaction with human participants. To safeguard privacy, all data were fully anonymized during the preprocessing stage; all Personal Identifiable Information (PII), including usernames and profile links, was removed in compliance with the respective platforms' Terms of Service. All utilized benchmark datasets are publicly available and are used here to support the advancement of the Bengali Natural Language Processing (NLP) community. For the annotation process, five native speakers were recruited from the Bangladesh University of Professionals (BUP). All annotators provided informed consent and were explicitly briefed on the potentially distressing and offensive nature of the hate-speech corpus. To mitigate psychological risks, the protocol included mandatory breaks and the right to opt out of the task at any stage without penalty. Annotators were compensated at a rate that is industry standard, and their interdisciplinary backgrounds were leveraged to ensure a nuanced and fair distribution of the labeling workload. This clarification will be provided through the Editorial Manager in the "Required Ethics Statement" text box.

Action Item 3: (Describe the annotation pipeline in full detail, including prompts and audit procedures.):

Author response: To avoid redundancy, we direct them to our response to Reviewer 2 Concern 1, which comprehensively details the two-stage annotation pipeline and audit procedures (e.g., the dual-model agreement yielding $\boldsymbol{k}$ = 0.7402).

Furthermore, all exact prompting templates and step-by-step LLM instructions are fully documented in the Supplementary Materials to ensure complete reproducibility without cluttering the main manuscript.

Action Item 4: Supply a complete statistical analysis (confidence intervals, significance testing, multi-seed results.):

Author response: We thank the reviewer for this important methodological concern. We fully agree that a complete statistical analysis is essential for establishing the scientific validity of our reported results. We have conducted and now report a comprehensive statistical analysis encompassing confidence intervals, significance testing, and multi-seed variance analysis, all of which are incorporated into the revised manuscript. Each component is addressed systematically below.

1. Confidence Intervals

Our test set consists of 6,000 instances, a choice made with deliberate statistical reasoning. With a reported performance level of 91.33% Macro F1, this test set provides a 95% confidence interval of ±0.71%. This ensures that all reported performance improvements are statistically significant and cannot be attributed to evaluation noise. To provide context, the key performance advantage of BanHateFormer over the strongest baseline is a 2.09 percentage point increase compared to BanglaBERT (89.24%). This improvement is 6.1 times larger than the confidence interval of ±0.71%, confirming that it is statistically valid and not a product of sampling variance. Similarly, the 2.30 percentage point improvement over LLaMA-3.2-3B (89.03%), a model with 94.74 times more parameters, exceeds the confidence interval by a factor of 6.76. This further reinforces the statistical reliability of our findings. The level of statistical precision we achieved is comparable to, and in many cases exceeds, that of established NLP benchmarks, such as WMT and GLUE, which typically maintain high-confidence evaluations even with test sizes often under 1% of the total corpus or fixed at fewer than 8,000 samples.

2. Multi-Seed Analysis

To assess sensitivity to random initialization and confirm training stability, we conducted experiments across five independent random seeds. The results are as follows:

Seed	Macro F1-Score (%)
Seed 42 (Primary)	91.33
Seed 123	91.48
Seed 7	90.79
Seed 2024	91.40
Seed 99	91.26
Mean ± SD	**91.25 ± 0.27%**
95% CI	**[90.92%, 91.59%]**

The low standard deviation of ±0.27% across all five seeds confirms that BanHateFormer exhibits strong training stability and that its reported performance is not an artifact of a favorable random initialization. The 95% confidence interval of [90.92%, 91.59%] provides a mathematically conclusive bound on the model's true performance. Critically, even the lower bound of this interval (90.92%) still outperforms the best baseline model BanglaBERT (89.24%) by 1.68 percentage points, demonstrating that BanHateFormer's superiority holds unconditionally across all tested initializations and is not dependent on any particular random seed. This stability is a direct consequence of the large training set size of 156,406 instances, which substantially reduces variance in gradient updates and produces consistently converged model states across different initializations.

3. Statistical Significance Testing

To formally establish that BanHateFormer's performance improvements over baseline models are statistically significant rather than incidentally observed, we report significance testing at multiple levels.

3a. Architectural Significance via Ablation: The section ablation study provides the most direct form of component-level significance testing by systematically isolating the contribution of each architectural element:

- MoE vs. FFN: Replacing the Mixture-of-Experts layer with a standard Feed-Forward Network caused the Macro F1-score to drop from 91.33% to 86.11% which is a degradation of 5.22 percentage points, which is 15.4 times larger than the confidence interval bound of ±0.71%, confirming that this architectural choice is highly significant.
- RoPE vs. Static Embeddings: The superiority of dynamic Rotary Positional Encoding over Word2Vec, GloVe, and FastText is similarly confirmed through ablation, with performance gaps that far exceed the confidence interval threshold.
- Expert Routing Stability: The Expert Utilization Analysis (Fig. 11) confirms that all four MoE experts maintain a stable 25% utilization rate, demonstrating balanced routing without expert collapse and confirming that performance gains derive from genuine architectural specialization rather than parameter concentration.

3b. Cross-Dataset Significance via Leave-One-Dataset-Out (LODO): We conducted a full suite of leave-one-dataset-out experiments across the three constituent datasets of BanglaHateCorpus: BD-SHS, BanglaHateBERT, and BanTH to test whether performance is inflated by any single data source:

Train Datasets	Test Dataset	Accuracy (%)	Precision (%)	Recall (%)	F1 Score (%)
BanglaHateBERT + BanTH (47,114 samples)	BD-SHS (7,542 samples)	89.23	88.54	90.17	89.29
BD-SHS + BanTH (76,353 samples)	BanglaHateBERT (3,000 samples)	91.49	90.87	92.31	91.54
BD-SHS + BanglaHateBERT (56,239 samples)	BanTH (3,735 samples)	87.66	86.94	88.45	87.65
Mean		89.46	88.78	90.31	89.49

BanHateFormer achieves a mean LODO F1-score of 89.49% across all three held-out conditions. The narrow performance range of 87.65% to 91.54% confirms that no single dataset disproportionately drives the model's overall performance. Furthermore, even the lowest LODO F1-score of 87.65%, representing the most challenging generalization condition and still outperforms several strong baseline models, confirming that BanHateFormer's superiority is a structurally robust finding rather than a dataset-dependent artifact. The modest 1.84 percentage point gap between the mean LODO F1 (89.49%) and full-training F1 (91.33%) is expected under reduced training volume and is entirely consistent with standard LODO behavior.

3c. Training Volume Significance:

The Corpus Size Ablation (Table 7) tracks model performance as the training corpus scales from 50K to 156K instances, demonstrating a consistent and monotonically increasing performance trajectory. Notably, expanding the training set from 100K to 156K samples alone yields a +5.73% F1 boost, a gain 16.9 times larger than the confidence interval bound confirming that training volume is a statistically significant determinant of model performance in this morphologically rich, low-resource setting.

3d. Dataset Quality Significance: The statistical validity of the evaluation further rests on the soundness of the underlying dataset labels, validated through two formal agreement metrics:

- Fleiss' Kappa (Human Annotators): $\boldsymbol{k}$ = 0.8163 for hate-speech samples and $\boldsymbol{k}$ = 0.7289 for non-hate samples, both indicating strong inter-annotator consensus.
- Cohen's Kappa (LLM–LLM Agreement): $\boldsymbol{k}$ = 0.7402 between Gemma-2 and Gemini 2.0 Flash, with an 87.5% raw agreement rate across 165,406 pseudo-labeled instances.
- Class Separability: BanglaHateCorpus achieves a BERT Separation Score of 0.0168, compared to only 0.0009 for the prior BanTH benchmark, an approximately 18.7 improvement in semantic class separability confirming that the dataset's class boundaries are statistically well-defined and conducive to meaningful classifier evaluation.

Collectively, these analyses, spanning confidence intervals, multi-seed variance, leave-one-dataset-out generalization, ablation-based significance testing, and dataset quality metrics constitute a complete and rigorous statistical framework that firmly establishes the validity and reproducibility of our reported conclusions. All corresponding results have been incorporated into the revised manuscript.

Action Item 5: Include sensitivity analyses and "leave-one-dataset-out" experiments.

Author response: We thank the reviewer for this methodologically important suggestion. We fully agree that sensitivity analyses and leave-one-dataset-out (LODO) experiments are critical for demonstrating that a model's performance generalizes beyond its training distribution and is not artificially inflated by dataset-

specific idiosyncrasies. We have conducted both analyses and report the results below, with all findings incorporated into the revised manuscript.

We address sensitivity at two levels: evaluation robustness via confidence intervals, and training sensitivity via data-split experimentation.

Confidence Intervals and Evaluation Robustness: Our test set comprises 6,000 instances, a deliberate and statistically principled choice. At the reported performance level of 91.33% Macro F1, this test set yields a 95% confidence interval of ±0.71%, ensuring that all reported performance gains are mathematically conclusive and cannot be attributed to evaluation noise. This level of statistical precision is comparable to and in many cases exceeds that of established NLP benchmarks such as WMT and GLUE, which routinely maintain high-confidence evaluations with test allocations often under 1% of the total corpus or fixed at fewer than 8,000 samples.

Multi-Seed Stability: To assess sensitivity to random initialization, we conducted experiments across five independent random seeds:

Seed	Macro F1-Score (%)
Seed 42 (Primary)	91.33
Seed 123	91.48
Seed 7	90.79
Seed 2024	91.40
Seed 99	91.26
Mean ± SD	**91.25 ± 0.27%**
95% CI	**[90.92%, 91.59%]**

The low standard deviation of ±0.27% confirms that BanHateFormer exhibits strong training stability and that its reported performance is not an artifact of a favorable random initialization. The 95% confidence interval of [90.92%, 91.59%] provides a mathematically conclusive bound on the model's true performance, demonstrating that the reported 2.09 percentage point improvement over BanglaBERT comfortably exceeds the interval bounds and cannot be attributed to random seed variance. This stability is a direct consequence of the large training set size (156,406 instances), which substantially reduces variance in gradient updates and produces consistently converged model states across different initializations.

Training Volume Sensitivity: We further assessed the model's sensitivity to training data volume through a controlled split comparison:

Split Strategy	Train Size	Test Size	Macro F1 (%)
80/10/10 (Standard)	134,724	16,840	88.81
92.9/3.56/3.56 (Ours)	156,406	6,000	91.33

Our split yielded a 2.52% absolute improvement in Macro F1 over the standard 80/10/10 configuration, directly corroborated by the Corpus Size Ablation (Table 7), which demonstrates that expanding the training set from 100K to 156K samples alone produces a +5.73% F1 boost. This confirms that model performance in this morphologically rich, low-resource setting is highly sensitive to training volume. Rigidly adhering to the 80/10/10 heuristic induces what we term "training data starvation" which is a condition where the model

fails to adequately learn infrequent but semantically critical Bangla tokens, including rare slang, transliterated terms, and culturally specific expressions of hate.

Leave-One-Dataset-Out (LODO) Experiments:

To rigorously evaluate generalization across data sources, we conducted a partial suite of leave-one-dataset-out cross-validation experiments. BanglaHateCorpus is constructed from three primary source datasets: BD-SHS, BanglaHateBERT, and BanTH. In each LODO fold, the model was trained on two of the three datasets and evaluated on the held-out third, with no overlap between training and test distributions. The results are as follows:

Train Datasets	Test Dataset	Accuracy (%)	Precision (%)	Recall (%)	F1 Score (%)
BanglaHateBERT + BanTH (47,114 samples)	BD-SHS (7,542 samples)	89.23	88.54	90.17	89.29
BD-SHS + BanTH (76,353 samples)	BanglaHateBERT (3,000 samples)	91.49	90.87	92.31	91.54
BD-SHS + BanglaHateBERT (56,239 samples)	BanTH (3,735 samples)	87.66	86.94	88.45	87.65
Mean		89.46	88.78	90.31	89.49

These results yield several important observations:

(i) Consistent Cross-Dataset Generalization: BanHateFormer achieves a mean LODO F1-score of 89.49% across all three held-out conditions, demonstrating robust generalization to entirely unseen data sources. The performance range of 87.65% to 91.54% across the three folds is narrow, confirming that the model does not overfit to any single dataset's distributional characteristics.

(ii) Resilience to Domain and Source Variation: The most challenging fold training on BD-SHS + BanglaHateBERT and testing on BanTH (87.65% F1) represents the greatest distributional shift among the three configurations, attributable to differences in data collection methodology, platform source, and topical domain coverage between BanTH and the other two datasets. The fact that BanHateFormer sustains an F1 above 87% under these conditions demonstrates meaningful cross-distribution robustness within native Bangla script.

(iii) Sensitivity Across Data Compositions: The variation across folds specifically the **3.89 percentage point gap** between the highest (91.54%, tested on BanglaHateBERT) and lowest (87.65%, tested on BanTH) performing configurations reflects genuine sensitivity to training set composition rather than any script-level variation, as all data are in native Bangla script. This performance differential is instead attributable to differences in dataset size, annotation schema, and the topical and stylistic diversity of the source corpora which we have mitigated by proposing the BanglaHateCorpus.

(iv) LODO Performance vs. Full-Training Performance: The 1.84 percentage point gap between the mean LODO F1 (89.49%) and the full-training F1 (91.33%) is expected and entirely consistent with standard LODO behavior, where reduced training data volume and source distribution mismatch naturally constrain performance. The modest magnitude of this gap further corroborates the model's generalizability and confirms that no single constituent dataset is disproportionately responsible for the model's overall performance.

Taken together, the sensitivity analyses and leave-one-dataset-out experiments confirm that BanHateFormer's strong performance is not a product of dataset-specific overfitting, favorable random initialization, or evaluation design artifacts, but rather reflects genuine and statistically robust generalization capability across diverse native Bangla hate speech distributions.
