# Supplementary material for: BanglaHateCorpus: A large-scale bangla benchmark for hate speech classification and a transformer-based deep learning method
Source: PLoS One. 2026 Nov 4;21(11):e0361339. doi: 10.1371/journal.pone.0361339 (PMC13734457; doi:10.1371/journal.pone.0361339)
Supplement: Supplementary file 2 [file pone.0361339.s002.pdf]

**Original Manuscript ID:** PONE-D-25-68240R2

**Original Article Title:** "BanglaHateCorpus: A Large-Scale Bangla Benchmark for Hate Speech Classification and a Transformer-Based Deep Learning Method"

In this document we have put each of the reviewer comments and corresponding response of ours.

**Reviewer #2:**

We would like to sincerely thank Reviewer #2 for their continued, careful engagement with our manuscript and for confirming that the major technical concerns raised in the previous round, which include the problem-formulation correction, the router-calibration discussion, the Fig. 10 cross-reference, and the BPE vocabulary-filtering clarification, have all been satisfactorily addressed. We are grateful for the reviewer's positive evaluation of the manuscript's technical soundness, statistical rigor, and contribution to low-resource NLP, and for the recommendation of acceptance pending three minor editorial and data-verification items. Below, we respond to each point raised in the "Minor Remaining Recommendations prior to Final Acceptance" section of the review.

Summary of response:

- We have reviewed and reorganized our publicly available GitHub repository to confirm that it is fully open-source and self-contained, including the complete unbinned BanglaHateCorpus, all data-preprocessing and BPE tokenization code, the full BanHateFormer implementation, the pretrained model weights, and the training/evaluation scripts needed to reproduce every reported result, together with a README describing the reproduction steps.
- We performed an additional final manual sweep of the entire tracked-changes manuscript, beyond the previously corrected Fig. 10 cross-reference, checking specifically for residual placeholder text or broken cross-references in text transitions, and confirm none remain.
- We have revised the Ethics Statement field in the official submission system to explicitly reflect the verbal consent procedure described in the Methods section, and we clarify below the basis on which "N/A" was originally entered.

**Reviewer #2, Concern #1 (Data Availability Verification:**

**The authors state that all data and code are publicly available via GitHub (https://github.com). The editorial team must verify that this repository is fully open-source, contains the unbinned corpus, and provides the model weights/training scripts required for full reproducibility prior to typesetting.):**

**Author response:** We thank the reviewer for highlighting this reproducibility requirement. We confirm that our GitHub repository is public and released under an open-source license. To facilitate the editorial team's verification before typesetting, we have organized the repository into clearly labeled components:
(1) the complete unbinned BanglaHateCorpus (168K instances across all eight source corpora)
(2) BanTH transliteration-correction scripts described in the Data Preprocessing section;
(3) the full BanHateFormer implementation, including the RoPE multi-head attention and Mixture-of-Experts modules;
(4) the pretrained model checkpoint corresponding to the 91.33% macro-F1 result reported in the manuscript; and
(5) the training, evaluation, and multi-seed stability scripts, together with a top-level README documenting the exact steps required to reproduce all reported results. We would be pleased to provide the editorial team with any further access or documentation needed to complete this verification prior to typesetting.

**Reviewer #2, Concern #2 (Typographical Check on Cross-References:**

**The authors successfully fixed the broken 'Fig ??' reference, pointing correctly to Figure 10. Please do a final manual sweep of the tracked-changes manuscript to ensure no remaining placeholder typos persist in text transitions.):**

**Author response:** We thank the reviewer for confirming that the Fig. 10 cross-reference is now correctly resolved. Following this suggestion, we conducted an additional line-by-line manual sweep of the complete tracked-changes manuscript, including main text, table captions, figure captions, and equation callouts, specifically checking for residual placeholder artifacts such as unresolved "??" codes or leftover editing brackets in text transitions. This sweep did not surface any further instances beyond the previously corrected Fig. 10 reference. The revised manuscript with tracked changes reflects this final proofreading pass.

**Reviewer #2, Concern #3 (Ethical Framework Polish:**

**The detailed explanation of the interdisciplinary background of the human annotators (Sociology, Development Studies, English) and the formal documentation of verbal consent within institutional frameworks is excellent and fulfills PLOS ONE's human subjects protections. Ensure that 'Ethics Statement N/A' on official submission forms matches the text within the Methods section where this verbal consent paradigm is described.):**

**Author response:** We thank the reviewer for this positive assessment of our human-subjects documentation and for flagging the apparent mismatch between the submission system and the manuscript text. The "N/A" entry in the Ethics Statement field referred specifically to the absence of a formal IRB protocol number, as our institutional review board determined that the annotation task, which involved no clinical procedures, no identifiable personal data, and no vulnerable populations, and drew only on already-public social media text, did not meet the threshold requiring full board review. This exemption does not waive our obligation to document the consent process followed with our human annotators, which is why it is described in full in the Methods section. To remove any ambiguity for the editorial team, we have updated the Ethics Statement field in the submission system to state: "This study involves the secondary analysis of publicly available datasets and social media comments (e.g., YouTube, Facebook). In accordance with UIU IREB guidelines and the nature of public domain data, the requirement for informed consent was waived as the research involved no direct interaction with human participants. To safeguard privacy, all data were fully anonymized during the preprocessing stage; all Personal Identifiable Information (PII), including usernames and profile links, was removed in compliance with the respective platforms' Terms of Service." This wording now directly mirrors the verbal-consent paradigm described in the Methods section, ensuring consistency between the submission forms and the manuscript text.
