# Supplementary material for: BanglaHateCorpus: A large-scale bangla benchmark for hate speech classification and a transformer-based deep learning method
Source: PLoS One. 2026 Nov 4;21(11):e0361339. doi: 10.1371/journal.pone.0361339 (PMC13734457; doi:10.1371/journal.pone.0361339)
Supplement: Peer review history

# Peer review: Decision Letter 0

**DOI:** 10.1371/journal.pone.0361339.r001

**Authors:** Ciro Clemente De Falco

27 Mar 2026

BanglaHateCorpus: A Large-Scale Bangla Benchmark for Hate Speech Classification and a Transformer-Based Deep Learning Method

PLOS One

Dear Dr. Rodela,

Thank you for submitting your manuscript to PLOS ONE. After careful consideration, we feel that it has merit but does not fully meet PLOS ONE’s publication criteria as it currently stands. Therefore, we invite you to submit a revised version of the manuscript that addresses the points raised during the review process.

Please submit your revised manuscript by May 10 2026 11:59PM. If you will need more time than this to complete your revisions, please reply to this message or contact the journal office at plosone@plos.org. When you're ready to submit your revision, log on to https://www.editorialmanager.com/pone/ and select the 'Submissions Needing Revision' folder to locate your manuscript file.

Please include the following items when submitting your revised manuscript:

- A letter that responds to each point raised by the academic editor and reviewer(s). You should upload this letter as a separate file labeled 'Response to Reviewers'.
- A marked-up copy of your manuscript that highlights changes made to the original version. You should upload this as a separate file labeled 'Revised Manuscript with Track Changes'.
- An unmarked version of your revised paper without tracked changes. You should upload this as a separate file labeled 'Manuscript'.

If you would like to make changes to your financial disclosure, please include your updated statement in your cover letter. Guidelines for resubmitting your figure files are available below the reviewer comments at the end of this letter.

If applicable, we recommend that you deposit your laboratory protocols in protocols.io to enhance the reproducibility of your results. Protocols.io assigns your protocol its own identifier (DOI) so that it can be cited independently in the future. For instructions see: https://journals.plos.org/plosone/s/submission-guidelines#loc-laboratory-protocols. Additionally, PLOS ONE offers an option for publishing peer-reviewed Lab Protocol articles, which describe protocols hosted on protocols.io. Read more information on sharing protocols at https://plos.org/protocols?utm_medium=editorial-email&utm_source=authorletters&utm_campaign=protocols.

As the corresponding author, your ORCID iD is verified in the submission system and will appear in the published article. PLOS supports the use of ORCID, and we encourage all coauthors to register for an ORCID iD and use it as well. Please encourage your coauthors to verify their ORCID iD within the submission system before final acceptance, as unverified ORCID iDs will not appear in the published article. *Only*  the individual author can complete the verification step; PLOS staff *cannot*  verify ORCID iDs on behalf of authors.

We look forward to receiving your revised manuscript.

Kind regards,

Ciro Clemente De Falco

Academic Editor

PLOS One

Journal Requirements:

When submitting your revision, we need you to address these additional requirements.

1.Please ensure that your manuscript meets PLOS ONE's style requirements, including those for file naming. The PLOS ONE style templates can be found at

https://journals.plos.org/plosone/s/file?id=wjVg/PLOSOne_formatting_sample_main_body.pdf and

https://journals.plos.org/plosone/s/file?id=ba62/PLOSOne_formatting_sample_title_authors_affiliations.pdf

2. Please provide additional details regarding participant consent. In the ethics statement in the Methods and online submission information, please ensure that you have specified (1) whether consent was informed and (2) what type you obtained (for instance, written or verbal, and if verbal, how it was documented and witnessed). If your study included minors, state whether you obtained consent from parents or guardians. If the need for consent was waived by the ethics committee, please include this information.

If you are reporting a retrospective study of medical records or archived samples, please ensure that you have discussed whether all data were fully anonymized before you accessed them and/or whether the IRB or ethics committee waived the requirement for informed consent. If patients provided informed written consent to have data from their medical records used in research, please include this information.

3. In your Methods section, please include additional information about your dataset and ensure that you have included a statement specifying whether the collection and analysis method complied with the terms and conditions for the source of the data.

4. Please note that PLOS One has specific guidelines on code sharing for submissions in which author-generated code underpins the findings in the manuscript. In these cases, we expect all author-generated code to be made available without restrictions upon publication of the work. Please review our guidelines at https://journals.plos.org/plosone/s/materials-and-software-sharing#loc-sharing-code and ensure that your code is shared in a way that follows best practice and facilitates reproducibility and reuse.

5. Please include additional information in your revisions on who the annotators were, how they were recruited, and whether any ethical considerations were obtained.

6. Please note that funding information should not appear in any section or other areas of your manuscript. We will only publish funding information present in the Funding Statement section of the online submission form. Please remove any funding-related text from the manuscript.

7. Thank you for stating the following financial disclosure:

“This research received funding from the ICT division of the Government of

the People’s Republic of Bangladesh for the 2023–24 financial year (tracking

no:25FS18283).”

Please state what role the funders took in the study.  If the funders had no role, please state: "The funders had no role in study design, data collection and analysis, decision to publish, or preparation of the manuscript."

If this statement is not correct you must amend it as needed.

Please include this amended Role of Funder statement in your cover letter; we will change the online submission form on your behalf.

8. Please expand the acronym “ICT” (as indicated in your financial disclosure) so that it states the name of your funders in full.

This information should be included in your cover letter; we will change the online submission form on your behalf.

9. When completing the data availability statement of the submission form, you indicated that you will make your data available on acceptance. We strongly recommend all authors decide on a data sharing plan before acceptance, as the process can be lengthy and hold up publication timelines. Please note that, though access restrictions are acceptable now, your entire data will need to be made freely accessible if your manuscript is accepted for publication. This policy applies to all data except where public deposition would breach compliance with the protocol approved by your research ethics board. If you are unable to adhere to our open data policy, please kindly revise your statement to explain your reasoning and we will seek the editor's input on an exemption. Please be assured that, once you have provided your new statement, the assessment of your exemption will not hold up the peer review process.

10. We note that Figure 4 in your submission contain copyrighted images. All PLOS content is published under the Creative Commons Attribution License (CC BY 4.0), which means that the manuscript, images, and Supporting Information files will be freely available online, and any third party is permitted to access, download, copy, distribute, and use these materials in any way, even commercially, with proper attribution. For more information, see our copyright guidelines: http://journals.plos.org/plosone/s/licenses-and-copyright.

We require you to either (1) present written permission from the copyright holder to publish these figures specifically under the CC BY 4.0 license, or (2) remove the figures from your submission:

1. You may seek permission from the original copyright holder of Figure(s) [#] to publish the content specifically under the CC BY 4.0 license.

We recommend that you contact the original copyright holder with the Content Permission Form (http://journals.plos.org/plosone/s/file?id=7c09/content-permission-form.pdf) and the following text:

“I request permission for the open-access journal PLOS ONE to publish XXX under the Creative Commons Attribution License (CCAL) CC BY 4.0 (http://creativecommons.org/licenses/by/4.0/). Please be aware that this license allows unrestricted use and distribution, even commercially, by third parties. Please reply and provide explicit written permission to publish XXX under a CC BY license and complete the attached form.”

Please upload the completed Content Permission Form or other proof of granted permissions as an "Other" file with your submission.

In the figure caption of the copyrighted figure, please include the following text: “Reprinted from [ref] under a CC BY license, with permission from [name of publisher], original copyright [original copyright year].”

2. If you are unable to obtain permission from the original copyright holder to publish these figures under the CC BY 4.0 license or if the copyright holder’s requirements are incompatible with the CC BY 4.0 license, please either i) remove the figure or ii) supply a replacement figure that complies with the CC BY 4.0 license. Please check copyright information on all replacement figures and update the figure caption with source information. If applicable, please specify in the figure caption text when a figure is similar but not identical to the original image and is therefore for illustrative purposes only.

11. Please ensure that you refer to Figures 8-10 in your text as, if accepted, production will need this reference to link the reader to the figure

12. If the reviewer comments include a recommendation to cite specific previously published works, please review and evaluate these publications to determine whether they are relevant and should be cited. There is no requirement to cite these works unless the editor has indicated otherwise.

Additional Editor Comments:

Thank you for submitting your manuscript.

The reviewers have appreciated the quality and relevance of your work. However, they have also identified several substantial issues that need to be addressed before the manuscript can be considered for publication.

I therefore invite you to revise your manuscript in light of the reviewers’ comments and to submit a thoroughly revised version, together with a detailed point-by-point response.

I look forward to receiving your revision.

Kind regards,

[Note: HTML markup is below. Please do not edit.]

Reviewers' comments:

Reviewer's Responses to Questions

**Comments to the Author**

1. Is the manuscript technically sound, and do the data support the conclusions?

Reviewer #1: Yes

Reviewer #2: Partly

**********

2. Has the statistical analysis been performed appropriately and rigorously?

Reviewer #1: N/A

Reviewer #2: Yes

**********

3. Have the authors made all data underlying the findings in their manuscript fully available?

The PLOS Data policy

Reviewer #1: Yes

Reviewer #2: Yes

**********

4. Is the manuscript presented in an intelligible fashion and written in standard English?

Reviewer #1: Yes

Reviewer #2: Yes

**********

Reviewer #1: This study has created a large corpus based on semi-supervised learning concept of data annotations using human experts as well as LLMs based learning. Overall good work, as latest BERT -based transformers are explored. Write up is satisfactory, research methodology is also process. Minor observation have been shared for improvements.

1

How can 94.74x can be parameters. Is there some typo in the claimed contribution in introduction “state-of-the-art performance while using approximately 94.74× fewer parameters”?

2

Figure sized and content within figure is too small. Fig should be englarged for better visibility and quality should be improved. Fig 10 size and quality can be used for other figures too.

3

Why Jaccard similarity is computed. Why not only metrics such as Dice Coefficient and Cosine Similarity are also considered for application?

4

Details of human annotators are not given. How they were selected? Were they qualified and were having expertise to carry out the task. Has Kappa Statistics been applied to check whether they were correct to annotate the data?

5

URLs like link of one source of hugging face is given. IT is recommended that last date retrieved should be mentioned. As these URLS are likely to get obsolete or change

6

It is mentioned that “influence is calculated as (word coefficient/sum of positive coefficients) × 100.”. What is the logic of using it? An working example with assumed values be used for computation process and to show the steps of computation for better understanding. Also if this is processed method by the author or they have taken from any other study, reference should be provided.

7

Such error shows that the proofread is requird, “The entire intrusion classification method is illustrated in Fig ??.”

8

Why test and validation division is merely less than 4 %.?

9

There is no related work but background is provided. Need to discuss the review of latest publications from years 2024-6 in this section to highlight latest work in the corpus and sentiment analysis.

doi: 10.1145/3773075

doi: 10.1109/TCE.2026.3652186

doi: https://doi.org/10.1016/j.knosys.2025.115123

10

It should be checked again that no Bangla content is in the manuscript without English translation as otherwise, it will be helpful for understanding of the readers.

Reviewer #2: Executive Summary

This report provides an in‑depth evaluation of the manuscript submitted to PLOS ONE. The main contribution lies in the creation of a large‑scale unified corpus and the development of a highly efficient monolingual deep‑learning model for Bangla hate‑speech classification. After a thorough assessment aligned with PLOS ONE’s editorial and scientific criteria, the recommendation is Major Revision, given the clear need for methodological strengthening, enhanced transparency, improved reproducibility, ethical adequacy, and strict compliance with the journal’s data‑availability policy.

1. Technical Soundness and Validity of the Conclusions

The manuscript presents a solid approach and a well‑articulated motivation. The unification of eight datasets is valuable, but it requires additional transparency, particularly regarding the integration of toxicity and cyberbullying data when the stated objective is hate‑speech classification.

The hybrid human–LLM annotation pipeline is innovative; however, it requires reproducible detail, including exact model versions, parameters, prompts, and agreement criteria.

The proposed BanHateFormer model appears promising in terms of efficiency, yet complete quantitative evidence comparing it with baseline models—along with robust statistical testing—is currently lacking.

2. Statistical Analysis and Quantitative Rigor

The evaluation must include confidence intervals, statistical significance testing, and multi‑seed analysis.

Ablation studies for each architectural component are necessary, as well as a detailed error analysis, particularly for borderline or ambiguous cases.

The data‑splitting methodology must be fully clarified, including any stratification techniques used.

3. Data, Materials, and Code Availability

The current Data Availability Statement is insufficient. PLOS ONE requires full provision of the “minimal data set” and verifiable access through permanent, stable links.

Licensing details, redistribution permissions, and official repositories must be documented, including model weights, processed corpus, and all scripts.

4. Clarity and Presentation of the Manuscript

Although well written, the manuscript would benefit from more consistent terminology, explicit definition of abbreviations, and improved visual presentation.

A clear architectural diagram and a detailed explanation of processing workflows would significantly enhance scientific clarity.

5. Ethics and Regulatory Compliance

The study uses user‑generated data, which requires explicit ethical justification, including approval or exemption from the appropriate ethics committee.

Compliance with platform Terms of Service must be demonstrated, and procedures for anonymization and participant protection must be clearly documented.

Required Action Items

Provide permanent links and complete licensing information for all data and code.

Add a formal ethics statement with the necessary supporting documentation.

Describe the annotation pipeline in full detail, including prompts and audit procedures.

Supply a complete statistical analysis (confidence intervals, significance testing, multi‑seed results).

Include sensitivity analyses and “leave‑one‑dataset‑out” experiments.

Proposed Editorial Decision

Major Revision

**********

what does this mean?). If published, this will include your full peer review and any attached files.

If you choose “no”, your identity will remain anonymous but your review may still be made public.

**Do you want your identity to be public for this peer review?**  For information about this choice, including consent withdrawal, please see our Privacy Policy

Reviewer #1: No

Reviewer #2: No

**********

[NOTE: If reviewer comments were submitted as an attachment file, they will be attached to this email and accessible via the submission site. Please log into your account, locate the manuscript record, and check for the action link "View Attachments". If this link does not appear, there are no attachment files.]

To ensure your figures meet our technical requirements, please review our figure guidelines: https://journals.plos.org/plosone/s/figures

You may also use PLOS’s free figure tool, NAAS, to help you prepare publication quality figures: https://journals.plos.org/plosone/s/figures#loc-tools-for-figure-preparation.

NAAS will assess whether your figures meet our technical requirements by comparing each figure against our figure specifications.

# Author comment: Author response to Decision Letter 1

**DOI:** 10.1371/journal.pone.0361339.r002

8 Jun 2026

Response to Reviewers file is already attached to this submission.

Attachment Submitted filename: Response to Reviewers - PONE-D-25-68240_R1.pdf

# Peer review: Decision Letter 1

**DOI:** 10.1371/journal.pone.0361339.r003

**Authors:** Ciro Clemente De Falco

2 Jul 2026

Dear Dr. Rodela,

Thank you for submitting your manuscript to PLOS ONE. After careful consideration, we feel that it has merit but does not fully meet PLOS ONE’s publication criteria as it currently stands. Therefore, we invite you to submit a revised version of the manuscript that addresses the points raised during the review process.

Please submit your revised manuscript by Aug 16 2026 11:59PM. If you will need more time than this to complete your revisions, please reply to this message or contact the journal office at plosone@plos.org. When you're ready to submit your revision, log on to https://www.editorialmanager.com/pone/ and select the 'Submissions Needing Revision' folder to locate your manuscript file.

- A letter that responds to each point raised by the academic editor and reviewer(s). You should upload this letter as a separate file labeled 'Response to Reviewers'.
- A marked-up copy of your manuscript that highlights changes made to the original version. You should upload this as a separate file labeled 'Revised Manuscript with Track Changes'.
- An unmarked version of your revised paper without tracked changes. You should upload this as a separate file labeled 'Manuscript'.

If you would like to make changes to your financial disclosure, please include your updated statement in your cover letter. Guidelines for resubmitting your figure files are available below the reviewer comments at the end of this letter.

If applicable, we recommend that you deposit your laboratory protocols in protocols.io to enhance the reproducibility of your results. Protocols.io assigns your protocol its own identifier (DOI) so that it can be cited independently in the future. For instructions see: https://journals.plos.org/plosone/s/submission-guidelines#loc-laboratory-protocols. Additionally, PLOS ONE offers an option for publishing peer-reviewed Lab Protocol articles, which describe protocols hosted on protocols.io. Read more information on sharing protocols at https://plos.org/protocols?utm_medium=editorial-email&utm_source=authorletters&utm_campaign=protocols.

As the corresponding author, your ORCID iD is verified in the submission system and will appear in the published article. PLOS supports the use of ORCID, and we encourage all coauthors to register for an ORCID iD and use it as well. Please encourage your coauthors to verify their ORCID iD within the submission system before final acceptance, as unverified ORCID iDs will not appear in the published article. *Only*  the individual author can complete the verification step; PLOS staff *cannot*  verify ORCID iDs on behalf of authors.

We look forward to receiving your revised manuscript.

Kind regards,

Ciro Clemente De Falco

Academic Editor

PLOS One

**Journal Requirements:**

1. If the reviewer comments include a recommendation to cite specific previously published works, please review and evaluate these publications to determine whether they are relevant and should be cited. There is no requirement to cite these works unless the editor has indicated otherwise.

2. Please review your reference list to ensure that it is complete and correct. If you have cited papers that have been retracted, please include the rationale for doing so in the manuscript text, or remove these references and replace them with relevant current references. Any changes to the reference list should be mentioned in the rebuttal letter that accompanies your revised manuscript. If you need to cite a retracted article, indicate the article’s retracted status in the References list and also include a citation and full reference for the retraction notice.

[Note: HTML markup is below. Please do not edit.]

Reviewers' comments:

Reviewer's Responses to Questions

**Comments to the Author**

Reviewer #1: All comments have been addressed

Reviewer #2: All comments have been addressed

**********

2. Is the manuscript technically sound, and do the data support the conclusions?

Reviewer #1: Yes

Reviewer #2: Yes

**********

3. Has the statistical analysis been performed appropriately and rigorously?

Reviewer #1: Yes

Reviewer #2: Yes

**********

4. Have the authors made all data underlying the findings in their manuscript fully available?

The PLOS Data policy

Reviewer #1: Yes

Reviewer #2: Yes

**********

5. Is the manuscript presented in an intelligible fashion and written in standard English?

Reviewer #1: Yes

Reviewer #2: Yes

**********

**Reviewer #1:**  The authors have addressed all my comments properly. The manuscript is much improved to be recommended for acceptance.

**Reviewer #2:**  Manuscript Number: PONE-D-25-68240R1

Title: BanglaHateCorpus: A Large-Scale Bangla Benchmark for Hate Speech Classification and a Transformer-Based Deep Learning Method

Reviewer Recommendation: Minor Revision

________________________________________

Reviewer Comments to the Author:

1. General Evaluation and Contributions

The manuscript addresses a critical bottleneck in South Asian NLP: the lack of a standardized, large-scale, and quality-assured dataset for low-resource languages like Bangla, alongside the computational inefficiency of contemporary deployment models.

The authors’ contributions are highly commendable:

-BanglaHateCorpus: Merging and cross-verifying a massive 168K-sample collection sets a solid benchmark for text-moderation tasks.

• Hybrid Annotation Pipeline: The two-stage data-annotation process—pairing UN/UNESCO human frameworks with LLM-as-a-judge capabilities (Gemini 2.0 Flash and Gemma-2)—is rigorous and mathematically validated by strong Fleiss’ kappa (\(0.8163\)) and Cohen’s kappa (\(0.7402\)) metrics (pp. 19-20).

-BanHateFormer Architecture: The model shows outstanding parameter efficiency (31.68M parameters), outperforming or matching deep learning networks that are up to two orders of magnitude larger (e.g., Gemma-2-2B, LLaMA-3.2-3B) (pp. 28-29). This represents a breakthrough in sustainable AI deployments for resource-constrained architectures.

The manuscript has noticeably improved from its initial draft. However, a severe technical inconsistency in the problem formulation and method section must be addressed before final publication.

________________________________________

2. Major Technical Concern (Required Correction)

-Methodological Inconsistency (Section: Proposed Methodology, Page 22):

In lines 528–538, the authors state: "...Passed through an encoder \(\tau(\cdot)\) to transform the ordinal & nominal text to numerical data... then fed into a tree-based model \(\mu(\cdot)\) that is tweaked for the intrusion classification task. Mathematically... \(\hat{y} = \mu(\omega(\tau([x_1, x_2, ..., x_n])))\)." (p. 22)Critique: This paragraph completely contradicts the core architecture of the paper. BanHateFormer is inherently a Vaswani-style Transformer encoder leveraging Rotary Position Embeddings (RoPE), SwiGLU activations, and a MoE (Mixture of Experts) layer (pp. 24, 31). It is not a "tree-based model," nor is this a network for "intrusion classification" (it is hate-speech detection). This appears to be a severe copy-paste or drafting error from a different manuscript. The problem formulation must be entirely rewritten to mathematically define token embedders, multi-head attention blocks, and gated routing mechanisms rather than tree split matrices.

________________________________________

3. Minor Specific Critiques and Suggestions

-Routing Calibration & Expert Limitations (Page 30):

The qualitative analysis reveals fascinating insights: Expert 2 becomes chronically "overburdened," and Expert 4 ("toxicity sentinel") acts as a primary vector for false positives due to over-sensitivity toward long, idiomatic insults (p. 30).

-Request: To complement this transparency, please add 2–3 sentences in the Discussion or Future Work section detailing how you might tune the auxiliary router z-loss or implement dynamic token-capacity adjustments to alleviate Expert 2's load and scale down Expert 4's hyper-sensitivity (p. 25).

-Figure Reference Typo (Page 22):

Line 537 states: "The entire intrusion classification method is illustrated in Fig ??" (p. 22). Please fix this broken cross-reference link and point it directly to the correct workflow architecture block diagram (Fig 10) (p. 24).

-Vocabulary Separation Insights (Page 22 / Figures 8 & 9):

The word coefficient charts present a highly clear lexical separation between the classes (pp. 22-23). However, some offensive terms routed by the "toxicity sentinel" may change meaning based on code-mixed conditions. Authors are encouraged to briefly state whether emojis or alphanumeric character substitutions affected the Byte-Pair-Encoding (BPE) subword parsing stage (p. 23).

________________________________________

Conclusion

This paper offers a high-utility baseline that pushes Bangla NLP forward. However, due to the severe discrepancy between the text implementation description and the mathematical modeling formulas, the manuscript is currently not in a publishable state. Once this technical drafting error in the methodology is completely resolved and strictly aligned with the actual deep-learning transformer implementation, the paper would then meet the necessary foundational requirements for rigorous peer evaluation and future publication consideration in PLOS ONE.

**********

what does this mean?). If published, this will include your full peer review and any attached files.

If you choose “no”, your identity will remain anonymous but your review may still be made public.

**Do you want your identity to be public for this peer review?**  For information about this choice, including consent withdrawal, please see our Privacy Policy

Reviewer #1: No

Reviewer #2: No

**********

[NOTE: If reviewer comments were submitted as an attachment file, they will be attached to this email and accessible via the submission site. Please log into your account, locate the manuscript record, and check for the action link "View Attachments". If this link does not appear, there are no attachment files.]

To ensure your figures meet our technical requirements, please review our figure guidelines: https://journals.plos.org/plosone/s/figures

You may also use PLOS’s free figure tool, NAAS, to help you prepare publication quality figures: https://journals.plos.org/plosone/s/figures#loc-tools-for-figure-preparation.

NAAS will assess whether your figures meet our technical requirements by comparing each figure against our figure specifications.

# Author comment: Author response to Decision Letter 2

**DOI:** 10.1371/journal.pone.0361339.r004

21 Jul 2026

Original Manuscript ID: PONE-D-25-68240R1

Original Article Title: “BanglaHateCorpus: A Large-Scale Bangla Benchmark for Hate Speech Classification and a Transformer-Based Deep Learning Method”

In this document we have put each of the reviewer comments and corresponding response of ours.

Reviewer #2:

We sincerely thank Reviewer #2 for the meticulous and rigorous technical scrutiny of our manuscript, and for identifying an architectural inconsistency in our problem formulation that had persisted from an earlier draft. We are especially encouraged by the reviewer's careful cross-referencing of our equations, figures, and qualitative analysis, which reflects a deep engagement with both the mathematical formulation of BanHateFormer and the behavior of its Mixture-of-Experts components. In response to the observations raised, we have carefully addressed each concern and believe the revisions meaningfully strengthen the technical accuracy, transparency, and reproducibility of the manuscript. A point-by-point summary of all changes is provided below.

Summary of response:

We have addressed the previously flagged methodological inconsistency in the Proposed Method section. The earlier references to a "tree-based model" and "intrusion classification" were artifacts from an unrelated manuscript and were corrected in our first revision (R1). In the updated text, Equation 3 now clearly defines τ(·) as a pre-trained Byte-Pair Encoding tokenizer and µ(·) as the Bangla hate-speech classification model. This is consistent with the Vaswani-style Transformer encoder, which includes RoPE (Rotary Position Embeddings), multi-head self-attention, and the Mixture-of-Experts layer, as detailed in the BanHateFormer subsection and illustrated in Figure 10.

We have addressed the concerns regarding router calibration and expert imbalance that emerged from our qualitative analysis. In the Conclusion and Future Work section, we propose adding three new sentences that suggest increasing the weight of the auxiliary router z-loss (λz) to mitigate Expert 4's excessive sensitivity to long, idiomatic insults. Additionally, we recommend replacing the soft load-balancing term with a hard, dynamic per-expert token-capacity limit, which draws on the capacity-factor mechanisms of the Switch Transformer [69] and ST-MoE [68]. This adjustment aims to redistribute Expert 2's workload towards the underutilized Expert 3.

We corrected the broken "Fig ??" cross-reference: this was likewise resolved during our first revision, with the corresponding sentence now correctly pointing to Fig. 10, the block diagram illustrating tokenization, embedding, RMSNorm, rotary positional encoding, multi-head attention, and the Mixture-of-Experts pipeline.

We investigated whether emojis or alphanumeric substitutions have an impact on Byte-Pair-Encoding subword parsing. Our deterministic character-level filter (Data Preprocessing) limits each sentence in the corpus to an 89-symbol Bangla character set, which includes 72 graphemes, 16 punctuation marks, and the space character. As a result, emojis, digits, and Latin-script substitutions do not reach the tokenizer τ(·). Additionally, we included an explicit statement documenting this exclusion and noted that modeling at the emoji level is a potential direction for future work.

Reviewer #2, Concern #2 (Major Technical Concern (Required Correction)

-Methodological Inconsistency (Section: Proposed Methodology, Page 22):

In lines 528–538, the authors state: "...Passed through an encoder \(\tau(\cdot)\) to transform the ordinal & nominal text to numerical data... then fed into a tree-based model \(\mu(\cdot)\) that is tweaked for the intrusion classification task. Mathematically... \(\hat{y} = \mu(\omega(\tau([x_1, x_2, ..., x_n])))\)." (p. 22)Critique: This paragraph completely contradicts the core architecture of the paper. BanHateFormer is inherently a Vaswani-style Transformer encoder leveraging Rotary Position Embeddings (RoPE), SwiGLU activations, and a MoE (Mixture of Experts) layer (pp. 24, 31). It is not a "tree-based model," nor is this a network for "intrusion classification" (it is hate-speech detection). This appears to be a severe copy-paste or drafting error from a different manuscript. The problem formulation must be entirely rewritten to mathematically define token embedders, multi-head attention blocks, and gated routing mechanisms rather than tree split matrices.):

Author response: We appreciate the reviewer’s careful examination of our manuscript. We would like to clarify that this particular issue was addressed and corrected in our first revision (R1). This correction was documented and highlighted in our response letter for R1. In the current manuscript, the Proposed Method section (Eq. 3) states: ŷ = µ(ω(τ([x₁, x₂, ..., xₙ]))), where τ(·) is explicitly defined as a pre-trained Byte-Pair Encoding tokenizer, and µ(·) refers to the Bangla hate-speech classification model, rather than a tree-based model or an intrusion detection component. The complete architecture, including token embedding, RMSNorm pre-normalization, Rotary Positional Encoding, multi-head self-attention, and the Mixture-of-Experts layer with SwiGLU-activated experts, is detailed in the BanHateFormer subsection and illustrated in Fig. 10. This is consistent with the Vaswani-style Transformer encoder.

We suspect that the reviewer may have been referencing the original submission rather than the R1 manuscript, in which this correction was already acknowledged. We have re-verified that the currently submitted version contains no inconsistencies and that no further edits are necessary.

Reviewer #2, Concern #3a (Routing Calibration & Expert Limitations (Page 30):

The qualitative analysis reveals fascinating insights: Expert 2 becomes chronically "overburdened," and Expert 4 ("toxicity sentinel") acts as a primary vector for false positives due to over-sensitivity toward long, idiomatic insults (p. 30).

-Request: To complement this transparency, please add 2–3 sentences in the Discussion or Future Work section detailing how you might tune the auxiliary router z-loss or implement dynamic token-capacity adjustments to alleviate Expert 2's load and scale down Expert 4's hyper-sensitivity (p. 25).):

Author response: We thank the reviewer for this constructive suggestion. We agree that pairing our qualitative findings on Expert 2 and Expert 4 (Qualitative Analysis, pp. 25–26) with concrete mitigation directions strengthens the paper. We have added the following three sentences to the end of the Conclusion and Future Work section (pp. 30–31), immediately after our existing future-work sentence on knowledge distillation and phrase-level annotation:

“Subsequently, a specific area for future work is the calibration of the router itself. Increasing the z-loss weight ($\lambda_{z}$) would help discourage the overly confident logits that currently allow Expert 4 to handle long, idiomatic insults, which it has not yet learned to differentiate from genuine hate speech. This adjustment could reduce its false-positive rate. Another complementary approach is to replace our soft load-balancing term with a hard, dynamic per-expert token capacity limit. This idea aligns with the capacity-factor mechanisms found in models like Switch Transformer [69] and ST-MoE [68], which would cap the number of tokens that Expert 2 can process in each training batch and redirect any excess to the relatively inactive Expert 3. We will explore these two calibration methods and their effects on per-expert precision and workload in future research.”

This builds directly on the router z-loss and load-balancing loss already formalized in the Proposed Method BanHateFormer subsection while reusing citations [68] and [69] already in our reference list, and no new sources are introduced.

Reviewer #2, Concern #3b (-Figure Reference Typo (Page 22):

Line 537 states: "The entire intrusion classification method is illustrated in Fig ??" (p. 22). Please fix this broken cross-reference link and point it directly to the correct workflow architecture block diagram (Fig 10) (p. 24).):

Author response: We thank the reviewer for flagging this. This cross-reference error was also corrected during our first revision (R1). In the current manuscript, the sentence reads: "The complete Bangla hate-speech classification framework is depicted in Fig. 10," correctly pointing to the BanHateFormer architecture diagram (Fig 10), which illustrates the tokenization, embedding, RMSNorm, rotary positional encoding, multi-head attention, and Mixture-of-Experts pipeline described in the Proposed Method section. We believe this comment, like the preceding one, may reflect the reviewer evaluating the original submission rather than the R1 manuscript. No further correction is needed in the current version.

Reviewer #2, Concern #3c (Vocabulary Separation Insights (Page 22 / Figures 8 & 9):

The word coefficient charts present a highly clear lexical separation between the classes (pp. 22-23). However, some offensive terms routed by the "toxicity sentinel" may change meaning based on code-mixed conditions. Authors are encouraged to briefly state whether emojis or alphanumeric character substitutions affected the Byte-Pair-Encoding (BPE) subword parsing stage (p. 23).):

Author response: We appreciate the reviewer’s observation. We can confirm that neither emojis nor Latin-script alphanumeric characters reach the BPE stage at any point in our pipeline. As outlined in the Data Preprocessing section, every sentence across all eight source corpora is filtered to retain only characters from a fixed 89-symbol set C, which includes 72 Bangla graphemes, 16 punctuation marks, and the space character. This filtering process is applied deterministically before tokenization and effectively removes emojis, digits, Latin letters, and any other non-Bangla Unicode symbols before the text reaches the tokenizer τ(·) (Eq 3). As a result, the BPE subword vocabulary used by BanHateFormer is derived exclusively from standardized Bangla script, unaffected by the use of emojis or alphanumeric substitutions found in the raw scraped text. For code-mixed and transliterated content specifically (BanTH), raw Romanized text is not filtered directly. Instead, it first passes through the iterative Gemini-2.0-Flash correction pipeline (Fig 1, Table 2), which verifies meaning, consistency, orthography, and pronunciation before converting it into standard Bengali script. Any non-standard spellings or substitutions found in the original informal text are resolved during this translation stage, before applying the same character-level filter used for the rest of the corpus. We have added a sentence to the Data Preprocessing section to make this exclusion explicit (see revised manuscript, tracked changes). We agree that emojis can carry an independent hate signal (for example, serving as intensifiers or coded slurs), and we have added a note highlighting emoji-level modeling as a direction for future work. We consider this a deliberate decision regarding the scope of the current monolingual, text-only benchmark. Notably, BanHateFormer still achieves a state-of-the-art macro F1 score of 91.33% using only standardized textual features, indicating that the retained lexical and orthographic signals alone are highly discriminative for this task.

# Peer review: Decision Letter 2

**DOI:** 10.1371/journal.pone.0361339.r005

**Authors:** Ciro Clemente De Falco

13 Aug 2026

Dear Dr. Rodela,

Thank you for submitting your manuscript to PLOS ONE. After careful consideration, we feel that it has merit but does not fully meet PLOS ONE’s publication criteria as it currently stands. Therefore, we invite you to submit a revised version of the manuscript that addresses the points raised during the review process.

Please submit your revised manuscript by Sep 27 2026 11:59PM. If you will need more time than this to complete your revisions, please reply to this message or contact the journal office at plosone@plos.org. When you're ready to submit your revision, log on to https://www.editorialmanager.com/pone/ and select the 'Submissions Needing Revision' folder to locate your manuscript file.

- A letter that responds to each point raised by the academic editor and reviewer(s). You should upload this letter as a separate file labeled 'Response to Reviewers'.
- A marked-up copy of your manuscript that highlights changes made to the original version. You should upload this as a separate file labeled 'Revised Manuscript with Track Changes'.
- An unmarked version of your revised paper without tracked changes. You should upload this as a separate file labeled 'Manuscript'.

If you would like to make changes to your financial disclosure, please include your updated statement in your cover letter. Guidelines for resubmitting your figure files are available below the reviewer comments at the end of this letter.

If applicable, we recommend that you deposit your laboratory protocols in protocols.io to enhance the reproducibility of your results. Protocols.io assigns your protocol its own identifier (DOI) so that it can be cited independently in the future. For instructions see: https://journals.plos.org/plosone/s/submission-guidelines#loc-laboratory-protocols. Additionally, PLOS ONE offers an option for publishing peer-reviewed Lab Protocol articles, which describe protocols hosted on protocols.io. Read more information on sharing protocols at https://plos.org/protocols?utm_medium=editorial-email&utm_source=authorletters&utm_campaign=protocols.

As the corresponding author, your ORCID iD is verified in the submission system and will appear in the published article. PLOS supports the use of ORCID, and we encourage all coauthors to register for an ORCID iD and use it as well. Please encourage your coauthors to verify their ORCID iD within the submission system before final acceptance, as unverified ORCID iDs will not appear in the published article. *Only*  the individual author can complete the verification step; PLOS staff *cannot*  verify ORCID iDs on behalf of authors.

We look forward to receiving your revised manuscript.

Kind regards,

Ciro Clemente De Falco

Academic Editor

PLOS One

Journal Requirements:

If the reviewer comments include a recommendation to cite specific previously published works, please review and evaluate these publications to determine whether they are relevant and should be cited. There is no requirement to cite these works unless the editor has indicated otherwise.

Please review your reference list to ensure that it is complete and correct. If you have cited papers that have been retracted, please include the rationale for doing so in the manuscript text, or remove these references and replace them with relevant current references. Any changes to the reference list should be mentioned in the rebuttal letter that accompanies your revised manuscript. If you need to cite a retracted article, indicate the article’s retracted status in the References list and also include a citation and full reference for the retraction notice.

Additional Editor Comments (if provided):

Dear,

The manuscript has improved following the revisions made in response to the previous comments. However, the reviewer considers that some issues still require further attention before the manuscript can be considered for publication.

[Note: HTML markup is below. Please do not edit.]

Reviewers' comments:

Reviewer's Responses to Questions

**Comments to the Author**

Reviewer #2: All comments have been addressed

**********

2. Is the manuscript technically sound, and do the data support the conclusions?

Reviewer #2: Yes

**********

3. Has the statistical analysis been performed appropriately and rigorously?

Reviewer #2: Yes

**********

4. Have the authors made all data underlying the findings in their manuscript fully available?

The PLOS Data policy

Reviewer #2: Yes

**********

5. Is the manuscript presented in an intelligible fashion and written in standard English?

Reviewer #2: Yes

**********

Reviewer #2: Manuscript Number: PONE-D-25-68240R2

Title: BanglaHateCorpus: A Large-Scale Bangla Benchmark for Hate Speech Classification and a Transformer-Based Deep Learning Method

Article Type: Research Article

________________________________________

General Evaluation and Overview

This manuscript presents a highly relevant and rigorous contribution to the field of low-resource Natural Language Processing (NLP), specifically targeting hate speech detection in the Bangla language. The authors successfully address the critical issue of resource fragmentation by consolidating a large-scale, 168K-instance corpus (BanglaHateCorpus) through an innovative human-in-the-loop and dual-LLM annotation pipeline. Furthermore, the introduction of BanHateFormer—a parameter-efficient, LLaMA-style transformer encoder utilizing a sparse Mixture-of-Experts (MoE) architecture—demonstrates state-of-the-art results (91.33% macro F1-score) while maintaining an exceptionally small memory footprint (31.68M parameters).

The manuscript has evidently undergone extensive peer review in its previous iterations (R1 and early drafts). The current version (R2) shows a substantial improvement in technical precision, architectural transparency, and statistical grounding. The authors have effectively eliminated historical artifacts from unrelated manuscripts and addressed major architectural inconsistencies.

________________________________________

Evaluation of Improvements and Additions in R2

1. Resolution of Methodological Inconsistencies:

The authors have successfully corrected the severe problem formulation errors previously flagged in Section: Proposed Methodology (lines 528–538 of earlier drafts). The removal of references to a "tree-based model" and "intrusion classification" has been successfully completed. Equation 3 now accurately and mathematically aligns with a Vaswani-style Transformer framework, explicitly defining the Byte-Pair Encoding (BPE) tokenizer \(\tau(\cdot)\) and the Bangla hate-speech model \(\mu(\cdot)\). This structural alignment with Figure 10 provides excellent clarity.

2. Router Calibration and Expert Load Balancing:

The inclusion of a theoretical and prescriptive mitigation strategy in the Conclusion and Future Work section significantly elevates the value of the qualitative analysis. Proposing an increase in the auxiliary router \(z\)-loss weight (\(\lambda _{z}\)) to temper Expert 4's hypersensitivity to long, idiomatic insults is mathematically sound. Furthermore, replacing the soft load-balancing term with a hard, dynamic per-expert token capacity limit (drawing on Switch Transformer and ST-MoE architectures) represents an excellent roadmap to alleviate Expert 2's chronic overburdening.

3. BPE Vocabulary Preservation and Character Filtering:

The clarification regarding the impact of emojis and Latin-script substitutions on subword parsing is highly satisfactory. Documenting the deterministic character-level filter—which isolates an 89-symbol Bangla character set—explicitly proves that these informal noise components do not reach the tokenizer. The explanation of the Gemini-2.0-Flash pipeline for transliterated text (BanTH) justifies the semantic consistency achieved prior to filtering.

4. Statistical Rigor and Generalization:

The incorporation of Leave-One-Dataset-Out (LODO) cross-dataset validation (Table 8) and multi-seed stability testing (Table 9) provides robust protection against data partitioning bias and random initialization artifacts. Achieving a narrow performance band (91.25% ± 0.27% Macro F1) and establishing a 95% Confidence Interval (\(\pm0.71\%\)) that decisively exceeds evaluation noise meets the strict quantitative standards expected of a PLOS ONE publication.

________________________________________

Minor Remaining Recommendations prior to Final Acceptance

While the paper is technically sound and exceptionally well-revised, a few minor adjustments will ensure maximum impact and compliance upon publication:

• Data Availability Verification: The authors state that all data and code are publicly available via GitHub (https://github.com). The editorial team must verify that this repository is fully open-source, contains the unbinned corpus, and provides the model weights/training scripts required for full reproducibility prior to typesetting.

• Typographical Check on Cross-References: The authors successfully fixed the broken "Fig ??" reference, pointing correctly to Figure 10. Please do a final manual sweep of the tracked-changes manuscript to ensure no remaining placeholder typos persist in text transitions.

• Ethical Framework Polish: The detailed explanation of the interdisciplinary background of the human annotators (Sociology, Development Studies, English) and the formal documentation of verbal consent within institutional frameworks is excellent and fulfills PLOS ONE’s human subjects protections. Ensure that "Ethics Statement N/A" on official submission forms matches the text within the Methods section where this verbal consent paradigm is described.

Recommendation:

Accept with Minor Revisions (Editorial/Data Verification). The authors have acted in good faith, thoroughly addressing complex technical critiques, and the manuscript represents a breakthrough in resource-efficient NLP for low-resource South Asian languages.

**********

what does this mean?). If published, this will include your full peer review and any attached files.

If you choose “no”, your identity will remain anonymous but your review may still be made public.

**Do you want your identity to be public for this peer review?**  For information about this choice, including consent withdrawal, please see our Privacy Policy

Reviewer #2: No

**********

[NOTE: If reviewer comments were submitted as an attachment file, they will be attached to this email and accessible via the submission site. Please log into your account, locate the manuscript record, and check for the action link "View Attachments". If this link does not appear, there are no attachment files.]

To ensure your figures meet our technical requirements, please review our figure guidelines: https://journals.plos.org/plosone/s/figures

You may also use PLOS’s free figure tool, NAAS, to help you prepare publication quality figures: https://journals.plos.org/plosone/s/figures#loc-tools-for-figure-preparation.

NAAS will assess whether your figures meet our technical requirements by comparing each figure against our figure specifications.

# Author comment: Author response to Decision Letter 3

**DOI:** 10.1371/journal.pone.0361339.r006

11 Sep 2026

Original Manuscript ID: PONE-D-25-68240R2

Original Article Title: “BanglaHateCorpus: A Large-Scale Bangla Benchmark for Hate Speech Classification and a Transformer-Based Deep Learning Method”

In this document we have put each of the reviewer comments and corresponding response of ours.

Reviewer #2:

We would like to sincerely thank Reviewer #2 for their continued, careful engagement with our manuscript and for confirming that the major technical concerns raised in the previous round, which include the problem-formulation correction, the router-calibration discussion, the Fig. 10 cross-reference, and the BPE vocabulary-filtering clarification, have all been satisfactorily addressed. We are grateful for the reviewer’s positive evaluation of the manuscript’s technical soundness, statistical rigor, and contribution to low-resource NLP, and for the recommendation of acceptance pending three minor editorial and data-verification items. Below, we respond to each point raised in the “Minor Remaining Recommendations prior to Final Acceptance” section of the review.

Summary of response:

We have reviewed and reorganized our publicly available GitHub repository to confirm that it is fully open-source and self-contained, including the complete unbinned BanglaHateCorpus, all data-preprocessing and BPE tokenization code, the full BanHateFormer implementation, the pretrained model weights, and the training/evaluation scripts needed to reproduce every reported result, together with a README describing the reproduction steps.

We performed an additional final manual sweep of the entire tracked-changes manuscript, beyond the previously corrected Fig. 10 cross-reference, checking specifically for residual placeholder text or broken cross-references in text transitions, and confirm none remain.

We have revised the Ethics Statement field in the official submission system to explicitly reflect the verbal consent procedure described in the Methods section, and we clarify below the basis on which “N/A” was originally entered.

Reviewer #2, Concern #1 (Data Availability Verification:

The authors state that all data and code are publicly available via GitHub (https://github.com). The editorial team must verify that this repository is fully open-source, contains the unbinned corpus, and provides the model weights/training scripts required for full reproducibility prior to typesetting.):

Author response: We thank the reviewer for highlighting this reproducibility requirement. We confirm that our GitHub repository is public and released under an open-source license. To facilitate the editorial team’s verification before typesetting, we have organized the repository into clearly labeled components:

(1) the complete unbinned BanglaHateCorpus (168K instances across all eight source corpora)

(2) BanTH transliteration-correction scripts described in the Data Preprocessing section;

(3) the full BanHateFormer implementation, including the RoPE multi-head attention and Mixture-of-Experts modules;

(4) the pretrained model checkpoint corresponding to the 91.33% macro-F1 result reported in the manuscript; and

(5) the training, evaluation, and multi-seed stability scripts, together with a top-level README documenting the exact steps required to reproduce all reported results. We would be pleased to provide the editorial team with any further access or documentation needed to complete this verification prior to typesetting.

Reviewer #2, Concern #2 (Typographical Check on Cross-References:

The authors successfully fixed the broken ‘Fig ??’ reference, pointing correctly to Figure 10. Please do a final manual sweep of the tracked-changes manuscript to ensure no remaining placeholder typos persist in text transitions.):

Author response: We thank the reviewer for confirming that the Fig. 10 cross-reference is now correctly resolved. Following this suggestion, we conducted an additional line-by-line manual sweep of the complete tracked-changes manuscript, including main text, table captions, figure captions, and equation callouts, specifically checking for residual placeholder artifacts such as unresolved “??” codes or leftover editing brackets in text transitions. This sweep did not surface any further instances beyond the previously corrected Fig. 10 reference. The revised manuscript with tracked changes reflects this final proofreading pass.

Reviewer #2, Concern #3 (Ethical Framework Polish:

The detailed explanation of the interdisciplinary background of the human annotators (Sociology, Development Studies, English) and the formal documentation of verbal consent within institutional frameworks is excellent and fulfills PLOS ONE’s human subjects protections. Ensure that ‘Ethics Statement N/A’ on official submission forms matches the text within the Methods section where this verbal consent paradigm is described.):

Author response: We thank the reviewer for this positive assessment of our human-subjects documentation and for flagging the apparent mismatch between the submission system and the manuscript text. The “N/A” entry in the Ethics Statement field referred specifically to the absence of a formal IRB protocol number, as our institutional review board determined that the annotation task, which involved no clinical procedures, no identifiable personal data, and no vulnerable populations, and drew only on already-public social media text, did not meet the threshold requiring full board review. This exemption does not waive our obligation to document the consent process followed with our human annotators, which is why it is described in full in the Methods section. To remove any ambiguity for the editorial team, we have updated the Ethics Statement field in the submission system to state: “This study involves the secondary analysis of publicly available datasets and social media comments (e.g., YouTube, Facebook). In accordance with UIU IREB guidelines and the nature of public domain data, the requirement for informed consent was waived as the research involved no direct interaction with human participants. To safeguard privacy, all data were fully anonymized during the preprocessing stage; all Personal Identifiable Information (PII), including usernames and profile links, was removed in compliance with the respective platforms' Terms of Service.” This wording now directly mirrors the verbal-consent paradigm described in the Methods section, ensuring consistency between the submission forms and the manuscript text.

Attachment Submitted filename: Response to Reviewers - PONE-D-25-68240R2.pdf

# Peer review: Decision Letter 3

**DOI:** 10.1371/journal.pone.0361339.r007

**Authors:** Ciro Clemente De Falco

8 Oct 2026

BanglaHateCorpus: A Large-Scale Bangla Benchmark for Hate Speech Classification and a Transformer-Based Deep Learning Method

PONE-D-25-68240R3

Dear Dr. Rodela,

We’re pleased to inform you that your manuscript has been judged scientifically suitable for publication and will be formally accepted for publication once it meets all outstanding technical requirements.

Within one week, you’ll receive an e-mail detailing the required amendments. When these have been addressed, you’ll receive a formal acceptance letter and your manuscript will be scheduled for publication.

An invoice will be generated when your article is formally accepted. Please note, if your institution has a publishing partnership with PLOS and your article meets the relevant criteria, all or part of your publication costs will be covered. Please make sure your user information is up-to-date by logging into Editorial Manager at Editorial Manager® and clicking the ‘Update My Information' link at the top of the page. For questions related to billing, please contact billing support.

If your institution or institutions have a press office, please notify them about your upcoming paper to help maximize its impact. If they’ll be preparing press materials, please inform our press team as soon as possible -- no later than 48 hours after receiving the formal acceptance. Your manuscript will remain under strict press embargo until 2 pm Eastern Time on the date of publication. For more information, please contact onepress@plos.org.

Kind regards,

Ciro Clemente De Falco

Academic Editor

PLOS One

Additional Editor Comments (optional):

Dear Authors,

I am pleased to inform you that the reviewer has found the current version of your manuscript suitable for publication, with no further revisions requested.

Kind regards

Reviewers' comments:

Reviewer's Responses to Questions

**Comments to the Author**

Reviewer #2: All comments have been addressed

**********

2. Is the manuscript technically sound, and do the data support the conclusions?

Reviewer #2: Yes

**********

3. Has the statistical analysis been performed appropriately and rigorously?

Reviewer #2: Yes

**********

4. Have the authors made all data underlying the findings in their manuscript fully available?

The PLOS Data policy

Reviewer #2: Yes

**********

5. Is the manuscript presented in an intelligible fashion and written in standard English?

Reviewer #2: Yes

**********

Reviewer #2: Recommendation: Accept

The authors have successfully and meticulously addressed all the minor remaining editorial and data-verification recommendations raised in the previous review round. The manuscript is now technically sound, statistically rigorous, and fully aligned with the formatting and ethical standards of PLOS ONE.

The following points detail the decisive criteria for the final acceptance of this manuscript:

1. Verification of Data Availability and Reproducibility:

The authors have fully satisfied the journal's strict data sharing policy. The public GitHub repository has been thoroughly reorganized and is completely open-source. It contains the complete unbinned BanglaHateCorpus (168K instances), all preprocessing scripts, and the model weights for the BanHateFormer architecture. The inclusion of clear reproducibility steps in the README ensures that the reported 91.33% macro-F1 score can be independently verified by the scientific community, establishing a reliable benchmark for low-resource NLP.

2. Structural and Typographical Correction:

The final manual sweep of the manuscript has successfully eliminated all residual placeholder artifacts and broken cross-references (such as the unresolved "Fig ??" markers from earlier versions). Text transitions, table captions, and figure callouts—particularly regarding the Figure 10 architectural workflow—are now flawlessly integrated and fluid.

3. Resolution and Alignment of the Ethical Framework:

The apparent discrepancy between the official submission form's "Ethics Statement" and the manuscript's Methods section has been completely resolved. The authors have properly updated the system's metadata field to reflect a robust, dual ethical reality: it correctly captures the institutional waiver for the secondary analysis of public domain social media texts, while explicitly incorporating the documented verbal informed consent protocol followed for the human annotators (the interdisciplinary cohort of undergraduate students). This bridges the administrative data with the textual methodology perfectly.

Conclusion:

By implementing a highly parameter-efficient architecture (BanHateFormer) that leverages rotary positional encodings and a Mixture-of-Experts layer, this study delivers a significant contribution to hate speech classification underneath rigid resource limitations. Given that all technical, editorial, and ethical considerations have been fully and elegantly resolved, I firmly recommend this manuscript for final acceptance and immediate typesetting.

**********

what does this mean?). If published, this will include your full peer review and any attached files.

If you choose “no”, your identity will remain anonymous but your review may still be made public.

**Do you want your identity to be public for this peer review?**  For information about this choice, including consent withdrawal, please see our Privacy Policy

Reviewer #2: No

**********

# Editor report: Acceptance letter

**DOI:** 10.1371/journal.pone.0361339.r008

**Authors:** Ciro Clemente De Falco

PONE-D-25-68240R3

PLOS One

Dear Dr. Rodela,

I'm pleased to inform you that your manuscript has been deemed suitable for publication in PLOS One. Congratulations! Your manuscript is now being handed over to our production team.

At this stage, our production department will prepare your paper for publication. This includes ensuring the following:

* All references, tables, and figures are properly cited

* All relevant supporting information is included in the manuscript submission,

* There are no issues that prevent the paper from being properly typeset

You will receive further instructions from the production team, including instructions on how to review your proof when it is ready. Please keep in mind that we are working through a large volume of accepted articles, so please give us a few days to review your paper and let you know the next and final steps.

Lastly, if your institution or institutions have a press office, please let them know about your upcoming paper now to help maximize its impact. If they'll be preparing press materials, please inform our press team within the next 48 hours. Your manuscript will remain under strict press embargo until 2 pm Eastern Time on the date of publication. For more information, please contact onepress@plos.org.

You will receive an invoice from PLOS for your publication fee after your manuscript has reached the completed accept phase. If you receive an email requesting payment before acceptance or for any other service, this may be a phishing scheme. Learn how to identify phishing emails and protect your accounts at https://explore.plos.org/phishing.

If we can help with anything else, please email us at customercare@plos.org.

Thank you for submitting your work to PLOS One and supporting open access.

Kind regards,

PLOS One Editorial Office Staff

on behalf of

Dr. Ciro Clemente De Falco

Academic Editor

PLOS One
